# Supplementary material for: Estimating genetic variability among diverse lentil collections through novel multivariate techniques
Source: PLoS One. 2022 Jun 30;17(6):e0269177. doi: 10.1371/journal.pone.0269177 (PMC9246128; doi:10.1371/journal.pone.0269177)
Supplement: S1 Fig — (DOCX) [file pone.0269177.s001.docx]

**S1 Fig. Average temperature during lentil crop season October to April 2017-18 and 2018-19 at the experimental station**
