# Supplementary material for: Estimating genetic variability among diverse lentil collections through novel multivariate techniques
Source: PLoS One. 2022 Jun 30;17(6):e0269177. doi: 10.1371/journal.pone.0269177 (PMC9246128; doi:10.1371/journal.pone.0269177)
Supplement: S1 Table — (DOCX) [file pone.0269177.s002.docx]

**S1 Table. List of lentil genotypes studied during 2017-18 and 2018-19. Two varieties Markaz 2009 and Punjab 2009 were used as checks.**

| **S. No.** | **Genotypes** | **Origin** | | **S. No.** | | **Genotypes** | | **Origin** | | **S. No.** | | **Genotypes** | | **Origin** | | **S. No.** | | **Genotypes** | | **Origin** | | **S. No.** | | **Genotypes** | | Origin |
| --- | --- | --- | --- | --- | --- | --- | --- | --- | --- | --- | --- | --- | --- | --- | --- | --- | --- | --- | --- | --- | --- | --- | --- | --- | --- | --- |
| 1 | 5472 | | Sialkot | | 24 | | 5511 | | Muzaffargarh | | 47 | | 5571 | | Jacobabad | | 70 | | 5636 | | Layyah | | 93 | | 5669 | Layyah |
| 2 | 5474 | | Gujranwala | | 25 | | 5512 | | Muzaffargarh | | 48 | | 5575 | | Ghotki | | 71 | | 5637 | | Hyderabad | | 94 | | 5670 | Layyah |
| 3 | 5475 | | Gujranwala | | 26 | | 5517 | | Muzaffargarh | | 49 | | 5576 | | Ghotki | | 72 | | 5638 | | Hyderabad | | 95 | | 5671 | Layyah |
| 4 | 5476 | | Gujranwala | | 27 | | 5518 | | Muzaffargarh | | 50 | | 5580 | | Muzaffargarh | | 73 | | 5639 | | Hyderabad | | 96 | | 5672 | Layyah |
| 5 | 5477 | | Gujranwala | | 28 | | 5527 | | Sialkot | | 51 | | 5581 | | Multan | | 74 | | 5640 | | Hyderabad | | 97 | | 5673 | Narowal |
| 6 | 5478 | | Sheikhupura | | 29 | | 5529 | | Kasur | | 52 | | 5583 | | Muzaffargarh | | 75 | | 5643 | | Thatta | | 98 | | 5677 | Narowal |
| 7 | 5479 | | Lahore | | 30 | | 5530 | | Muzaffargarh | | 53 | | 5584 | | Muzaffargarh | | 76 | | 5647 | | Thatta | | 99 | | 5677 | Narowal |
| 8 | 5480 | | Kasur | | 31 | | 5531 | | Muzaffargarh | | 54 | | 5590 | | Muzaffargarh | | 77 | | 5650 | | Thatta | | 100 | | 5679 | Narowal |
| 9 | 5481 | | Kasur | | 32 | | 5532 | | Jhang | | 55 | | 5593 | | Nawabshah | | 78 | | 5652 | | Hyderabad | | 101 | | 5680 | Narowal |
| 10 | 5482 | | Lahore | | 33 | | 5535 | | Jhang | | 56 | | 5595 | | Kharan | | 79 | | 5653 | | Layyah | | 102 | | 5683 | Narowal |
| 11 | 5483 | | Sheikhupura | | 34 | | 5537 | | Gujrat | | 57 | | 5598 | | Kharan | | 80 | | 5654 | | Layyah | | 103 | | 5684 | Narowal |
| 12 | 5484 | | Sheikhupura | | 35 | | 5538 | | Thatta | | 58 | | 5600 | | Punjgur | | 81 | | 5655 | | Bhakkar | | 104 | | 5685 | Narowal |
| 13 | 5485 | | Kasur | | 36 | | 5549 | | Badin | | 59 | | 5610 | | Khuzdar | | 82 | | 5656 | | Bhakkar | | 105 | | 5686 | Narowal |
| 14 | 5486 | | Kasur | | 37 | | 5550 | | Hyderabad | | 60 | | 5621 | | Faisalabad | | 83 | | 5657 | | Bhakkar | | 106 | | 5687 | Narowal |
| 15 | 5488 | | Okara | | 38 | | 5553 | | Hyderabad | | 61 | | 5622 | | Faisalabad | | 84 | | 5658 | | Bhakkar | | 107 | | 5688 | Sialkot |
| 16 | 5489 | | Okara | | 39 | | 5555 | | Hyderabad | | 62 | | 5623 | | Faisalabad | | 85 | | 5659 | | Bhakkar | | 108 | | 5689 | Sialkot |
| 17 | 5491 | | Sahiwal | | 40 | | 5556 | | Hyderabad | | 63 | | 5624 | | Rawalpindi | | 86 | | 5660 | | Bhakkar | | 109 | | 5689 | Sialkot |
| 18 | 5493 | | Sahiwal | | 41 | | 5561 | | Sanghar | | 64 | | 5625 | | Muzaffargarh | | 87 | | 5661 | | Bhakkar | | 110 | | 5690 | Sialkot |
| 19 | 5494 | | Okara | | 42 | | 5562 | | Sanghar | | 65 | | 5626 | | Muzaffargarh | | 88 | | 5664 | | Layyah | | 111 | | 5691 | Sialkot |
| 20 | 5500 | | Khanewal | | 43 | | 5563 | | Sanghar | | 66 | | 5628 | | Rajanpur | | 89 | | 5665 | | Layyah | | 112 | | 5692 | Sialkot |
| 21 | 5501 | | Khanewal | | 44 | | 5564 | | Sanghar | | 67 | | 5630 | | Rajanpur | | 90 | | 5666 | | Layyah | | 113 | | 5693 | Sialkot |
| 22 | 5506 | | Muzaffargarh | | 45 | | 5565 | | Hyderabad | | 68 | | 5634 | | Layyah | | 91 | | 5667 | | Layyah | | 114 | | 5694 | Sialkot |
| 23 | 5510 | | Muzaffargarh | | 46 | | 5570 | | Larkana | | 69 | | 5635 | | Larkana | | 92 | | 5668 | | Layyah | | 115 | | 5695 | Sialkot |
| 116 | 5696 | | Narowal | | 139 | | 5751 | | Jhelum | | 162 | | 6015 | | USA | | 185 | | 6075 | | Bahawalnagar | | 208 | | 6123 | Sheikhupura |
| 117 | 5698 | | Sialkot | | 140 | | 5753 | | Rawalpindi | | 163 | | 6017 | | USA | | 186 | | 6076 | | Bahawalnagar | | 209 | | 6124 | Sheikhupura |
| 118 | 5700 | | Sialkot | | 141 | | 5772 | | Panjgur | | 164 | | 6037 | | Syria | | 187 | | 6077 | | Bahawalnagar | | 210 | | 6125 | Narowal |
| 119 | 5712 | | Narowal | | 142 | | 5773 | | Kharan | | 165 | | 6038 | | Syria | | 188 | | 6078 | | Khanewal | | 211 | | 23776 | Pakistan |
| 120 | 5716 | | Narowal | | 143 | | 5856 | | USA | | 166 | | 6041 | | Syria | | 189 | | 6080 | | Narowal | | 212 | | 23777 | Pakistan |
| 121 | 5717 | | Gujranwala | | 144 | | 5861 | | USA | | 167 | | 6042 | | Syria | | 190 | | 6081 | | Narowal | | 213 | | 23779 | Pakistan |
| 122 | 5723 | | Layyah | | 145 | | 5979 | | USA | | 168 | | 6043 | | Syria | | 191 | | 6082 | | Narowal | | 214 | | 23781 | Pakistan |
| 123 | 5724 | | Layyah | | 146 | | 5981 | | USA | | 169 | | 6045 | | Syria | | 192 | | 6083 | | Narowal | | 215 | | 23787 | Unknown |
| 124 | 5726 | | Layyah | | 147 | | 5982 | | USA | | 170 | | 6046 | | Syria | | 193 | | 6084 | | Narowal | | 216 | | 24783 | Unknown |
| 125 | 5727 | | Layyah | | 148 | | 5988 | | USA | | 171 | | 6047 | | Syria | | 194 | | 6085 | | Narowal | | 217 | | 24784 | Unknown |
| 126 | 5729 | | Layyah | | 149 | | 5993 | | USA | | 172 | | 5856 | | USA | | 195 | | 6086 | | Narowal | | 218 | | 24785 | Unknown |
| 127 | 5730 | | Layyah | | 150 | | 5995 | | USA | | 173 | | 6052 | | Syria | | 196 | | 6087 | | Narowal | | 219 | | 24786 | Unknown |
| 128 | 5737 | | Chakwal | | 151 | | 5996 | | USA | | 174 | | 6054 | | Syria | | 197 | | 6089 | | Narowal | | 220 | | 24787 | Unknown |
| 129 | 5739 | | Muzaffargarh | | 152 | | 5999 | | USA | | 175 | | 6058 | | Syria | | 198 | | 6090 | | Narowal | |  | |  |  |
| 130 | 5741 | | Bahawalpur | | 153 | | 6002 | | USA | | 176 | | 6060 | | Syria | | 199 | | 6092 | | Rawalpindi | |  | |  |  |
| 131 | 5742 | | Bahawalpur | | 154 | | 6003 | | USA | | 177 | | 6062 | | Syria | | 200 | | 6093 | | Rawalpindi | |  | |  |  |
| 132 | 5744 | | Faisalabad | | 155 | | 6005 | | USA | | 178 | | 6064 | | Syria | | 201 | | 6097 | | Chakwal | |  | |  |  |
| 133 | 5745 | | Faisalabad | | 156 | | 6008 | | USA | | 179 | | 6066 | | Bahawalpur | | 202 | | 6099 | | Rawalpindi | |  | |  |  |
| 134 | 5746 | | Lahore | | 157 | | 6010 | | USA | | 180 | | 6067 | | Bahawalpur | | 203 | | 6101 | | Rawalpindi | |  | |  |  |
| 135 | 5747 | | Kasur | | 158 | | 6011 | | USA | | 181 | | 6068 | | Bahawalpur | | 204 | | 6104 | | Rawalpindi | |  | |  |  |
| 136 | 5748 | | Gujranwala | | 159 | | 6012 | | USA | | 182 | | 6069 | | Bahawalpur | | 205 | | 6114 | | Bahawalnagar | |  | |  |  |
| 137 | 5749 | | Gujranwala | | 160 | | 6013 | | USA | | 183 | | 6073 | | Bahawalnagar | | 206 | | 6116 | | Bahawalnagar | |  | |  |  |
| 138 | 5750 | | Jhelum | | 161 | | 6014 | | USA | | 184 | | 6074 | | Bahawalnagar | | 207 | | 6122 | | Sheikhupura | |  | |  |  |
