# Supplementary material for: Estimating genetic variability among diverse lentil collections through novel multivariate techniques
Source: PLoS One. 2022 Jun 30;17(6):e0269177. doi: 10.1371/journal.pone.0269177 (PMC9246128; doi:10.1371/journal.pone.0269177)
Supplement: S2 Table — (DOCX) [file pone.0269177.s003.docx]

**S2 Table. Mean±SE for quantitative traits of 5% best performing lentil genotypes during 2017-18.**

| Geno | SY (g) | Geno | 100-SW (g) | Geno | BY (g) | Geno | PH (cm) | Geno | LPH (cm) | Geno | PS (cm) |
| --- | --- | --- | --- | --- | --- | --- | --- | --- | --- | --- | --- |
| 6084 | 85± 0.07 | 6052 | 3.65±0.0 | 6122 | 264±2.8 | 5593 | 71.12±1.7 | 5527 | 32.16±5.2 | 6010 | 0.74±0.1 |
| 6062 | 73±1.0 | 5583 | 3.64±0.0 | 5689 | 258±1.0 | 5549 | 65.1± | 6062 | 24.3±1.0 | 6013 | 0.66±0.02 |
| 6122 | 71±1.9 | 5556 | 2.97±0.0 | 5730 | 254±1.5 | 5694 | 62.86±2.1 | 6013 | 20.92±1.2 | 6041 | 0.66±0.02 |
| 6058 | 67±1.0 | 6101 | 2.9±0.0 | 6037 | 254±2.0 | 5687 | 60.8±3.7 | 6041 | 20.92±1.3 | 23776 | 0.62±0.03 |
| 6087 | 67±0.7 | 6124 | 2.88±0.0 | 6042 | 246±1.3 | 5518 | 58.64±2.4 | 6042 | 20.32±2.0 | 6124 | 0.62±0.03 |
| 5689 | 66±0.9 | 5684 | 2.82±0.0 | 5600 | 245±0.7 | 5472 | 58.14±2.4 | 5518 | 20.3±0.7 | 6122 | 0.61±0.01 |
| 6042 | 63±2.0 | 5856 | 2.56±0.0 | 5653 | 232±1.4 | 5622 | 57.98±6.5 | 5686 | 19.74±1.0 | 6043 | 0.60±0.02 |
| 6074 | 62±1.1 | 23776 | 2.48±0.0 | 5982 | 220±1.3 | 5500 | 57.52±2.1 | 5993 | 19.18±1.1 | 5996 | 0.6±0.04 |
| 5664 | 59±1.6 | 6054 | 2.47±0.0 | 5658 | 215±1.6 | 5506 | 57.52±2.1 | 5529 | 19.12±1.3 | 6069 | 0.58±0.03 |
| 5687 | 58±0.7 | 5643 | 2.46±0.0 | 6084 | 210±1.3 | 5723 | 57±2.2 | 6002 | 18.52±0.7 | 6076 | 0.58±0.05 |
| Markaz | 41.72±1.0 | Markaz | 1.91±0.0 | Markaz | 87.5±1.8 | Markaz | 59.09±1.4 | Markaz | 15.32±0.4 | Markaz | 1.55±0.20 |
| Punjab | 55.27±2.4 | Punjab | 2.48±0.0 | Punjab | 112.2±6.39 | Punjab | 55.1±1.4 | Punjab | 15.6±0.36 | Punjab | 1.36±0.01 |
| Geno | NSP (n) | Geno | DM | Geno | CT (mint) | Geno | HS(n) | Geno | NP |  |  |
| 6074 | 3±0.0 | 5698 | 161±0.0 | 6074 | 10±0.0 | 6074 | 0±0.0 | 5995 | 3±0.0 |  |  |
| 5595 | 3±0.0 | 6015 | 161±0.0 | 5595 | 10±0.0 | 5595 | 0±0.0 | 5861 | 3±0.0 |  |  |
| 6075 | 3±0.0 | 5667 | 172±0.0 | 6075 | 11±0.0 | 6075 | 0±0.0 | 5691 | 3±0.0 |  |  |
| 6010 | 2±0.0 | 5748 | 172±0.0 | 6010 | 11±0.0 | 6010 | 0±0.0 | 5671 | 3±0.0 |  |  |
| 6013 | 2±0.0 | 5555 | 172±0.0 | 6013 | 11±0.0 | 6041 | 0±0.0 | 6045 | 3±0.0 |  |  |
| 6041 | 2±0.0 | 6017 | 173±0.0 | 6041 | 11±0.0 | 23776 | 0±0.0 | 5689 | 3±0.0 |  |  |
| 23776 | 2±0.0 | 5700 | 173±0.0 | 23776 | 11±0.0 | 6124 | 0±0.0 | 5537 | 3±0.0 |  |  |
| 6124 | 2±0.0 | 5571 | 174±0.0 | 6124 | 11±0.0 | 6122 | 0±0.0 | 5685 | 3±0.0 |  |  |
| 6122 | 2±0.0 | 5580 | 175±0.0 | 6122 | 11±0.0 | 6043 | 0±0.0 | 6047 | 3±0.0 |  |  |
| 6043 | 2±0.0 | 5981 | 178±0.0 | 6043 | 12±0.0 | 5996 | 0±0.0 | 6074 | 2±0.0 |  |  |
| Markaz | 2±0.0 | Markaz | 172±0.29 | Markaz | 12.09±0.1 | Markaz | 0.27±0.1 | Markaz | 3±0.0 |  |  |
| Punjab | 2±0.0 | Punjab | 170±0.29 | Punjab | 11.7±0.19 | Punjab | 0.25±0.17 | Punjab | 3±0.0 |  |  |

Geno, Genotype; SY, seed yield; 100-SW, hundred seed weight; BY, biological yield; PH, plant height; LPH, lower pod height; PS, pod size; NSP, number of seed per pod; DM, days to maturity; CT, cooking time; HS, hard seed; NP, number of pods; g, gram; cm, centimeters; n, number; mint, minutes.
