# Supplementary material for: Estimating genetic variability among diverse lentil collections through novel multivariate techniques
Source: PLoS One. 2022 Jun 30;17(6):e0269177. doi: 10.1371/journal.pone.0269177 (PMC9246128; doi:10.1371/journal.pone.0269177)
Supplement: S3 Table — (DOCX) [file pone.0269177.s004.docx]

**S3 Table. Mean±SE for quantitative traits of 5% best performing lentil genotypes during 2018-19.**

| Geno | SY (g) | Geno | HSW (g) | Geno | BY (g) | Geno | PH (cm) | Geno | LPH (cm) | Geno | PS (cm) |
| --- | --- | --- | --- | --- | --- | --- | --- | --- | --- | --- | --- |
| 6084 | 76±2.9 | 5583 | 3.2±0.0 | 6042 | 187±1.3 | 6062 | 69±2.1 | 6099 | 33.34±2.1 | 5550 | 0.74±1.1 |
| 6062 | 69±1.0 | 6052 | 3.2±0.0 | 6087 | 183±0.9 | 6067 | 66±0.7 | 5717 | 20.28±2.4 | 5556 | 0.66±2.1 |
| 6122 | 68±1.7 | 5684 | 2.71±0.0 | 5689 | 179±1.3 | 6060 | 65.8±1.4 | 5695 | 19.74±1.7 | 5570 | 0.66±0.8 |
| 6058 | 67±2.2 | 6062 | 2.4±0.0 | 6012 | 175±2.3 | 6074 | 65±1.7 | 6097 | 19.12±1.3 | 5667 | 0.62±1.8 |
| 6087 | 62±2.8 | 6101 | 2.4±0.0 | 6037 | 172±0.9 | 5485 | 65±1.7 | 5684 | 19.12±1.8 | 5665 | 0.62±2.3 |
| 5689 | 60±1.9 | 5556 | 2.36±0.0 | 6052 | 167±2.1 | 5562 | 65±2.1 | 6101 | 19.12±0.2 | 5661 | 0.612±1.9 |
| 6074 | 58±2.2 | 5643 | 2.31±0.0 | 6060 | 167±0.7 | 6104 | 64.4±2.7 | 6124 | 18.94±1.1 | 5575 | 0.6±0.8 |
| 6042 | 57±1.3 | 5553 | 2.31±0.0 | 24786 | 163±2.6 | 24783 | 64±1.8 | 6060 | 18.76±2.1 | 5531 | 0.6±1.9 |
| 5664 | 53±2.4 | 6086 | 2.3±0.0 | 24783 | 162±1.5 | 5538 | 63.2±1.8 | 5693 | 18.18±0.8 | 5628 | 0.58±1.5 |
| 5687 | 53±1.0 | 23777 | 2.2±0.0 | 6090 | 162±1.3 | 6064 | 63±1.9 | 5667 | 17.94±0.8 | 5623 | 0.58±1.9 |
| Markaz | 39.08±0.9 | Markaz | 1.85±0.01 | Markaz | 88.58±1.6 | Markaz | 61.58±1.1 | Markaz | 16.56±0.6 | Markaz | 1.33±0.01 |
| Punjab | 53.27±1.7 | Punjab | 2.4±0.1 | Punjab | 110.4±5.1 | Punjab | 56.54±0.9 | Punjab | 16.71±0.6 | Punjab | 1.37±0.01 |
| Geno | SP (n) | Geno | MD | Geno | CT (mint) | Geno | HS (n) | Geno | NP |  |  |
| 5550 | 3±0.0 | 6092 | 198±0.0 | 5745 | 10±0.0 | 5480 | 2±0.0 | 5595 | 3±0.0 |  |  |
| 5637 | 3±0.0 | 6099 | 198±0.0 | 5575 | 10±0.0 | 5532 | 2±0.0 | 6075 | 3±0.0 |  |  |
| 5625 | 3±0.0 | 5511 | 198±0.0 | 6087 | 11±0.0 | 23779 | 2±0.0 | 6074 | 3±0.0 |  |  |
| 5555 | 3±0.0 | 5518 | 198±0.0 | 6090 | 11±0.0 | 5742 | 2±0.0 | 5480 | 2±0.0 |  |  |
| 5677 | 3±0.0 | 5517 | 197±0.0 | 5576 | 11±0.0 | 5637 | 2±0.0 | 5532 | 2±0.0 |  |  |
| 5630 | 3±0.0 | 6097 | 197±0.0 | 6089 | 11±0.0 | 5700 | 2±0.0 | 23779 | 2±0.0 |  |  |
| 5538 | 3±0.0 | 6014 | 196±0.0 | 5727 | 11±0.0 | 5694 | 2±0.0 | 5742 | 2±0.0 |  |  |
| 5583 | 3±0.0 | 5628 | 196±0.0 | 5737 | 11±0.0 | 5993 | 2±0.0 | 5637 | 2±0.0 |  |  |
| 5654 | 3±0.0 | 5677 | 196±0.0 | 6092 | 11±0.0 | 5630 | 2±0.0 | 5700 | 2±0.0 |  |  |
| 5518 | 3±0.0 | 6082 | 195±0.0 | 6064 | 12±0.0 | 5549 | 2±0.0 | 5694 | 2±0.0 |  |  |
| Markaz | 2±0.0 | Markaz | 172.5±0.2 | Markaz | 12.16±0.1 | Markaz | 0.41±0.1 | Markaz | 3±0 |  |  |
| Punjab | 2±0.0 | Punjab | 169±0.3 | Punjab | 11.90±1.6 | Punjab | 0.09±0.9 | Punjab | 3±0 |  |  |

Geno, Genotype; SY, seed yield; HSW, hundred seed weight; BY, biological yield; PH, plant height; LPH, lower pod height; PS, pod size; NSP, number of seed per pod; DM, days to maturity; CT, cooking time; HS, hard seed; NP, number of pods; g, gram; cm, centimeters; n, number; mint, minutes.
