# Supplementary material for: Estimating genetic variability among diverse lentil collections through novel multivariate techniques
Source: PLoS One. 2022 Jun 30;17(6):e0269177. doi: 10.1371/journal.pone.0269177 (PMC9246128; doi:10.1371/journal.pone.0269177)
Supplement: S4 Table — (DOCX) [file pone.0269177.s005.docx]

**S4 Table. Principal component analysis for quantitative traits of lentils studied during 2017-18.**

|  | **F1** | **F2** | **F3** | **F4** | **F5** | **F6** | **F7** | **F8** | **F9** | **F10** | **F11** |
| --- | --- | --- | --- | --- | --- | --- | --- | --- | --- | --- | --- |
| Eigenvalue | 2.09 | 1.37 | 1.19 | 1.08 | 1.02 | 0.95 | 0.89 | 0.80 | 0.76 | 0.57 | 0.23 |
| Variability (%) | 19.03 | 12.50 | 10.88 | 9.90 | 9.34 | 8.63 | 8.09 | 7.31 | 6.91 | 5.19 | 2.17 |
| Cumulative % | 19.03 | 31.54 | 42.42 | 52.33 | 61.67 | 70.30 | 78.39 | 85.71 | 92.62 | 97.82 | 100 |
| Contribution of variables (%) | | | | | | | | | | | |
|  | F1 | F2 | F3 | F4 | F5 | F6 | F7 | F8 | F9 | F10 | F11 |
| SY | 35.15 | 6.56 | 0.06 | 0.56 | 0.50 | 0.24 | 0.03 | 0.69 | 0.20 | 5.30 | 50.65 |
| 100-SW | 4.89 | 23.41 | 1.73 | 2.17 | 0.00 | 10.01 | 2.33 | 41.90 | 0.48 | 12.89 | 0.15 |
| BY | 33.84 | 4.56 | 0.02 | 0.02 | 0.87 | 0.01 | 0.55 | 4.91 | 3.89 | 6.65 | 44.65 |
| PH | 2.07 | 15.62 | 20.24 | 4.11 | 2.06 | 0.53 | 7.19 | 8.68 | 38.17 | 0.24 | 1.05 |
| LPH | 0.33 | 3.01 | 0.00 | 51.58 | 2.12 | 4.49 | 25.60 | 6.59 | 5.44 | 0.24 | 0.56 |
| PS | 19.37 | 11.21 | 2.96 | 0.27 | 2.64 | 0.91 | 2.42 | 0.00 | 0.00 | 60.18 | 0.01 |
| NSP | 0.45 | 8.64 | 35.05 | 0.88 | 0.01 | 5.98 | 17.71 | 4.00 | 24.71 | 0.44 | 2.09 |
| DM | 1.10 | 7.98 | 2.06 | 16.08 | 16.97 | 20.89 | 28.48 | 0.43 | 1.71 | 4.22 | 0.03 |
| CT | 0.11 | 0.00 | 19.90 | 6.22 | 23.84 | 30.67 | 2.95 | 14.79 | 0.96 | 0.40 | 0.12 |
| HS | 0.25 | 13.26 | 11.02 | 15.05 | 0.02 | 24.41 | 7.63 | 9.61 | 17.83 | 0.39 | 0.50 |
| NP | 2.40 | 5.70 | 6.94 | 3.03 | 50.93 | 1.83 | 5.06 | 8.37 | 6.56 | 8.99 | 0.15 |

Geno, Genotype; SY, seed yield; 100-SW, hundred seed weight; BY, biological yield; PH, plant height; LPH, lower pod height; PS, pod size; NSP, number of seed per pod; DM, days to maturity; CT, cooking time; HS, hard seed; NP, number of pods, F, factors
