# Supplementary material for: Estimating genetic variability among diverse lentil collections through novel multivariate techniques
Source: PLoS One. 2022 Jun 30;17(6):e0269177. doi: 10.1371/journal.pone.0269177 (PMC9246128; doi:10.1371/journal.pone.0269177)
Supplement: S5 Table — (DOCX) [file pone.0269177.s006.docx]

**S5 Table. Principal Component Analysis for quantitative traits studied during 2018-19.**

|  | **F1** | **F2** | **F3** | **F4** | **F5** | **F6** | **F7** | **F8** | **F9** | **F10** | **F11** |  |
| --- | --- | --- | --- | --- | --- | --- | --- | --- | --- | --- | --- | --- |
| Eigenvalue | 2.19 | 1.34 | 1.15 | 1.11 | 1.03 | 0.95 | 0.92 | 0.78 | 0.69 | 0.65 | 0.15 |  |
| Variability (%) | 19.91 | 12.19 | 10.52 | 10.16 | 9.37 | 8.65 | 8.41 | 7.13 | 6.30 | 5.90 | 1.42 |  |
| Cumulative % | 19.91 | 32.10 | 42.62 | 52.78 | 62.15 | 70.81 | 79.22 | 86.36 | 92.66 | 98.57 | 100 |  |
| **Contribution of variables (%)** | | | | | | | | | | | |  |
|  | F1 | F2 | F3 | F4 | F5 | F6 | F7 | F8 | F9 | F10 | F11 |  |
| SY | 37.46 | 0.18 | 0.02 | 1.52 | 0.00 | 0.15 | 0.62 | 5.78 | 1.65 | 2.67 | 49.91 |  |
| HSW | 9.87 | 7.68 | 0.00 | 1.89 | 6.70 | 10.36 | 1.16 | 56.41 | 0.00 | 5.80 | 0.07 |  |
| BY | 36.51 | 2.26 | 0.20 | 0.43 | 0.02 | 0.03 | 0.09 | 6.02 | 3.70 | 1.79 | 48.90 |  |
| PH | 4.26 | 0.57 | 41.65 | 3.35 | 6.62 | 6.10 | 1.17 | 0.42 | 14.59 | 21.09 | 0.13 |  |
| LPH | 1.85 | 9.83 | 11.96 | 27.26 | 5.26 | 1.94 | 11.24 | 1.62 | 15.00 | 13.83 | 0.10 |  |
| PS | 2.12 | 11.95 | 9.81 | 0.45 | 1.20 | 6.99 | 53.47 | 4.28 | 8.67 | 1.03 | 0.00 |  |
| NSP | 1.93 | 14.37 | 1.09 | 13.19 | 17.28 | 26.95 | 0.44 | 1.27 | 8.20 | 15.23 | 0.00 |  |
| DM | 1.23 | 1.127 | 16.04 | 46.05 | 0.10 | 0.62 | 6.80 | 3.87 | 0.65 | 23.43 | 0.04 |  |
| CT | 3.54 | 31.77 | 0.97 | 0.95 | 0.06 | 0.02 | 17.92 | 7.31 | 24.94 | 11.64 | 0.83 |  |
| HS | 0.01 | 12.90 | 14.35 | 4.65 | 0.39 | 46.69 | 5.62 | 2.99 | 9.28 | 3.08 | 0.00 |  |
| NP | 1.16 | 7.32 | 3.86 | 0.23 | 62.32 | 0.11 | 1.43 | 9.97 | 13.21 | 0.36 | 0.00 |  |

Geno, Genotype; SY, seed yield; 100-SW, hundred seed weight; BY, biological yield; PH, plant height; LPH, lower pod height; PS, pod size; NSP, number of seed per pod; DM, days to maturity; CT, cooking time; HS, hard seed; NP, number of pods, F, factors
