# Supplementary material for: Estimating genetic variability among diverse lentil collections through novel multivariate techniques
Source: PLoS One. 2022 Jun 30;17(6):e0269177. doi: 10.1371/journal.pone.0269177 (PMC9246128; doi:10.1371/journal.pone.0269177)
Supplement: Attachment — Submitted filename: PONE-D-21-20067_R3.pdf [file pone.0269177.s011.pdf]

PLOS ONE

Estimating genetic variability among diverse lentil collections through novel multivariate techniques

--Manuscript Draft--

Manuscript Number: PONE-D-21-20067R3

Article Type: Research Article

Full Title: Estimating genetic variability among diverse lentil collections through novel multivariate techniques

Short Title: Estimating genetic variability in lentils

Corresponding Author: Muhammad Sajjad Iqbal, PhD
University of Gujrat - Hafiz Hayat Campus: University of Gujrat
Gujrat, Punjab PAKISTAN

Keywords: Lentil, genetic variability, multivariate analysis, cooking time, seed yield

Abstract: <p>Lentil is an important food legume throughout the world and Pakistan stands at 18th position with 8,610 tons production from 17,457 hectares. It is rich in protein, carbohydrates, fat, fiber, and minerals that can potentially meet food security and resolve malnutrition issues, particularly in South Asia. Two hundred and twenty lentil genotypes representing Pakistan (178), Syria (14), and USA (22) including 6 from unknown origin were studied for yield, yield contributing traits, and cooking time (CT). The genotype 6122 (Pakistan) performed the best during both years with seed yield per plant (SY) 68±1.7 g, biological yield per plant (BY) 264±2.8 g, pod size (PS) 0.61±0.01 cm, number of seeds per pod (SP) 2, cooking time (CT) 11 minutes, with no hard seed (HS). The genotype 6122 (Pakistan) and 6042 (Syria) produced the highest BY, hence these have potential as an efficient source for fodder, particularly during extreme winter months. The genotype 5698 (Pakistan) and 6015 (USA) were late in maturity, during 2018-19 while 24783 and 5561 matured early in 2019. Minimum CT 10 minutes was taken by the genotypes 6074 and 5745 of Pakistani origin. The lowest CT saves energy, time, and resources, keeps flavor, texture and improves protein digestibility, hence the genotypes with minimum CT could be short listed for developing better lentil cultivars. Pearson correlation matrix revealed significant association among several traits, especially SY with BY, PS, and NSP that suggested their use for the future crop improvement program. The PCA revealed a considerable reduction in components for selection of suitable genotypes with desired traits that could be utilized for future lentil breeding. Structural Equational Model (<st1:stockticker w:st="on">SEM</st1:stockticker>) for SY based on covariance studies indicated the perfect relationship among variables. Further, hierarchical cluster analysis establishes four clusters for 2017-18, whereas seven clusters for 2018-19. Cluster 4 of 2017-18 and cluster 5 of 2018-19 exhibited the genotypes with best performance for SY, BY, PS, NSP, CT and HS. Based on heritability; HSW, SY, BY, NPS were found highly heritable. Traits with high heritability will be helpful for selecting genotypes high in variation which could exhibit genes of interest and efficient in improvement of the particular trait. In conclusion, 10 genotypes (5664, 5687, 6084, 6062, 6122, 6058, 6087, 5689, 6042, and 6074) have been identified for their use for lentil crop breeding programs.</p>

Order of Authors:
Muhammad Sajjad Iqbal, PhD
Syed Atiq Hussain
Muhammad Akbar
Noshia Arshad
Saba Munir
Muhammad Azhar Ali
Hajra Masood

Powered by Editorial Manager® and ProduXion Manager® from Aries Systems Corporation

	Tahira Ahmad
	Nazra Shaheen
	Ayesha Tahir, PhD
	Muhammad Kashif Ilyas, PhD
	Abdul Ghafoor, PhD
	Muhammad Ahson Khan, MSc Hons
**Response to Reviewers:**	Response to Reviewers comments<br>Authors pay thanks to the honorable Reviewers to improve this article a standard MS for wide readership. Further, MS has again reviewed thoroughly and edited sufficiently to make intangible and according to Standard English.<br>Below is the response to the Reviewers comments please;<br><br>Line 141: remove double comma and adjust spacing.<br>Double commas havs been removed and spacing adjusted accordingly, now at line 145 of the manuscript<br>P11, L 8-9: What do you mean by there in "Short duration genotypes are suggested as early maturing genotypes hence required further trials to see there early maturity cultivar status." Delete the entire sentence.<br>Deleted at line 197 of the final manuscript without track changes<br><br>P12, L5-7: Remove "Traits which possess low variability must be required to address through new collection by broadening existing lentil genetic resources through local as well as exotic sources." There are sentences at the end of each paragraph which are not making any sense. In the result section only point out to important findings. Discussion of results should be in the discussion section.<br><br>Authors have removed all of the unnecessary paragraphs as advised by the respectable reviewer, above mentioned lines have been removed as at line 220 of the manuscript<br>P13, L4-7: Under Pearson correlation matrix: authors indicated that HSW exhibited positive correlation with PS and BY. PH and PS also showed positive correlation with NSP<br>and DM but Table 4 only shows correlation of SY with other traits in both years. Either you have to show all pairwise correlation in table 4 or don't write about results that are not shown in the table.<br><br>Authors have removed the suggested characters and restricted to the traits as recommended.<br>P 21, L 16: Revise the sentence as "In this study, HSW varied between the years with 3.65 g in 2017-18 and 3.28 g in 2018-19"<br>Revised accordingly at line 386<br><br>P21, L 22: Cooking time not only saves energy and time but also it develops ….<br>Amended as suggested<br>P 23, L5: replace & with and<br>Replaced accordingly at line 420 and also in other paragraphs<br>P 23, L 9: Revise as "Similar results were observed"<br>Revised accordingly as suggested in the line 424<br>Conclusion: tons/hectare versus t/h be consistent throughout<br>Tons/hectare added as suggested in the line 477.
**Additional Information:**	
**Question**	**Response**
**Financial Disclosure**<br><br>Enter a financial disclosure statement that describes the sources of funding for the work included in this submission. Review	There is no financial assistance for publication of this research article

Powered by Editorial Manager® and ProduXion Manager® from Aries Systems Corporation

the submission guidelines for detailed requirements. View published research articles from *PLOS ONE* for specific examples.<br><br>This statement is required for submission and **will appear in the published article** if the submission is accepted. Please make sure it is accurate.<br><br>**Unfunded studies**<br>Enter: *The author(s) received no specific funding for this work.*<br><br>**Funded studies**<br>Enter a statement with the following details:<br>• Initials of the authors who received each award<br>• Grant numbers awarded to each author<br>• The full name of each funder<br>• URL of each funder website<br>• Did the sponsors or funders play any role in the study design, data collection and analysis, decision to publish, or preparation of the manuscript?<br>• **NO** - Include this sentence at the end of your statement: *The funders had no role in study design, data collection and analysis, decision to publish, or preparation of the manuscript.*<br>• **YES** - Specify the role(s) played.<br><br>* typeset	
**Competing Interests**<br><br>Use the instructions below to enter a competing interest statement for this submission. On behalf of all authors, disclose any competing interests that could be perceived to bias this work—acknowledging all financial support and any other relevant financial or non-financial competing interests.<br><br>This statement is required for submission and **will appear in the published article** if the submission is accepted. Please make sure it is accurate and that any funding sources listed in your Funding Information later in the submission form are also	The authors have declared that no competing interests exist.

declared in your Financial Disclosure statement.<br><br>View published research articles from *PLOS ONE* for specific examples.<br><br>**NO authors have competing interests**<br><br>Enter: *The authors have declared that no competing interests exist.*<br><br>**Authors with competing interests**<br><br>Enter competing interest details beginning with this statement:<br><br>*I have read the journal's policy and the authors of this manuscript have the following competing interests: [insert competing interests here]*<br><br>* typeset	
**Ethics Statement**<br><br>Enter an ethics statement for this submission. This statement is required if the study involved:<br><br>• Human participants<br>• Human specimens or tissue<br>• Vertebrate animals or cephalopods<br>• Vertebrate embryos or tissues<br>• Field research<br><br>Write "N/A" if the submission does not require an ethics statement.<br><br>General guidance is provided below. Consult the submission guidelines for detailed instructions. **Make sure that all information entered here is included in the Methods section of the manuscript.**	The study was approved by the Institutional Committee through No. UOG/ASRB/Botany/03/15806 accordingly all of the current experimental research and field studies are in compliance with relevant institutional, national and international guidelines and legislation.

Powered by Editorial Manager® and ProduXion Manager® from Aries Systems Corporation

**Format for specific study types**<br><br>**Human Subject Research (involving human participants and/or tissue)**<br>• Give the name of the institutional review board or ethics committee that approved the study<br>• Include the approval number and/or a statement indicating approval of this research<br>• Indicate the form of consent obtained (written/oral) or the reason that consent was not obtained (e.g. the data were analyzed anonymously)<br><br>**Animal Research (involving vertebrate animals, embryos or tissues)**<br>• Provide the name of the Institutional Animal Care and Use Committee (IACUC) or other relevant ethics board that reviewed the study protocol, and indicate whether they approved this research or granted a formal waiver of ethical approval<br>• Include an approval number if one was obtained<br>• If the study involved *non-human primates*, add *additional details* about animal welfare and steps taken to ameliorate suffering<br>• If anesthesia, euthanasia, or any kind of animal sacrifice is part of the study, include briefly which substances and/or methods were applied<br><br>**Field Research**<br><br>Include the following details if this study involves the collection of plant, animal, or other materials from a natural setting:<br>• Field permit number<br>• Name of the institution or relevant body that granted permission	
**Data Availability**<br><br>Authors are required to make all data underlying the findings described fully available, without restriction, and from the time of publication. PLOS allows rare exceptions to address legal and ethical concerns. See the PLOS Data Policy and FAQ for detailed information.	Yes - all data are fully available without restriction

A Data Availability Statement describing where the data can be found is required at submission. Your answers to this question constitute the Data Availability Statement and **will be published in the article**, if accepted.<br><br>**Important:** Stating 'data available on request from the author' is not sufficient. If your data are only available upon request, select 'No' for the first question and explain your exceptional situation in the text box.<br><br>Do the authors confirm that all data underlying the findings described in their manuscript are fully available without restriction?	
**Describe where the data may be found in full sentences. If you are copying our sample text, replace any instances of XXX with the appropriate details.**<br><br>• If the data are **held or will be held in a public repository**, include URLs, accession numbers or DOIs. If this information will only be available after acceptance, indicate this by ticking the box below. For example: *All XXX files are available from the XXX database (accession number(s) XXX, XXX.*).<br>• If the data are all contained **within the manuscript and/or Supporting Information files**, enter the following: *All relevant data are within the manuscript and its Supporting Information files.*<br>• If neither of these applies but you are able to provide **details of access elsewhere**, with or without limitations, please do so. For example:<br><br>*Data cannot be shared publicly because of [XXX]. Data are available from the XXX Institutional Data Access / Ethics Committee (contact via XXX) for researchers who meet the criteria for access to confidential data.*<br><br>*The data underlying the results presented in the study are available from (include the name of the third party*	All relevant data are within the manuscript and its Supporting Information files.

and contact information or URL).<br>• This text is appropriate if the data are owned by a third party and authors do not have permission to share the data.<br><br>* typeset	
Additional data availability information:	

Manuscript

Click here to access/download;Manuscript;Manuscript 27 Feb 2022.docx

Estimating genetic variability among diverse lentil collections through novel multivariate techniques

Syed Atiq Hussain[1], Muhammad Sajjad Iqbal[1]*, Muhammad Akbar[1], Noshia Arshad[1], Saba Munir[1], Muhammad Azhar Ali[1], Hajra Masood[1], Tahira Ahmad[1], Nazra Shaheen[1], Ayesha Tahir[2], Muhammad Ahson Khan[3], Muhammad Kashif Ilyas[3], and Abdul Ghafoor[3]

[1]Biodiversity Informatics, Genomics and Post Harvest Biology Laboratory, Department of Botany, University of Gujrat, Gujrat, 50700-Pakistan;

[2]Departmet of Biosciences, COMSATS University, Chak Shahzad Campus, Park Road, Islamabad;

[3]National Agricultural Research Center (NARC), Islamabad, Pakistan

*****Correspondence: drsajjad.iqbal@uog.edu.pk (MSI)

These authors contributed equally to this work.

Acknowledgement

We are grateful to Bio-Resource Conservation Institute (BCI), NARC, Islamabad, Pakistan for providing Lentil germplasm and experimental field facilities.

Abstract

Lentil is an important food legume throughout the world and Pakistan stands at 18th position with 8,610 tons production from 17,457 hectares. It is rich in protein, carbohydrates, fat, fiber and minerals that can potentially meet food security and resolve malnutrition issues, particularly in South Asia. Two hundred and twenty lentil genotypes representing Pakistan (178), Syria (14), and USA (22) including 6 from unknown origin were studied for yield, yield contributing traits, and cooking time (CT). The genotype 6122 (Pakistan) performed the best during both years with seed yield per plant (SY) 68±1.7 g, biological yield per plant (BY) 264±2.8 g, pod size (PS) 0.61±0.01 cm, number of seeds per pod (SP) 2, cooking time (CT) 11 minutes, with no hard seed (HS). The genotype 6122 (Pakistan) and 6042 (Syria) produced the highest BY, hence these have potential as an efficient source for fodder, particularly during extreme winter months. The genotype 5698 (Pakistan) and 6015 (USA) were late in maturity, during 2018-19 while 24783 and 5561 matured early in 2019. Minimum CT 10 minutes was taken by the genotypes 6074 and 5745 of Pakistani origin. The lowest CT saves energy, time and resources, keeps flavor, texture and improves protein digestibility, hence the genotypes with minimum CT could be short listed for developing better lentil cultivars. Pearson correlation matrix revealed significant association among several traits, especially SY with BY, PS and NSP that suggests their use for future crop improvement program. The PCA revealed a considerable reduction in components for selection of suitable genotypes with desired traits that could be utilized for future lentil breeding. Structural Equational Model (SEM) for SY based on covariance studies indicated the perfect relationship among variables. Further, hierarchical cluster analysis establishes four clusters for 2017-18, whereas seven clusters for 2018-19. Cluster 4 of 2017-18 and cluster 5 of 2018-19 exhibited the genotypes with best performance for SY, BY, PS, NSP, CT and HS. Based on

heritability; HSW, SY, BY, NPS were found highly heritable. Traits with high heritability will be helpful for selecting genotypes which exhibit genes of interest and efficient in improvement of the particular trait. In conclusion, 10 genotypes (5664, 5687, 6084, 6062, 6122, 6058, 6087, 5689, 6042 and 6074) have been identified hence suggested for future lentil breeding programs.

Introduction

Lentil (*Lens culinaris* Medik.) is an annual, herbaceous, cool-season, autogamous (self-pollinated) and diploid legume with 4 Gbp genome size that was domesticated with wheat and barley in the Fertile Crescent [1, 2]. It is one of the oldest dry legumes domesticated about 9,000 years ago in the Near East and subsequently spread throughout the Mediterranean Basin and Central Asia [3]. Lentil is the 4th major pulse crop in the world after common bean, pea and chickpea, and included seven taxa with four species such as *L. culinaris*, *L. nigricans*, *L. ervoides* and *L. lamottei*, the only cultivated species (*L. culinaris*) have two varietal types as macro-sperma (large seeded) and micro-sperma (small seeded) [4, 5].

Lentil is consumed due to its highest protein contents, vitamins and micronutrients that are a vital part of human nutrition while vegetative parts are being used as fodder for livestock [6]. Protein in lentil seed is of high quality which is 22-35% of total seed mass and consisted of a large number of amino acids including lysine and isoleucine, hence it is a cheap source of protein in many regions of the world both for humans as well as for animals [7].

Additionally, lentil is a rich source of carbohydrates (59%), minerals (2%) and fiber with very little fat which is significant to overcome malnutrition and deficiency of micronutrients in

developing countries [8]. It is also the best source of iron to prevent iron deficiency [9]. Medicinally it has low glycemic content, due to this physicians strongly suggest for patients with diabetes, obesity and heart maladies [9]. Seeds of lentil are mucilaginous and laxative, recommended for treating constipation, stomach infections, smallpox and ulcer [6]. The top ten lentil producing countries are Canada, India, Turkey, USA, Kazakhstan, Nepal, Australia, Russia, Bangladesh and China [10].

The imperative causes of the low yield of lentil in Pakistan are cultivation on marginal lands with poor management practices, lack of high yielding varieties, biotic and abiotic stresses, and above all adaptation of low yielding genotypes in different environments with minimum genetic variability with critically low productivity [11, 12]. It is economically not feasible to reduce biotic stresses by utilizing pesticides, herbicides and to reduce abiotic stresses by supplementing soil to meet plant demand through reclamation and enhancing fertilizer, as well as by improving irrigation systems [13]. Alternatively, the best solution to overcome these problems is to develop superior planting material. Regarding this, breeding and modern bio-techniques could improve crops by transferring a gene of interest or genetic modification. The inadequate information of genetic variability in existing lentil is a major hindrance to harvesting the potential including inadequate use of modern molecular techniques for genetic improvement [13].

Several studies have been carried out worldwide to assess genetic diversity based on phenotypic markers in lentil as well other legumes in the past [3, 7, 11, 14-19]. Interestingly, germplasm repository is a valuable asset that carries a huge treasure of genetic material for future crop improvement and development programs [20]. As genetic diversity can be estimated with the help of qualitative and quantitative traits, likewise, a large number of morphometric

techniques have been used to measure and classify genotypes in mustard, peas, mungbean, alfalfa, cowpea, soybean and lentil [21-26]. Current studies engaged several statistical techniques and aiming to unwind the hidden potential of genetic diversity in the available lentil genetic resources and to assess variability in agro-morphological traits for consecutively two years. Identified novel genotypes would be the base material for future lentil breeding programs.

Material and Methods

Ethics statement on experimental research

The study was approved by the Institutional Committee through No. UOG/ASRB/Botany/03/15806. Accordingly, all of the current experimental research and field studies comply with relevant institutional, national and international guidelines and legislation.

Plant material, study site, research design, and crop cultivation

Two hundred and twenty lentil genotypes representing Pakistan, the USA and Syria including unknown origin were obtained from National Gene bank, National Agricultural Research Center (NARC), Islamabad, Pakistan (S1 Table). Germplasm planted under field conditions of NARC in Islamabad, Pakistan located at 33.6701° N latitude and 73.1261° E longitude during October 2017 and harvested in April 2018, similarly, same timing and procedure were followed in the second year 2018-19. Both qualitative and quantitative traits were recorded consecutively for two years (2017-18 and 2018-19). While meteorological data

recorded on monthly basis was obtained from the experimental station (S1 Fig.). Experiments were laid out in an augmented design within 11 blocks and each block consisted of 20 genotypes along two check varieties, *viz.*, Markaz 2009 and Punjab 2009 after every twenty lines [16]. Three rows of 5 m length in each bed were planted with 10 cm space between plants and 30 cm within rows. The trial was conducted under usual agricultural practices without applying any herbicide, fungicide, pesticide or fertilizer so that the actual genetic diversity may be studied. Soil was homogenously fertile and analyzed in Soil Science Laboratory, NARC, Islamabad, which was Nabipur series, coarse loamy, mixed, hyperthemic, and udic ustochrepts. Recommended cultural practices were followed throughout the crop season [27].

Data collection

Ten healthy plants were randomly sampled from each genotype on a plant basis for recording of the data. Quantitative traits were recorded at foliage, reproductive and maturity stages including plant height (PH), number of pods per peduncle (NSP), lower pod height (LPH), pod size (PS), number of seeds per pod (NSP), and days to maturity (DM). Days to maturity were considered from the day of sowing to turning of 90% pods into golden brown color. After harvesting, biological yield (BY), seed yield (SY), 100 seed weight (HSW), cooking time (CT) and hard seeds (HS) were recorded after boiling 100 seeds. Cooking time was calculated in minutes for cooking un-soaked seeds to softness in boiling distil water.

For qualitative traits, observations were recorded for ground color of testa (GCT), cotyledon color (CC), color of pattern on testa (CPT), pod dehiscence (PD), pod shedding (PSh), leaflet size (LS), tendril length (TL), ground color of flower (GCF), pod pigmentation (PP), leaf pubescence (LP) and seedling stem pigmentation (SSP). At late vegetative stage, SSP designated as '0' for absent and '1' present, LS '3' for small, '5' medium and '7' large and LP '0' absent,

‘3’ slight and ‘7’ dense. At early reproductive stage, GCF ‘1’ white color, ‘2’ white with blue lines, ‘3’ blue, ‘4’ violet, ‘5’ pink and ‘6’ for others, were observed. At late reproductive stage, PP ‘0’ for absent and ‘1' present were recorded. At the stage of maturity, PD was categorized and recorded as ‘0’ none, ‘3’ low, ‘5’ medium and ‘7’ high and PSh ‘0’ none, ‘3’ low, ‘5’ medium and ‘7’ high. Tendril length included ‘1’ rudimentary tendril and ‘2’ for prominent tendril at late reproductive stage. After harvesting, CPT ‘0’ absent, ‘1’ olive, ‘2’ grey, ‘3’ brown, ‘4’ black.), CC ‘1’ yellow color, ‘2’ orange-red and ‘3’ olive green) while GCT represented ‘1’ green, ‘2’ grey, ‘3’ brown, ‘4’ black and ‘5’ pink.

Statistical analysis

Descriptive statistics of quantitative traits including mean, standard deviation, standard error of mean, range with minimum and maximum values, coefficient of variance, Pearson correlation and PCA were performed through statistical software XLStat 2019. Hierarchical cluster analysis conducted to construct dendrograms based on Euclidean distances using RStudio. The structural equation model (SEM) was drawn through LISREL (LISREL Student Version 9.2 https://lisrel-for-windows-student.software.informer.com/9.2/) to estimate multiple regressions that ~~indicating~~ the contribution of quantitative traits toward yield. Moreover, the genotypic, phenotypic, environmental variances and ~~broad sense~~ heritability were estimated by the formulas as provided below while genetic advance and genetic advance in percentage of mean were derived through variability package in R Studio [28, 29].

Genotypic variance

$$GV = \frac{GMS - EMS}{r}$$

Phenotypic variance

$$PV = \frac{GV - EV}{r}$$

Broad sense heritability

$$h^2 = \frac{GV}{PV}$$

Where GV and PV symbolized as the genotypic and phenotypic variances, respectively; GMS, genetic mean square; EMS, error mean square; r, number of replications; EV, environmental variances and h^2 represented as broad sense heritability.

Result

Genetic variation based on quantitative traits

High genetic variability was observed among lentil germplasm which could broadened the scope of simple selection and opportunity for new recombinants for genetic improvement. Genotype 6122 collected from Sheikhupura, Pakistan performed outstanding for SY (68±1.7 g), BY (264±2.8 g), PS (0.61±0.01cm), NSP (2), CT (11 minutes) with no HS. Mean values of SY (30.08±0.98 g) were higher in 2017-18 as compared to SY (25.26 ±0.87 g) in 2018-19 (Table 1). In comparison, check varieties showed low SY in 2018-19 as compared to 2017-18. It ranged from 5 to 85 g in 2017-18 and 3 to 76 g during 2018-19, respectively. It was noted that genotype 6084 belonged to Narowal, Pakistan produced the highest SY in both years while the lowest SY was recorded in genotype 5475 originated from Gujranwala, Pakistan for 2017-18. Likewise, genotype 5494 from Okara, Pakistan and 5475 from Gujranwala, Pakistan were the lowest in SY during 2018-19. Genotypes 6052 (Syria), 5583 (Muzaffargarh, Pakistan), and 5556 (Hyderabad,

Pakistan) produced better SY for both years. Overall performance of genotypes was highest during first year as compared to the subsequent year which could be attributed due to environmental conditions during second year. Evaluation based on both years with varying environmental conditions provide assistance in selection for not only common genotypes but also suggested varying environmental or location trials in future.

Biological yield is a significant contribution towards SY in lentil and the genotypes 6122 (Sheikhupura, Pakistan), 5689 (Sialkot, Pakistan), 5730 (Layyah, Pakistan), and 6037 (Syria) produced the highest BY 264±2.8 g, 258±1.0 g, 254±1.5 g, and 254±2.0 g, respectively in 2017-18, whereas genotypes 6042 (Syria), 6087 (Narowal, Pakistan) and 5689 (Sialkot, Pakistan) were the best among others for BY i.e., 187.0±1.3 g, 183.0±0.9 g, and 179±1.3 g, in 2018-19. Whereas plant height (PH) during 2018-19 was slightly lower than 2017-18, PH has significant contribution towards acquiring light and high number of branches to produce more seeds.

Table 1. Descriptive statistics for quantitative traits studied in lentil during 2017-18 and 2018-19**.**

	2018-19							2017-18					
**Traits**	**P values**	**Mean±SE**	**Min.**	**Max**	**SD**	**Markaz 2009**	**Punjab 2009**	**Mean±SE**	**Min.**	**Max.**	**SD**	**Markaz 2009**	**Punjab 2009**
SY	0.0001 s	30.08±0.9	5	85	14.52	41.72±1.0	55.27±2.4	25.26±0.8	3	76	12.91	39.08±0.9	53.27±1.7
HSW	0.254 ns	1.71±0.02	1.05	3.65	0.35	1.91±0.01	2.48±0.01	1.68±0.01	1.17	3.28	0.28	1.85±0.01	2.4±0.1
BY	0.0001 s	114.6±3.0	42	264	44.4	87.5±1.8	112.2±6.39	94.13±2.2	34	187	32.77	88.58±1.6	110.4±5.1
PH	0.0001 s	44.73±0.4	27.38	71.12	6.86	59.09±1.4	55.1±1.4	46.7±0.4	29.6	69	6.94	61.58±1.1	56.54±0.9
LPH	0.958 ns	14.97±0.1	9.14	32.16	2.34	15.32±0.4	15.6±0.36	14.98±0.1	10.72	33.34	2.03	16.56±0.6	16.71±0.6
PS	0.0001 s	0.3±0.01	0.2	0.7	0.08	1.55±0.20	1.36±0.01	0.3±0.01	0.2	0.7	0.08	1.33±0.01	1.37±0.01
NSP	0.0001 s	2.00±0.01	1	3	0.16	2±0.01	2±0.01	2.00±0.01	1	3	0.28	2±0.01	2±0.01
DM	0.0001 s	184±0.3	161	198	5.05	172±0.29	170±0.29	186±0.34	161	198	5.05	172.5±0.2	169±0.3
CT	0.0001 s	14.55±0.1	10	18	1.52	12.09±0.1	11.7±0.19	16.15±0.1	10	18	1.52	12.16±0.1	11.90±1.6
HS	0.0001 s	0.37±0.01	0	2	0.61	0.27±0.1	0.25±0.17	0.41±0.04	0	2	0.61	0.41±0.1	0.09±0.9
NP	0.0001 s	2.04±0.01	2	3	0.19	3±0.01	3±0.01	2.00±0.01	1	3	0.16	3±0.01	3±0.01

SY, seed yield; HSW, hundred seed weight; BY, biological yield; PH, plant height; LPH, lower pod height; PS, pod size; NSP, number of seed per pod; DM, days to maturity; CT, cooking time; HS, hard seed/hundred seed; NP, number of pods per peduncle; SE, standard error of mean; SD, standard deviation; p-value <0.05.

Days to maturity ranged from 161 to 198 days in both years. However, mean values were 184±0.34 days during 2017-18 and 186±0.34 days during 2018-19. Long duration genotypes were 5684 (Narowal, Pakistan), 5988 (USA), 5979 (USA), 5679 (Narowal, Pakistan), 5982 (Narowal, Pakistan), 5683 (Narowal, Pakistan), 24786 (unknown), 5623 (Faisalabad), 6077 (Bahawalnagar, Pakistan) and 5684 (Narowal, Pakistan) need further consideration to reduce the time for maturity, but these would be available for meeting fodder requirement for longer time, Following short duration genotypes (5698, 6015, 5667, 5748, 5555, 6017, 5700, 5571, 5580 and 5981 matured in < 178 days.

Both check varieties were insignificant within the same year, however overall performance was better during 2017-18 as compared to 2018-19. It indicates homogeneity of the experimental site and the validity of augmented design due to higher genetic variance. Superior genotypes based on the best performance for individual traits have been identified and presented in Table 2, which are recommended for future crop improvement programs. More than 40 g SY was produced by three genotypes 6084, 6062, and 6122 during both years, hence selected for utilization of yield potential. Cooking time is very crucial particularly in the era of energy crises and higher fuel prices, hence short CT is an emerging selection criterion for most of the legumes including lentil. Keeping in view this important issue, seven genotypes *viz.,* 6074, 5595, 6075, 2018, 6092, 6099 and 5511 which took <12 minutes CT were short listed. A list of genotypes representing the best 5% of the germplasm including check varieties is presented (S2 Table and S3 Table).

Table 2. List of best performing lentil genotypes selected during both years for quantitative traits.

Traits	During 2018	During 2019
SY > 40g	6084, 6062, 6122, 6058, 6087, 5689, 6042, 6074, 5664, 5687	6084, 6062, 6122, 6058, 6087, 5689, 6074, 6042, 5664,5687,
HSW > 1.90g	6052, 5583, 5556, 6101, 5684, 23776,	5583, 6052, 5684, 6101, 5556, 5643, 23776

	5643	
BY > 100g	6122, 5689, 6037, 6042, 5600, 5982, 5658, 6084	6122, 5689, 6037, 6042, 5600, 5982, 5658, 6084
PH > 55	5549, 5687, 5723, 5549, 5687	5549, 5687, 5723, 5549, 5687
LPH > 15cm	5527, 6062, 5751, 5686, 5744, 5477	5527, 6062, 5751, 5686, 5744, 5477
PS > 0.4 cm	5664, 6124, 6116,6077, 6082	5664, 6124, 6116,6077, 6082
NSP > 2	6122, 5687, 24784, 6012, 6084	6122, 5687, 24784, 6012, 6084
DM < 180 days	24783,5741, 5729, 6081, 5981	24783, 5741, 5729, 6081, 5981
CT < 12 minutes	6074,6075,6010, 6013, 6041	6074,6075,6010, 6013, 6041
HS < 2	6074, 5595, 6075, 6010,6041	6074, 5595, 6075, 6010,6041
NP > 2	5595,5861, 5691, 5671,5689	5595,5861, 5691, 5671,5689,

SY, seed yield; HSW, hundred seed weight; BY, biological yield; PH, plant height; LPH, lower pod height; PS, pod size; NSP, number of seed per pod; DM, days to maturity; CT, cooking time; HS, hard seed/hundred seed; NP, number of pods per peduncle

Genetic variation in qualitative traits

High variation was observed in qualitative traits as various lentil plant descriptors were described by dividing into categories (Table 3). The ground color of testa (GCT) exhibited four categories, out of which more than half of lentil germplasm have grey color, 33.6% as brown, 13.2% black while few genotypes (1.4%) were green. Cotyledon color (CC) was predominantly red, consumers' prefer red cotyledon color which have more market demand.

Table 3. Frequency distribution of qualitative traits of lentil germplasm studied during 2017-18 and 2018-19

Variable	No. of Categories	Categories Name	Frequency (%)	Variable	No. of Categories	Categories Name	Frequency (%)
Ground color of testa (GCT)	4	Green	1.36	Pod shedding (PS)	3	Low	55.9
		Grey	51.82			Medium	26.8
		Brown	33.64			High	17.3
		Black	13.18	Leaflet size (LS)	3	Small	62.73
Cotyledon color (CC)	3	Yellow	2.27			Medium	28.18
		Red	96.36			Large	9.09
		Olive green	1.36	Pod pigmentation (PP)	2	Absent	69.09
Color of pattern on testa	5	Absent	0.91			Present	30.91

(CPT)							
		Olive	7.73	Leaf pubescence (LP)	2	Slight	85.00
		Grey	59.54			Dense	15.00
		Brown	13.64	Seedling stem pigmentation.(SSP)	2	Absent	69.09
		Black	18.18			Present	30.91
Pod dehiscence (PD)	4	None	0.91				
		low	87.7				
		Medium	8.21				
		High	3.20				

Pearson correlation matrix

The correlation matrix for quantitative traits presented in Table 4 revealed positive association of SY with BY, PS, NSP, and NP. Significant association among various traits showed effectiveness and contribution of yield and yield contributing traits in a symmetry that can be translated to boost yield and for selection of genotypes with desirable traits. In the same context, lentil genotypes grown in 2018-19 indicated that SY exhibited a significant positive correlation with HSW, BY and PH, rests assure that these traits contributed directly towards seed yield. ~~HSW was positively correlated with BY, in return BY was also positively correlated with PH. Desirable combinations described high potential of the germplasm that can be utilized in future crop improvement programs through selection.~~

Table 4. Pearson Correlation matrix for SY with other quantitative traits of lentil grown in 2017-18 and 2018-19

**Traits**	**Years**	**HSW**	**BY**	**PH**	**LPH**	**PS**	**NSP**	**DM**	**HS**	**NP**
**SY**	2018	0.09	0.73*	-0.09	0.03	0.32*	0.15*	0.15*	0.11	0.18*
	2019	0.27*	0.83*	0.21*	-0.16*	-0.11	-0.11	-0.05	-0.01	0.08

SY, seed yield; HSW, hundred seed weight; BY, biological yield; PH, plant height; LPH, lower pod height; PS, pod size; NSP, number of seed per pod; DM, days to maturity; HS, hard seed; NP, number of pods per peduncle

Principal component analysis (PCA)

The PCA for 2018 indicated that first five components have > 1.0 eigenvalue as 2.09, 1.37, 1.19, 1.08 and 1.02, respectively (S4 Table). SY, BY and PS contributed more toward component 1. Similarly, component 2 can be selected for HSW, PH and HS. PCA advocates further selection of genotypes with specific trait/s without losing total variation and mush better idea about evaluated genotypes. For second year selection can be made for factor 1 including SY (0.84), BY (0.84) and NP (0.02) which contributed highest factor loading values while factor 2 can be selected for HSW (0.32), PS (0.63) and DM (0.15) as shown in Fig. 1.

Fig. 1. PCA showing the contribution of quantitative traits among 220 lentil genotypes grown in 2017-18.

Further, scattered diagram based on first two factors presented in Fig. 2 showed that the genotypes *viz*., 5638, 5583, 6124, 6052, 6010, 5684, 5979 and 6101 were present in the extreme upper right side of the scattered plot due to response towards HSW, DM and PS. The genotypes 6054, 5556, 23776 and 5650 were placed at an extreme upper left side with mixed performance of the genotypes for various traits. Such type of genotypes required further evaluation and characterization to explore full potential of the germplasm. Similarly, the genotypes 6122, 6084, 5689 and 5590 presented on the lower right side of the scattered plot were contributed through BY, SY, NP, NSP and HS.

Fig. 2. Scattered plot of PCA for lentil genotypes studied during 2017-18.

Moreover, based on PCA during 2019, first five components had > 1 Eigenvalue, whereas other PCs contributed low in cumulative variation (S5 Table). Contribution by SY, BY,

and HSW were highest toward factor 1 while factor 2 was contributed more by CT, SP and HS. The separation of genotypes and their accumulation in the particular principal components indicated the performance and association towards yield. Resultantly, factor 1 showed 19.91% variability while factor 2 as 12.19 % with an overall contribution of 32.10 % (Fig. 3).

Fig. 3. PCA showing the contribution of quantitative traits among 220 lentil genotypes grown in 2018-19.

Furthermore, genotypes 5506, 5729, 5510, 5672, 5636, 5581, 5491 and 5483 at the upper right corner were grouped on the basis of HSW, BY and SY indicating elite genotypes while in the lower right corner genotypes 5472, 5512, 5479, 5486, 5650, 5600 and 5531 reflected promising results for PH, NP and HS. Therefore, increase in height would result in higher number of seeds per pods which ultimately would contribute towards high yield. Similarly, at the upper left side of the scattered plot, genotypes 6080, 6043, 5653, 6043, 6002, 6060 and 5677 showed variability and high response to CT, NSP and PS which could be helpful in selection for genotypes with desirable CT. ~~While on the other hand, at lower left side~~ genotypes 23787, 24787, 23779, 5742 and 5712 were placed with the highest variability for DM and LPH which supports to select such genotypes that showed a positive association between both of these traits (Fig. 4). Likewise, lower pod height helps in early or timely maturity of the crop, as stature of plant helps in gathering more light and less shade, probably high metabolism and access to resources.

Fig. 4. Scattered plot of PCA for lentil genotypes grown in 2018-19

Hierarchical cluster analysis

Cluster analysis based on quantitative data in 2017-18 is presented in Fig. 5 which had divided the genotypes into 4 main clusters based on 50% linkage distance (LD) of 14.5. Cluster 1 consisted of 106 diverse genotypes including 6015 originated from USA followed by cluster 2 which contains 85 genotypes including 5698 originated from Sialkot, Pakistan with distinct position. Cluster 3 consisted of 20 genotypes where 3 are representing Pakistan found distinct (5689 Sialkot, 5687 and 6084 from Narowal). Cluster 4 composed of only 9 genotypes with unique performance for SY, BY, PS, NSP, CT and HS. Selection could be made for clusters having unique performance with desired traits, hence suggested to use for future lentil breeding.

The hierarchical cluster pattern obtained for 2nd year 2018-19 indicated 7 main clusters at 50% linkage distance of 8.4 (Fig. 6). Cluster 1 consisted of 37 genotypes, cluster 2 (115), cluster 3 (12), while cluster 4 and 5 were of 1 (24783) and 4 genotypes (6084, 6058, 6062 and 6122), respectively. Further, cluster 6 consisted of 49 genotypes and cluster 7 only 2 genotypes. Best performing genotypes were represented in C3 during 2017-18 and C5 during 2018-19 due to their performance as SY, BY, PS, NSP, CT and HS. Cluster 2 represents HSW > 1.19 producing genotypes during 2017-18, on the other hand, those genotypes which produced HSW > 1.19 during 2018-19 were grouped into cluster 3. Similarly, cluster 2 was also consisted of those genotypes which had PH > 55.1 and DM > 170 in both years. Further, cluster 1 consisted of genotypes having SY > 40 or near to 40 g during both years.

Fig. 5. Hierarchical dendrogram showing the relationship of lentil genotypes for quantitative traits studied during 2017-18.

Fig. 6. Hierarchical dendrogram showing the relationship of lentil genotypes for quantitative traits studied during 2018-19.

Structural equational model (SEM)

SEM was constructed for SY based on covariance studies as shown in Table 5. SY was taken as a dependent variable whereas other traits such as HSW, BY, PH, LPH, PS, DM, NP and NSP were taken as independent variables (Fig. 7). Contribution of independent variables like HSW (P values 0.10), BY (P values 0.09), PH (P values 0.00), LPH (P values 0.01), NSP (P values 0.81), PS (P values 0.00), DM (P values 0.00) and NP (P values 0.04) ~~observed~~ significant at level < 1. This was further verified by the lowest error variance of multiple linear regressions. The value of coefficient of determination (R^2 = 0.64) of these multiple linear relationships supports this hypothesis that real yield contributors are given in the equation and these individual traits are contributing in their percent capacity share as shown in ~~figure.~~

Fig. 7. Structural equations of model for seed yield (SY) during 2017-18 and 2018-19

Table 5. Structural equational model representing regression for agronomic traits studied during 2017-18 and 2018-19.

SY	HSW	BY	PH	LPH	NSP	PS	DM	NP
Z-values	2.12	0.27	0.02	0.43	3.69	5.43	-0.04	2.21
P-values	0.10	0.09	0.00	0.01	0.81	0.0	0.0	0.04

SY = 2.16*HSW + 0.27*BY + 0.025*PH + 0.0.43*LPH + 1.826*NSP + 6.34*PS - 0.039*DM + 2.298*NP

SY, seed yield; BY, biological yield; PH, plant height; LPH, lower pod height; PS, pod size; DM, days to maturity; NP, number of pods per peduncle; NSP, number of seeds per pod.

Heritability analysis

Phenotypic coefficient of variance ~~founded to be~~ greater than the genotypic coefficient of variance during 2017-18 and 2018-19 for all ~~the traits under study~~ (Table 6). High heritability was observed in 220 lentil genotypes along with ~~two~~ check verities. Seeds per pod showed low heritability value 0.46 during 2017-18 while considerably high during 2018-19 as 0.88. Other

traits showed high heritability such as 0.99 for BY, PH, LPH and DM during 2017-18 while during 2018-19 LPH and DM showed the same heritability value. The high heritability was observed for BY (0.99) along with high genetic advance as a percentage of mean 79.71 for 2017-18 while for the subsequent year LPH was 27.94. It indicates predominance of additive gene action for these traits under prevailing conditions. Further, high genetic advance along high heritability estimates provides ample opportunity for most suitable selection of traits for future application through crossing or hybridization for varietal development.

Table 6. Estimation of heritability and genetic advance for the years 2017-18 and 2018-19

Traits/Years	Environmental Variance		Genotypic Variance		Phenotypic Variance (σ_p)		Environmental Coefficient of Variance		Genotypic Coefficient of Variance		Phenotypic Coefficient of Variance		Heritability (Broad Sense) (h^2)		Genetic Advance		Genetic Advance as percentage of mean	
	2018	2019	2018	2019	2018	2019	2018	2019	2018	2019	2018	2019	2018	2019	2018	2019	2018	2019
SY	6.21	3.75	206	163.9	212.9	167.72	8.23	7.69	47.47	50.84	48.18	51.41	0.97	0.97	29.18	26.08	96.36	103.55
HSW	0.01	0.00	0.11	0.07	0.12	0.08	7.64	5.66	19.3	15.83	20.80	16.81	0.86	0.88	0.63	0.51	37.06	30.71
BY	16.9	33.09	1978	1050.9	1995	1084.	3.59	6.11	38.85	34.43	39.02	34.97	0.99	0.96	91.24	65.75	79.71	69.85
PH	0.46	0.61	47.04	48.10	47.5	48.72	1.51	1.61	15.26	14.23	15.34	14.32	0.99	0.98	14.06	14.19	31.29	29.13
LPH	0.02	0.02	5.52	4.16	5.54	4.18	1.01	1.10	15.87	13.61	15.90	13.65	0.99	0.99	4.83	4.18	32.63	27.94
PS	0.00	0.00	0.01	0.01	0.01	0.01	3.07	4.88	20.4	21.92	20.70	21.92	0.97	0.95	0.17	0.16	41.48	44.24
SP	0.01	0.00	0.01	0.07	0.02	0.08	5.69	3.21	5.34	13.66	7.81	14.04	0.46	0.94	0.15	0.56	7.52	27.40
DM	0.13	0.13	25.60	25.60	25.73	25.74	0.19	0.19	2.73	2.73	2.74	2.74	0.99	0.99	10.39	10.39	5.62	5.62
HS	0.00	0.01	0.37	0.36	0.38	0.38	25.88	30.52	163.9	163.12	165.9	165.9	0.97	0.96	1.24	1.23	333.5	330.32
NP	0.01	0.00	0.04	0.02	0.05	0.03	5.50	2.51	9.69	7.90	11.15	8.29	0.75	0.90	0.35	0.31	17.36	15.53

SY, seed yield; HSW, hundred seed weight; BY, biological yield; PH, plant height; LPH, lower pod height; PS, pod size; NSP, number of seeds per pod; DM, days to maturity; CT, cooking time; HS, hard seed; NP, number of pods per peduncle.

Discussion

Selection of ~~the~~ promising genotypes is the ultimate objective of ~~scientists~~ to develop cultivars for farmers' field. Genetic diversity has been used to characterize the genotypes in a range of plant species including lentil [30]. Wide variation was observed in the present study in lentil including SY from 3 to 85 g by 6084, which produced highest SY for both years [16]. ~~It is always to be desirable by breeders to choose high yielding genotypes exhibit highest yield potential to develop varieties or to carry out multi-location trials for varying environments [31].~~ High BY increases the photosynthetic area which in turn enhances food quality and SY. In the present study, 6122, 5689, 5730 and 6037 exhibited the best BY during 2017-18, likewise genotypes 6042, 6087 and 5689 were best for 2018-19. Improvement in BY at the vegetative growth stage before flowering leads to further increase in N-assimilation and thereby in SY [24, 33-35]. Nitrogen assimilation transfer atmospheric nitrogen which is available in the environment into amino acids, therefore it is one of the natural property of legumes which make them more proteinaceous food.

~~Overall performance~~ was better in the first year as compared to subsequent year that is more likely ~~to be~~ attributed to temperature fluctuation during both of the years, from 21°C in March to 26°C in April during 2017-18 and 19°C in March to 24°C in April during 2018-19. It is presumed that low temperature delayed flowering and pod filling process, however lentil requires low temperature during vegetative stage, while the reproductive stage needs high temperatures [36]. The optimum temperature for vegetative stage of lentil ranged from 6.1-27.3˚C while 17.4-31.5˚C during reproductive stage [37]. Likewise, plant height (PH) is a major yield contributing trait which supports sufficient vegetative growth and branching, ultimately ~~develops~~ more pods that means increased SY [7, 38].

High variation in native as well as exotic germplasm was found for both years. ~~There were additionally several environmental factors which influence crop performance as water scarcity affects productivity of legumes at growth stage,~~ perhaps ~~drought~~ during reproductive and grain filling stages (terminal drought) ~~are~~ more critical and usually ~~result~~ in a significant loss of SY and of course negative effects on yield contributing traits too [39, 40]. ~~In the present studies~~, genotypes 5698 (Sialkot, Pakistan) and 6015 (USA) were ~~found as~~ early maturing ~~genotypes~~ in 2017-18 while 24783 (unknown) and 5561 (Sanghar, Pakistan) for 2018-19 with 161-172 days. Current findings are more appealing as compared to previous reports of 168-190 days [16]. These early maturing genotypes are suggested for ~~further~~ crop improvement ~~program as well against~~ drought hit areas ~~during cold season after adopting breeding strategy for lentils [41]~~.

It is evident that SY is directly proportional to NSP, NP ranged from 2-3 in 2017-18 and 1-3 in 2018-19, selection based on stable genotypes for NP would proof baseline material for starting multi-location trials under different environmental conditions to develop new varieties [17]. Similarly, HSW is considered as an indicator of seed quality and a measure of grain bulk density. In this study HSW varied between the years with 3.65 g in 2017-18 and 3.28 g in 2018-19~~, showed wide variation during both years~~. Similar ~~type of~~ studies were also conducted for wide range of crops including lentil not only in Pakistan and Turkey but also in other countries of the world with respect to agro-morphological characterization [3, 7, 11, 14-18, 39, 42].

~~Further~~, cooking time (CT) is a favorite trait for consumers in case of legume crops~~, it saves energy~~. CT of 10 minutes was observed in genotypes 6074 and ~~5745~~. It ~~is~~ not only saves energy and time but also it develops adequate flavor, texture and improves protein digestibility in lentils [8]. More CT causes loss of proteins, vitamins, minerals and other secondary metabolites

[9]. ~~Overall, the~~ genotype 6122 performed the best out of 220 genotypes, therefore, suggested ~~further for trials and breeding programs~~ to develop ~~as a~~ new variety for commercial purposes.

Inheritance studies of qualitative traits like GCT and GPT ~~evidenced~~ that GCT is determined by two independent loci [43]. The dominant allele at one locus (Ggc) determines grey seed coat color and dominant allele at second locus (Tgc) determines tan seed coat color. Two dominant alleles (Ggc Tgc) interact to produce a brown ~~seed~~ GCT whereas double recessive (ggc tgc) as green color. Seed coat pattern is determined by a series of five alleles at another locus [41]. Moreover, pod pigmentation was absent in 69% of genotypes with low PD and PSh. Low PD and PSh are considered most favorite traits from a farmer's perspective because ~~it reduces~~ grain loss during harvesting. Further, slight LP with a small leaf and prominent tendril were most frequently found. While dense LP was found in 15% of genotypes. Presence of dense LP ~~(leaf hairs)~~ reduces absorbance of photosynthetically active radiation (400 to 700 nm). Reduction in photosynthetic rate is caused by decreased light absorption rather than decreased carbon dioxide conductance through boundary layer [17]. It has been also reported that light LP with a small leaf and prominent tendril exhibit high resistance to lodging. Additionally, seedling stem pigmentation was present in most varieties. All of the genotypes exhibited white blue line flowers [42].

Improvement in a target character can be achieved by indirect selection which are more heritable and easier to select [43]. ~~Further~~, this strategy of selection requires ~~understanding~~ interrelationships among characters and with target traits. A significant correlation between SY and most of the quantitative traits were documented in both years. Positive combinations can be helpful in future hybridization programs to obtain highest outcomes. It is recognized that seed yield per hectare is mainly associated with an increase in the percentage of seed germination, NP

and seed index, in contrary, prolonged flowering and delayed maturity in lentil crop reduces SY [44, 45]. Therefore, someone should be very careful about sowing time and its stage. The number of pods per peduncle had a significant positive correlation with SY and yield contributing traits like BY and HSW [16, 46].

In PCA contribution of variables toward traits revealed that SY, BY and PH had highest values in factor 1 which represent high variability among these traits whereas in factor 2 highest values were recorded in HSW, PH, and HS which are recommended for further selection and characterization. Similar results were observed in lentil collected from Pakistan and Turkey in the past [16, 17]. Scattered diagrams reflected high variation as spreads genotypes where PC showed that genotypes with better performance for SY, BY, PS, HSW, PH and HS displayed at extreme position. Previous studies showed that > 80 % of genetic variation is contributed through first two components [24, 47-48]. It was revealed that component factor 11 indicated high potential for both SY and BY, therefore this component is also suggested for further use. Similarly, factor 4 can be shortlisted for DM as well factor 7 for HSW and factor 8 for PS. Genotypes with best CT placed in factor 6 during 2017-18 and factor 2 during 2018-19. Correlation matrix for DM showed negative association with CT and supported by the fact that time of maturity always influenced by variation in environmental factors. Therefore, it is assumed that probably genotypes with best CT placed in different components during both of the years. Individual components can also be selected for NP, LPH, and PH which were placed in factors 3, 4, and 5, respectively. It can be derived that PCs which showed consistent performance in both years would be more beneficial to cater genotypes potential.

Hierarchical cluster analysis revealed that genotype 6015 (USA) and 5698 (Sialkot, Pakistan) showed an outlier from all other clusters in 2017-18 which indicated that these

genotypes performed well for DM. Genotypes 5664 (Sialkot, Pakistan) and 6074 (Bhawalnager, Pakistan) showed outliers from other clusters in 2018-19, resultantly placed in cluster 7. These genotypes performed best for the maximum number of quantitative traits as well as SY. It was also recorded that Syrian germplasm grouped into cluster 2 in 2018-19 while collections representing USA and Pakistan grouped into different clusters. On the other hand, genotypes of Syria, USA and Pakistan during 2017-18 were grouped in C2 clusters that might be due to common ancestry. It is assumed that distribution of genotypes during both of the years in separate clusters was due to environmental conditions especially temperature at experimental site or could be genetic material that carries genes of interest which can play significant role in lentil breeding program as well as in the improvement of these traits through selection [49-51]. Inter-cluster distance between genotypes of any two clusters indicated that these would be more genetically divergent [52, 53].

Another statistical approach used as structural equation model provides illustration of association among multifactorial traits which drives that SY is dependent on other traits in a casual way and path of the characters are towards SY. In the current findings, SEM showed significant level < 1 contribution of independent variables like LPH, PS, DM, HS, NP, NSP toward SY [16]. The high value of phenotypic coefficient of variance as compared to genotypic coefficient of variance indicated influence of environment on traits as well as it specified the possibility for selection of traits. Result showed that genotypic coefficient of variance was found to be less than the phenotypic coefficient of variance that might be due ~~to environmental factors influenced~~ the expression of the phenotypes. Most of the agro-morphological traits had fairly higher heritability [18]. A trait with high heritability indicates that these have more potential for ~~utilizing available genetic variability as~~ compared to those showing low values. Traits that

showed low level of heritability need further consideration like NSP. The high heritability value along with high genetic advance for SY, BY and LPH signifies the selection of these traits for further crop improvement [54]. It is assumed that a high genetic advance under selection could be achieved when characters are highly heritable and stable due to additive gene action. Traits with low heritability can be improved through selection using heterosis breeding [55, 56]. Values for phenotypic variance inferred those environmental factors played significant role as compared to genotypic variance for heritable quantitative traits [57]. Therefore, characters with high heritability are more important to gather maximum variation for starting varietal development program.

Conclusion

Genotype 6122 originated from Sheikhupura, Pakistan performed best for several traits during both years, hence recommended for farmers' field cultivation after conducting necessary trials. Yield per plant was recorded as 8.05 g and ultimately provides estimates of yield tons/hectare as 2.68, while check variety Markaz-09 have yield potential of 3.2 tons/hectare, advocates high future prospective for current lentil germplasm. Genotypes representing Pakistan and Syria exhibited best values for BY which possess potential to be used as fodder or forage purposes, too. Present investigation reported DM ranged from 161-198 days during both of the years while earlier reports showed more time for maturity. Short duration genotypes (5698, 6015, 5667, 5748, 5555, 6017, 5700, 5571, 5580, 5981 matured in < 178 days should be further tested for selecting early maturity lines. The high GCV along with heritability and genetic advance depicted wide variation for selection with improving yield traits in lentil cultivars. Ten genotypes

including 5664, 5687, 6084, 6062, 6122, 6058, 6087, 5689, 6042, and 6074 were selected based on high yield and recommended for future crop improvement programs.

Declaration

Not applicable

Funding

Not applicable

Conflicts of interest/competing interests

Authors declare no competing interests

Availability of data and material

All of the relevant data is already included

Authors' contributions

Conceptualization: Muhammad Sajjad Iqbal, Abdul Ghafoor, Data curation: Syed Atiq Hussain, Formal analysis: Saba Munir, Ayesha Tahir[2], Methodology: Muhammad Akbar[1], Noshia Arshad[1]. Resources: Muhammad Ahson Khan[3], Muhammad Kashif Ilyas[3]. Software: Muhammad Azhar Ali[1], Syed Atiq Hussain. Supervision: Muhammad Sajjad Iqbal. Validation: Hajra Masood[1], Tahira Ahmad[1], Nazra Shaheen[1], Writing – original draft: Syed Atiq Hussain, Muhammad Sajjad Iqbal. Writing – review and editing: Muhammad Sajjad Iqbal and Abdul Ghafoor.

Consent to participate

Not applicable

Consent for publication

All authors are willing to publish this manuscript in this journal

References

1. Ates D, Aldemir S, Yagmur B, Kahraman A, Ozkan H, Vandenberg A, et al. QTL mapping of genome regions controlling manganese uptake in lentil seed. G3. 2018; 8: 1409-1416.
2. Ates D. Genetic diversity in lentil landraces revealed by diversity array technology (DArT). Turkish J. Field Crops. 2019; 24: 252-260.
3. Scippa GS, Trupiano D, Rocco M, Viscosi V, Di Michele M, Dandrea A, et al. An integrated approach to characterization of two autochthonous lentil (*Lens culinaris*) landraces of Molise (South-Central Italy). Heredity. 2008; 101: 136-144.
4. Thavarajah P. Evaluation of chickpea (*Cicer arietinum* L.) micronutrient composition: Biofortification opportunities to combat global micronutrient malnutrition. Food Res. Intern. 2012; 49; 99-104.
5. Mbasani MJ, Briache FZ, Ennami M, Gaboun F, Benbrahim N, Triqui ZEA, et al. Resistance of Moroccan lentil genotypes to *Orobanche crenata* infestation. J. Crop Improve. 2019; 33: 306-326.

6. Pandey S, Bhatore A. Genetic diversity analysis for quantitative traits in indigenous genotypes of lentil in Madhya Pradesh. J. Pharmacog. Phytochem. 2018; **7**, 279-283.

7. Gaad D, Laouar M, Abdelguerfi A, Gaboun F. Collection and agromorphological characterization of Algerian accessions of lentil (*Lens culinaris*). Biodiversitas J. Biol. Divers. 2018; 19: 183-193.

8. Ramírez OAM, Moreno RR, Camara MF. Mineral and trace element content in legumes (lentils, chickpeas and beans): Bioaccesibility and probabilistic assessment of the dietary intake. J. Food Composition and Analysis, 2018; 73: 17-28.

9. Shi L, Arntfield SD, Nickerson M. Changes in levels of phytic acid, lectins and oxalates during soaking and cooking of Canadian pulses. Food Res. Intern. 2018; 107:660-8.

10. FAOSTAT. http://www.fao.org/faostat/en/#data. 2019; Accessed 26 March 2019.

11. Noor R, Khan SM, Ahmad F, Hussain M, Abd-Allah EF, Alqarawi AA, et al. Morpho-agronomic characterization study of *Lens culinaris* genotypes under salt marsh habitat in Swat, Pakistan. Saudi J. Biol. Sci. 2017; 24: 1639-1645.

12. Khan A, Ihsan M, Nisar M, Hazrat A, Ali M, Ul-Haq R, et al. Evaluation of genetic diversity in barley landraces through agromorphological and Biochemical Characterization. Sarhad J. Agri. 2011; 37: 984-992.

13. Ullah J, Hussain SA, Nisar M, Khan W, Khan U, Khan J et al. Biochemical characterization of lentil genotypes for genetic diversity. Plant Cell Biotech. Mol. Biol. 2016; 17: 7-13.

14. Gupta R, Begum SN, Islam MM, Alam MS. Characterization of lentil (*Lens culinaris* M.) genotypes through phenotypic marker. J. Bangladesh Agri. Uni. 2012; 10: 197-204.

15. Cristobal MD, Pando V, Herrero B. Morphological characterization of lentil (*Lens culinaris* Medik.) landraces from Castilla y León, Spain. Pakistan. J. Bot. 2014; 46: 1373-1380.
16. Bilal M, Jamil M, Ali A, Ilyas MK, Ghafoor A. Multivariate analysis of genetic diversity in lentil (*Lens culinaris*) germplasm. Fres. Environ. Bull. 2018; 27: 2151-2159.
17. Mohammed NA, Refay YA, Migdadi HM, AL-Somain BH, Muharram AA, Al-Selwey, et al. Agromorphological characterization of lentil genotypes in dry environments. Intern. J. Agri. 2019; 22: 1320-1330.
18. Naoura G, Sawadogo N, Atchozou EA, Emendack Y, Hassan MA, Reoungal. Assessment of agromorphological variability of dry season sorghum cultivars in Chad as novel sources of drought tolerance. Scientific Rep. 2019; 9: 1-12.
19. Santos CS, Silva B, Valente LMP, Gruber S, Vasconcelos MW. The effect of sprouting in lentil (*Lens culinaris*) nutritional and microbiological profile. Foods. 2020; 9: 400.
20. Iqbal MS, Akbar M. Phenotypic diversity analysis and performance of elite lines of *Brassica napus* L. Bangladesh J. Bot. 2021; 50(1): 1-6.
21. Nisar M, Ghafoor A, Ahmad H, Khan MR, Qureshi AS, Ali H, Islam M. Evaluation of genetic diversity of pea germplasm through phenotypic trait analysis. Pakistan. J. Bot. 2008; 40(5): 2081-2086.
22. Ghafoor A, Arshad M. Multivariate analyses for quantitative traits to determine genetic diversity of blackgram [*Vigna mungo* (L.) Hepper] germplasm. Pakistan. J. Bot. 2008; 40(6): 2307-2313.

23. Tucak M, Popović S, Cupic T, Šimić G, Gantner R, Meglic V. Evaluation of alfalfa germplasm collection by multivariate analysis based on phenotypic traits. Romanian Agri. Res. 2009; 26: 47-52.

24. Iqbal MS, Akbar M, Akhtar S, Fatima S, Ghafoor A. Multivariate analysis and its application for characterizing cowpea landraces. Legume Res. 2021; 44 (1): 1-7.

25. Iqbal Z, Arshad MG, Ashraf M, Mahmood T, Waheed A. Evaluation of soybean [*Glycine Max* (L.) Merrill] germplasm for some important morphological traits using multivariate analysis. Pakistan. J. Bot. 2008; 40(6):2323-2328.

26. Roy S, Islam MA, Sarker A, Malek MA, Rafii MY, Ismail MR. Determination of genetic diversity in lentil germplasm based on quantitative traits. AJCS. 2013; 7(1):14-21.

27. Iqbal MS, Qureshi AS, Ghafoor A, Qayyum A. Identification of superior genotypes based on morphological, physiological and agronomic traits in local and exotic cowpea germplasm. Pakistan. J. Bot. 2003; 35(1):69-77.

28. Singh RK, Choudhury BD. Biometrical method in quantitative genetic analysis. Kalyani Publishers, Ludhiana, New Delhi, 1985; pp. 54-57.

29. Popat R, Patel R, Parmer D. Variability: Genetic variability analysis for plant breeding research. R package version. http://cran.rproject.org/web/packages/variability/index.html. 2020. Accessed 07 September 2021.

30. Hussain N, Yaqoob M, Rashid A. Genetic competition among lentil (*Lens culinaris*) candidate lines for yield and yield components under rainfed conditions. J. Agri. Res. 2014; 52.

31. Iqbal MS, Ghafoor A, Abbasi FM, Qureshi AS, Ahmad H. Study of nutritional characteristics, mineral nutrients and agrobiodiversity in black cumin (*Nigella sativa* L.) genotypes from Pakistan. Afri. J. Biotech. 2011; 10: 14757-14766.

32. Paliya S, Saxena A, Tikle AN, Singh M, Tilwari A. Genetic divergence and character association of seed yield and component traits of lentil (*Lens culinaris* M.). Adv. Biores. 2015; 6: 53-59.

33. Bhartiya A, Aditya JP, Singh S. Assessment of variability for agromorphological traits in elite lentil (*Lens culinaris*) lines using multivariate analysis. Indian J. Agri. Res. 2015; 49: 539-543.

34. Ghanem, ME, Marrou H, Soltani A, Kumar S, Sinclair TR. Lentil variation in phenology and yield evaluated with a model. Agron. J. 2015; 107: 1967-1977.

35. Lee YJ, Sung JK, Lee SB, Lim JE, Song YS, Lee DB. Yield and free sugar contents of burdock (*Arctium lappa* L.) depending on nitrogen levels. Korean J. Soil Sci. Fertilizer. 2016; 49: 677-681.

36. Roy S, Islam MA, Sarker A, Ismail MR, Rafii MY, Mondal MMA, Malek MA. Morphological characterization of lentil accessions: qualitative characters. Bangladesh J. Bot. 2012; 41: 187-190.

37. Sita K, Sehgal A, Kumar J, Kumar S, Singh S, Siddique K H, Nayyar H. Identification of high temperature tolerant lentil (*Lens culinaris* Medik.) genotypes through leaf and pollen traits. Front. Plant Sci. 2017; 8: 744.

38. Kumar S, Hamwieh A, Manickavelu A, Kumar J, Sharma TR, Baum M. Advances in lentil genomics. In: *Legumes in Omic*. Springer Science + Business Media, New York, USA. 2014; 111-130.

39. Ma Y, Dias MC, Freitas H. Drought and salinity stress responses and microbe-induced tolerance in plants. Frontiers in Plant Sci. 2020; 11: 1750.
40. Pushpavalli R, Zaman-Allah M, Turner NC, Baddam R, Rao MV, Vadez V. Higher flower and seed number leads to higher yield under water stress conditions imposed during reproduction in chickpea. Functional Plant Biol. 2014; 42: 162-174.
41. Nadeem M, Li J, Yahya M, Sher A, Ma C, Wang X, et al. Research progress and perspective on drought stress in legumes: a review. Intern. J. Mole Sci. 2019; 20: 2541.
42. Santos CS, Carbas B, Castanho A, Bronze MR, Serrano C, Vasconcelos MW, Brites C. Relationship between seed traits and pasting and cooking behavior in a pulse germplasm collection. Crop and Pasture Sci. 2018; 69: 892-903.
43. Mirali M, Purves RW, Stonehouse R, Song R, Bett K, Vandenberg A. Genetics and biochemistry of zero-tannin lentils. PLoS One. 2016; 11(10), e0164624.
44. Dixit GP, Katiyar PK, Singh BB. Characterization of lentil (*Lens culinaris* Medik.) varieties based on morphological traits. J. Food Leg. 2011; 24: 194-197.
45. Dugassa A, Legesse H, Geleta N. Genetic variability, yield and yield associations of lentil (*Lens culinaris* Medic.) genotypes grown at Gitilo Najo, Western Ethiopia. Sci. Tech. Arts Res. J. 2015; 3: 10-18.
46. Depar MS, Soomro NA, Chandio AS, Samoon HA, Meghwar BL, Pathan AK, Kalroo MW. Evaluation of morpho-yield traits and their correlation with seed yield in lentil (*Lens culinaris*) genotypes. Sci. Intern. 2016; 28.
47. Abo-Hegazy SRE, Selim T, El-Emam EAA. Correlation and path coefficient analyses of yield and some yield components in lentil. Egyptian J. Plant Breed. 2012; 203: 1-14.

48. Shrestha J, Subedi S. Improving crop productivity through sustainable intensification. South Asian Res. J. Agri. Fish. 2019; 1: 8-11.

49. Afuape SO, Okocha PI, Njoku D. Multivariate assessment of the agromorphological variability and yield components among sweet potato (*Ipomoea batatas* (L. Lam) landraces. African J. Plant Sci. 2011; 5: 123-132.

50. Benbrahim N, Taghouti M, Zouahri A, Gaboun F. On-farm conservation of Zaer lentil landrace in context of climate change and improved varieties competition. Univ. J. Agric. Res. 2017; 5: 27–38.

51. Idrissi O, Piergiovanni A, Toklu F, Houasli C, Udupa S, De Keyser E, De Riek J. Molecular variance and population structure of lentil (*Lens culinaris* Medik.) landraces from Mediterranean countries as revealed by simple sequence repeat DNA markers: Implications for conservation and use. Plant Genet. Resour. 2018; 16: 249-259.

52. Raza I, Masood MA, Abid S, Farooq M A, Mustafa R. Exploring relationship among quantitative traits of sugarcane varieties using principal component analysis. Sci. Tech. Develop. 2017; 36: 142-146.

53. Nath UK, Rani S, Paul MR, Alam MN, Horneburg B. Selection of superior lentil (*Lens esculenta* M.) genotypes by assessing character association and genetic diversity. The Sci. World J. 2014; 6.

54. Tyagi SD, Khan MH. Studies on genetic variability and interrelationship among different traits in Microsperma lentil (*Lens culinaris* Medik.). J. Agri Biotech Sustain. Devel. 2010; 2: 15.

55. Asghar MJ, Abbas G, Shah TM, Atta BM. Study of genetic diversity in some local and exotic lentil (*Lens culinaris* Medik) genotypes. Pakistan. J. Bot. 2010; 42: 2681-2690.

56. Bicer BT, Sakar D. Heritability of yield and its components in lentil (*Lens culinaris* Medik.). Bulgarian J. Agri. Sci. 2010; 16: 30-35.

57. Sarfraz Z, Shah MM, Iqbal MS. Genetic variability, heritability and genetic advance for agronomic traits among genome donor wheat genotypes. J. Agric. Res. 2016; 54: 15-20.

Supporting information

S1 Table. List of lentil genotypes studied during 2017-18 and 2018-19. Two varieties Markaz 2009 and Punjab 2009 were used as check.

S2 Table. Mean±SE for quantitative traits of 5% best performing lentil genotypes during 2017-18.

S3 Table. Mean±SE for quantitative traits of 5% best performing lentil genotypes during 2018-19.

S4 Table. Principal component analysis for quantitative traits of lentil studied during 2017-18.

S5 Table. Principal Component Analysis for quantitative traits studied during 2018-19.

S1 Fig. Average temperature during lentil crop season October to April 2017-18 and 2018-19 at the experimental station

Figure Click here to access/download;Figure;Fig. 1.docx

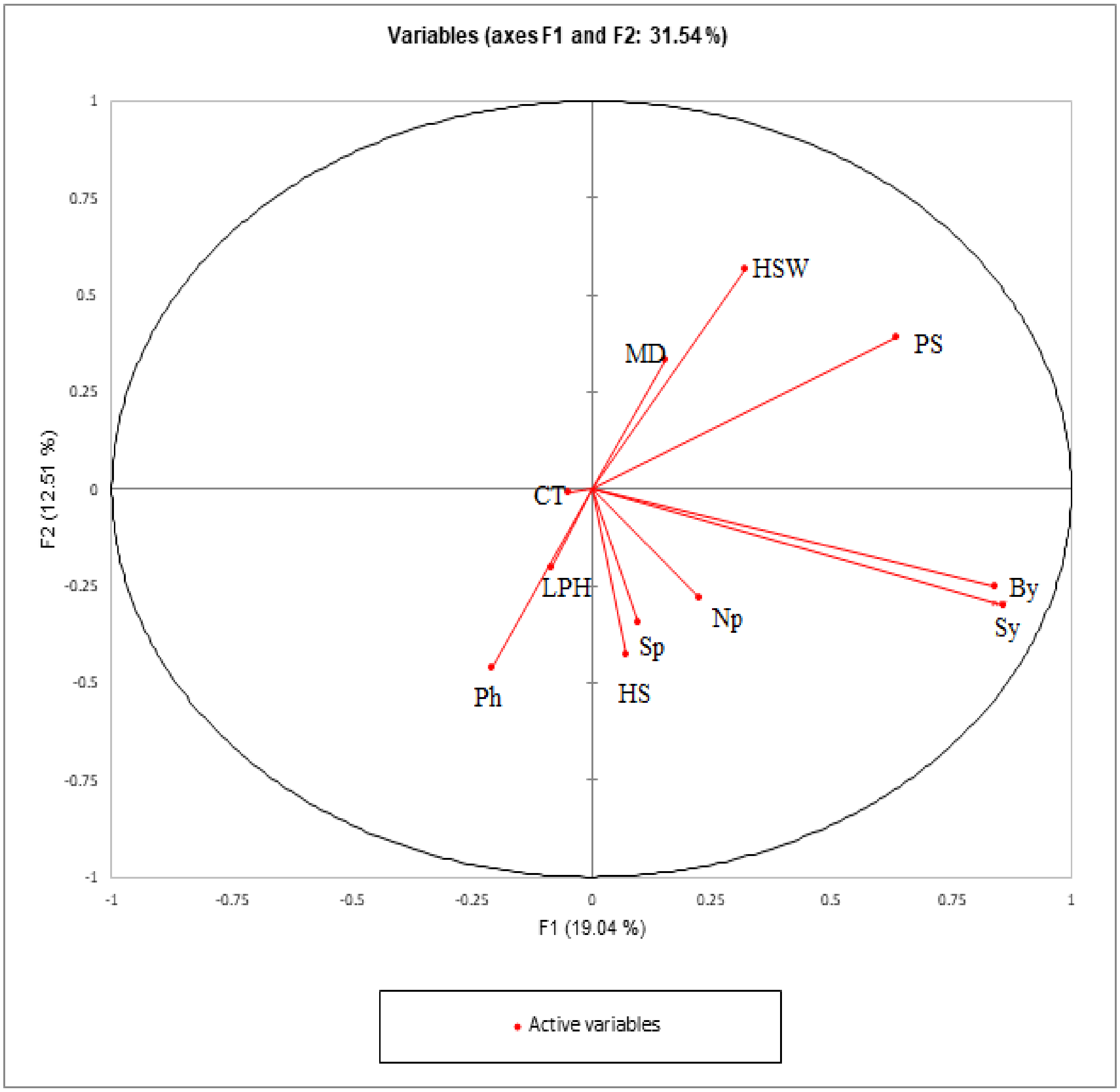

Fig. 1. PCA showing the contribution of quantitative traits among 220 lentil genotypes grown in 2017-18.

Figure
Click here to access/download;Figure;Fig. 2.docx

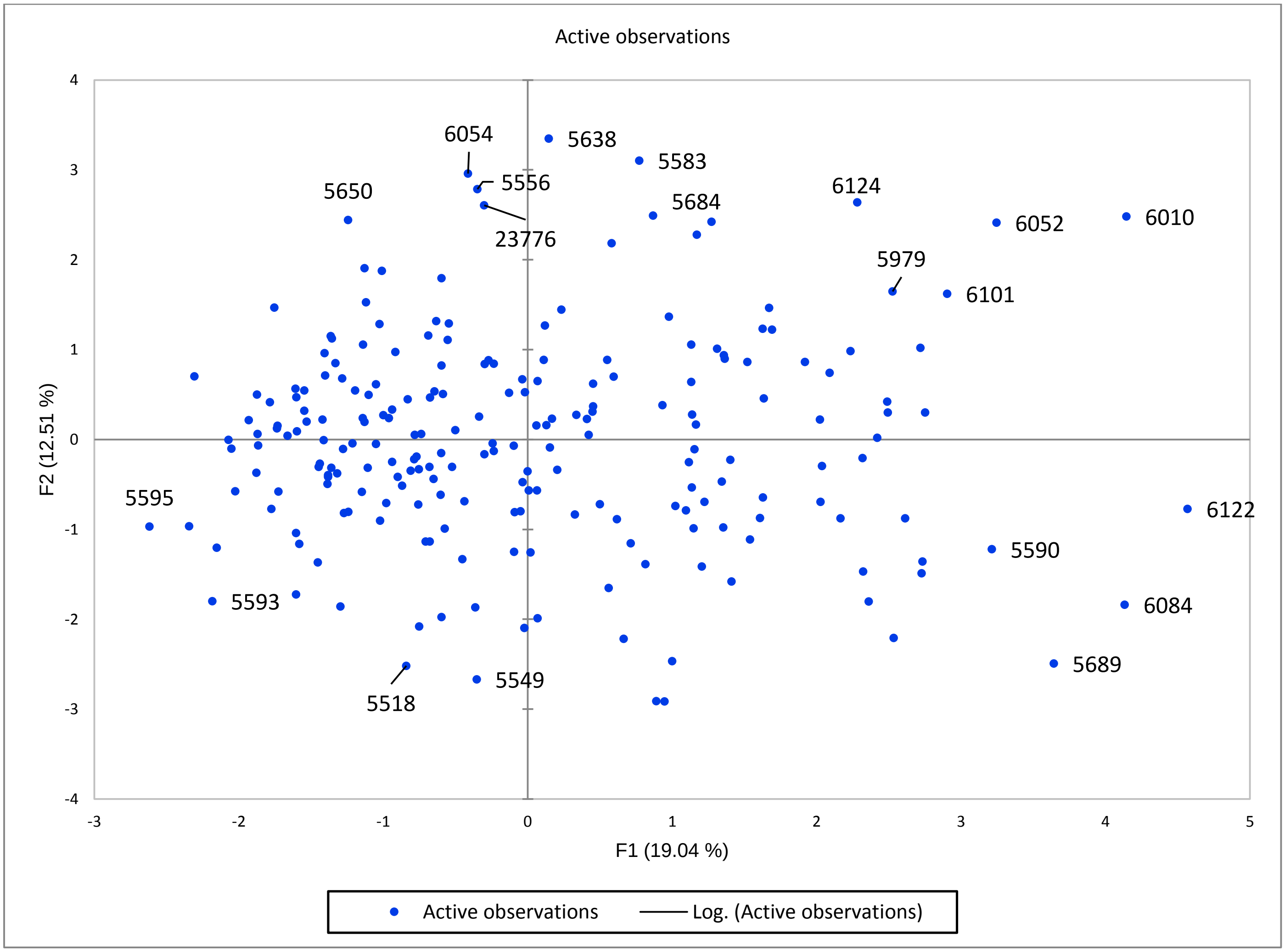

Fig. 2. Scattered plot of PCA for 220 lentil genotypes studied during 2017-18.

Figure Click here to access/download;Figure;Fig. 3.docx

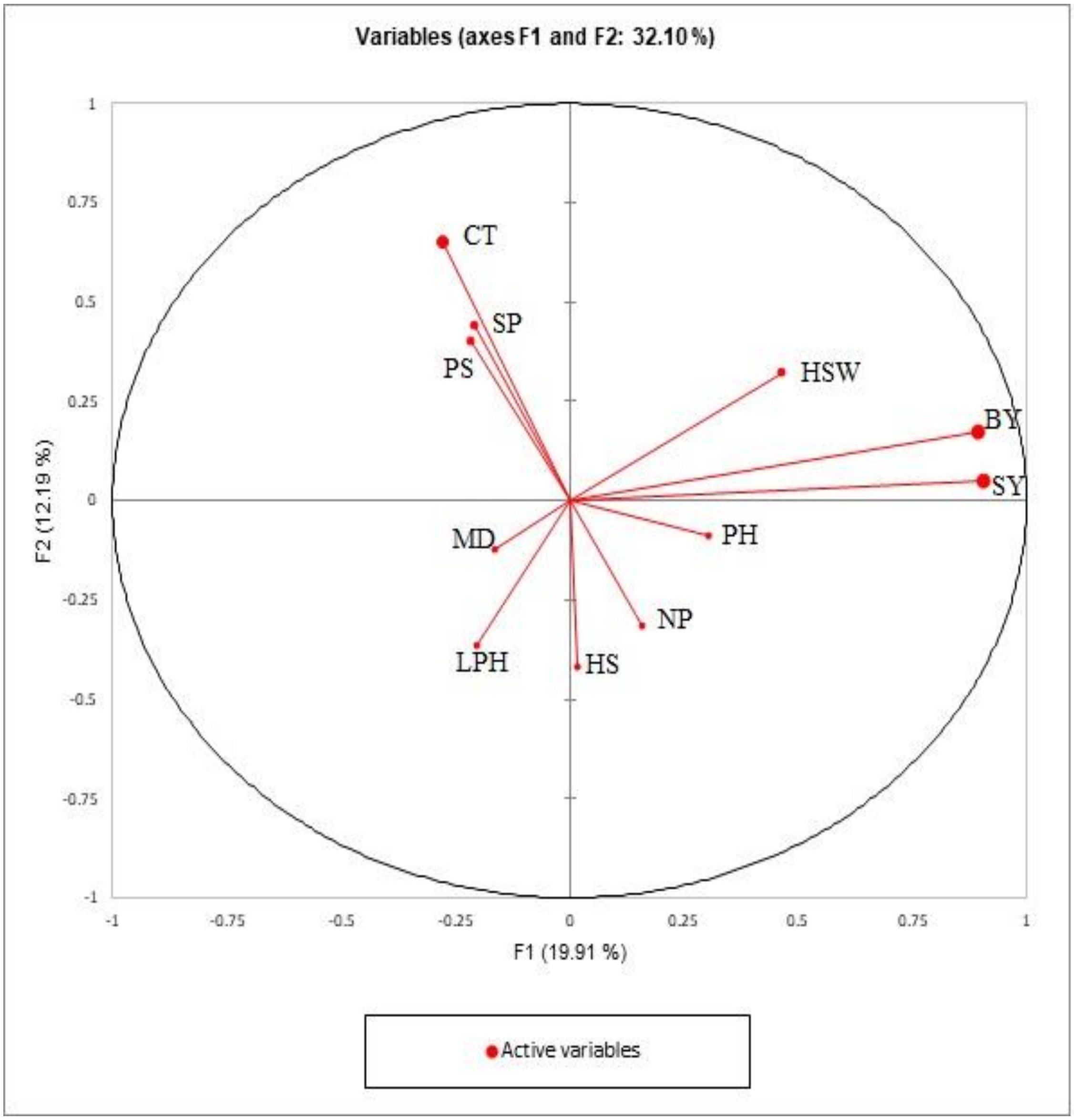

Fig. 3. PCA showing the contribution of quantitative traits among 220 lentil genotypes grown in 2018-19.

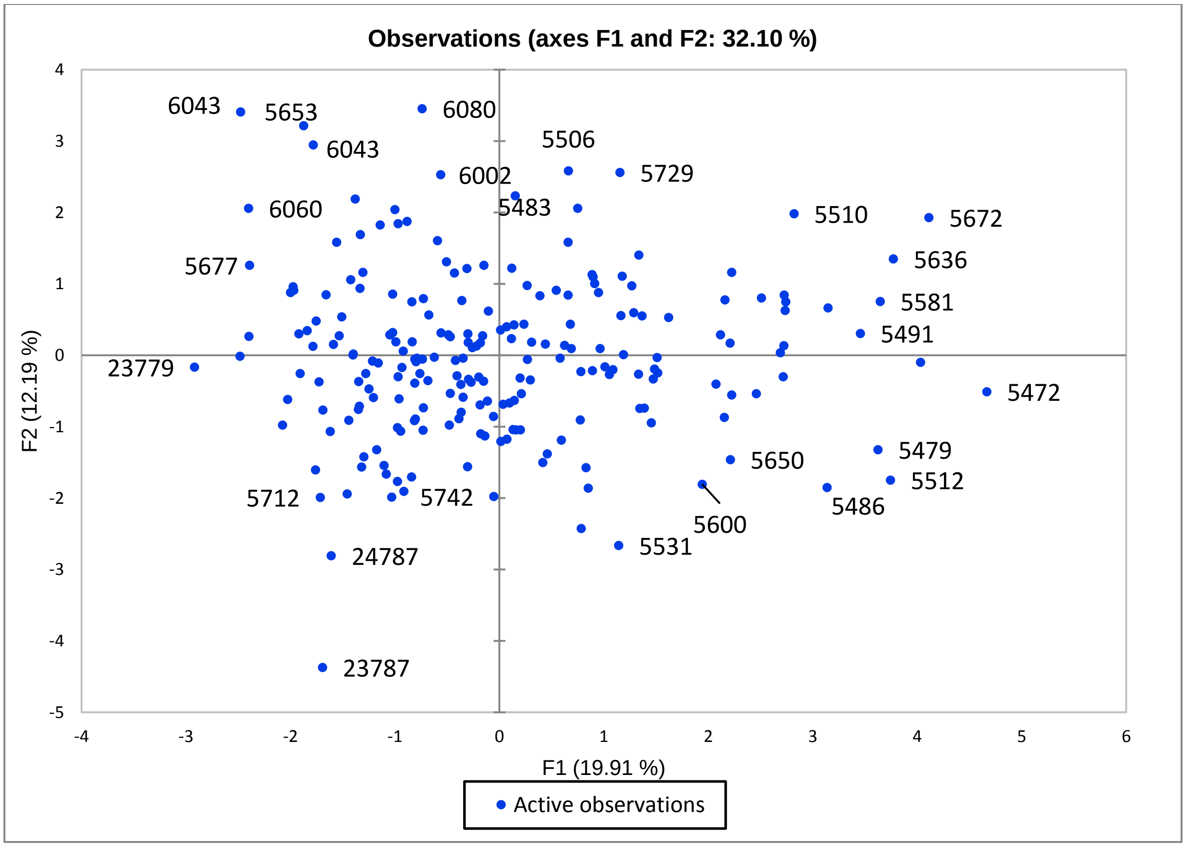

Fig. 4. Scattered plot of PCA for lentil genotypes grown in 2018-19

Figure
Click here to access/download;Figure;Fig. 5.docx

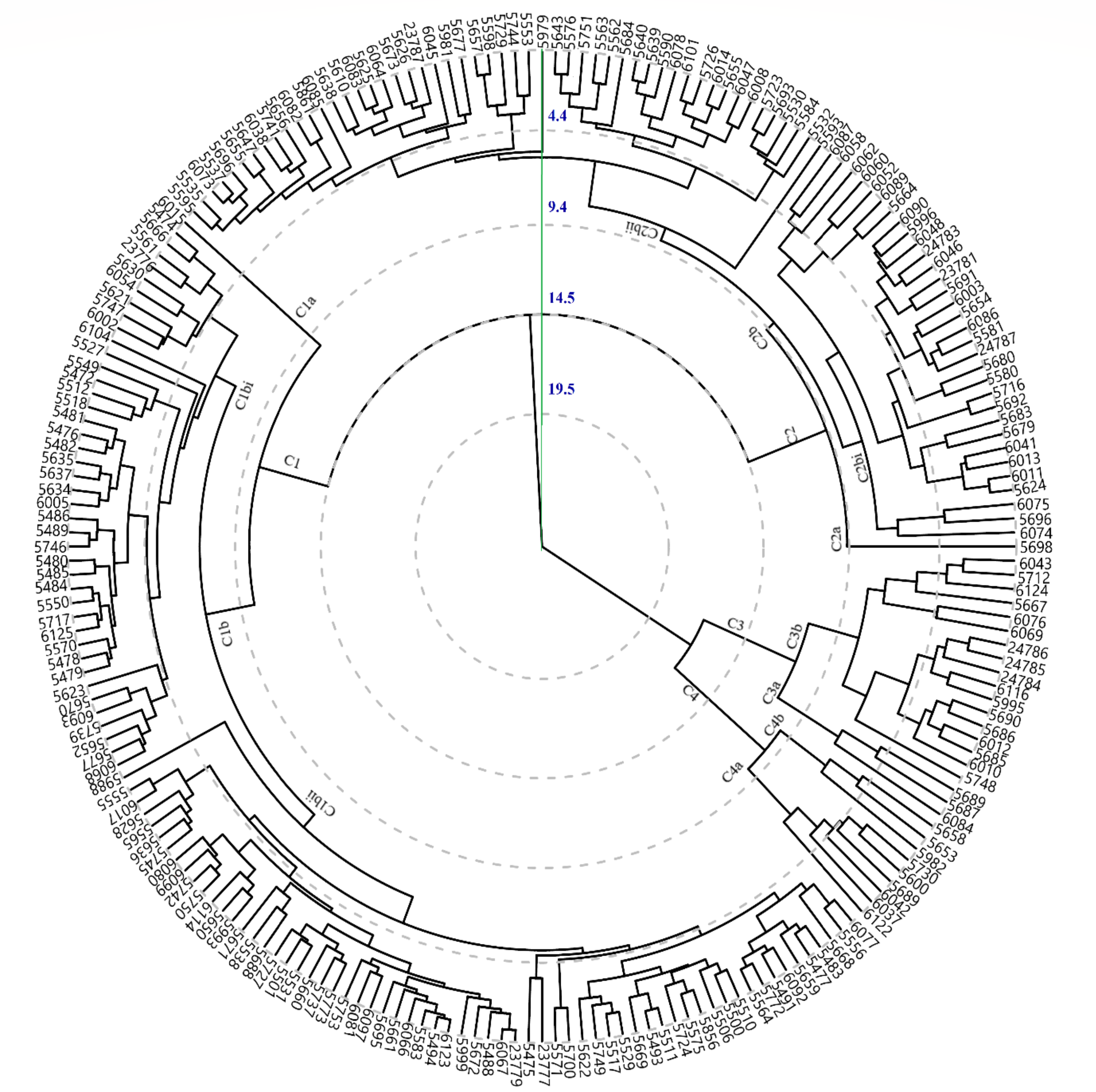

Fig. 5. Hierarchical dendrogram showing the relationship of lentil genotypes for quantitative traits studied during 2017-18.

Figure Click here to access/download;Figure;Fig. 6.docx

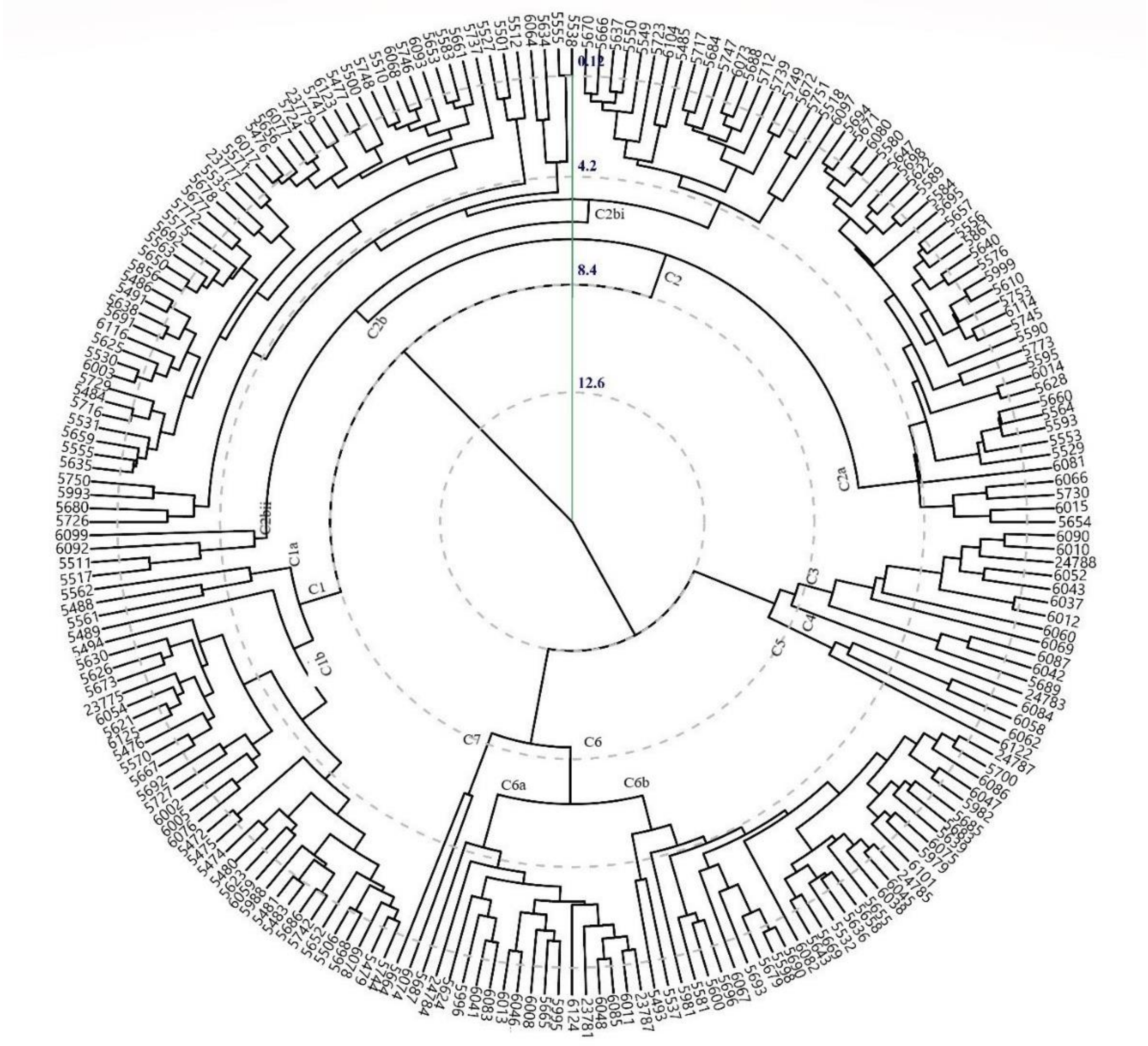

Fig. 6. Hierarchical dendrogram showing the relationship of lentil genotypes for quantitative traits studied during 2018-19.

Figure
Click here to access/download;Figure;Fig. 7.docx

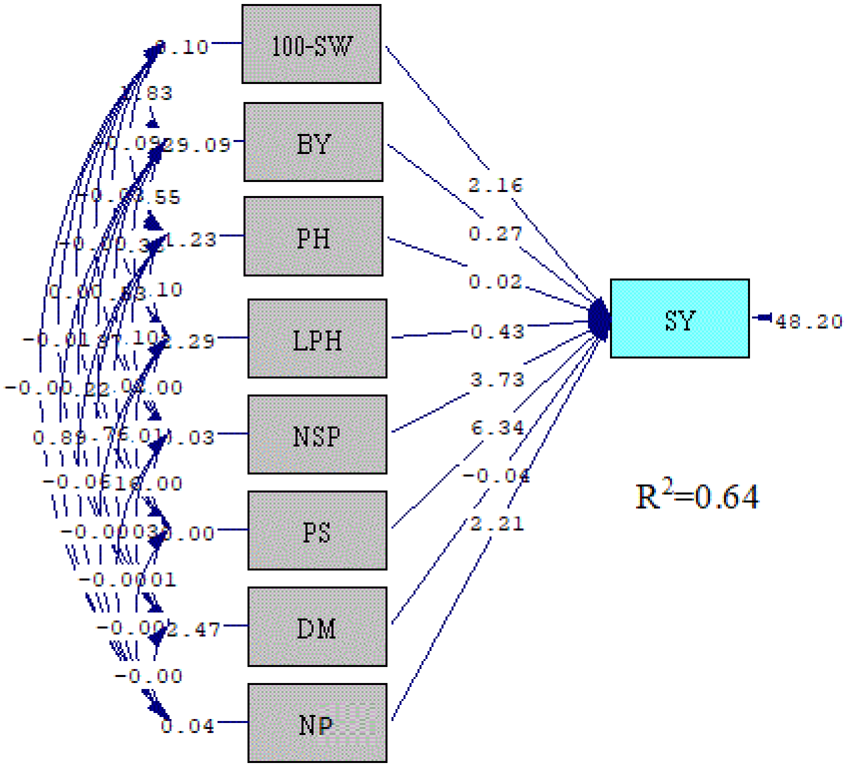

Chi-Square=0.00, df=0, P-value=1.00000, RMSEA=0.000

Fig. 7. Structural equations model for seed yield (SY) during 2017-18 and 2018-19

Click here to access/download
Supporting Information
S1 Fig..docx

Click here to access/download
Supporting Information
S1 Table.docx

Click here to access/download
Supporting Information
S2 Table.docx

Click here to access/download
Supporting Information
S3 Table.docx

Click here to access/download
Supporting Information
S4 Table.docx

Click here to access/download
Supporting Information
S5 Table.docx

Estimating genetic variability among diverse lentil collections through novel multivariate techniques

Formatted: Font: 18 pt

Syed Atiq Hussain[1], Muhammad Sajjad Iqbal[1]*, Muhammad Akbar[1], Noshia Arshad[1], Saba Munir[1], Muhammad Azhar Ali[1], Hajra Masood[1], Tahira Ahmad[1], Nazra Shaheen[1], Ayesha Tahir[2], Muhammad Ahson Khan[3], Muhammad Kashif Ilyas[3], and Abdul Ghafoor[3]

[1]Biodiversity Informatics, Genomics and Post Harvest Biology Laboratory, Department of Botany, University of Gujrat, Gujrat, 50700-Pakistan;

[2]Departmet of Biosciences, COMSATS University, Chak Shahzad Campus, Park Road, Islamabad;

[3]National Agricultural Research Center (NARC), Islamabad, Pakistan

*****Correspondence: drsajjad.iqbal@uog.edu.pk (MSI)

These authors contributed equally to this work.

Acknowledgement

We are ~~great~~grateful ~~ly thankful~~ to Bio-~~resource~~ Resource Conservation Institute (BCI), NARC, Islamabad, Pakistan for providing Lentil germplasm and experimental field facilities.

Abstract

Lentil is an important food legume throughout the world and Pakistan stands at 18th position with 8,610 tons production from 17,457 hectares. It is rich in protein, carbohydrates, fat, fiber, and minerals that can potentially meet food security and resolve malnutrition issues, particularly in South Asia. Two hundred and twenty lentil genotypes representing Pakistan (178), Syria (14), and USA (22) including 6 from unknown origin were studied for yield, yield contributing traits, and cooking time (CT). The genotype 6122 (Pakistan) performed the best during both years with seed yield per plant (SY) 68±1.7 g, biological yield per plant (BY) 264±2.8 g, pod size (PS) 0.61±0.01 cm, number of seeds per pod (SP) 2, cooking time (CT) 11 minutes, with no hard seed (HS). The genotype 6122 (Pakistan) and 6042 (Syria) produced the highest BY, hence these have potential as an efficient source for fodder, particularly during extreme winter months. The genotype 5698 (Pakistan) and 6015 (USA) were late in maturity, during 2018-19 while 24783 and 5561 matured early in 2019. Minimum CT 10 minutes was taken by the genotypes 6074 and 5745 of Pakistani origin. The lowest CT saves energy, time, and resources, keeps flavor, texture and improves protein digestibility, hence the genotypes with minimum CT could be short listed for developing better lentil cultivars. Pearson correlation matrix revealed significant association among several traits, especially SY with BY, PS, and NSP that suggestsed their use for the future crop improvement program. The PCA revealed a considerable reduction in components for selection of suitable genotypes with desired traits that could be utilized for future lentil breeding. Structural Equational Model (SEM) for SY based on covariance studies indicated the perfect relationship among variables. Further, hierarchical cluster analysis establishes four clusters for 2017-18, whereas seven clusters for 2018-19. Cluster 4 of 2017-18 and cluster 5 of 2018-19 exhibited the genotypes with best performance for SY, BY, PS, NSP, CT and HS. Based on

Formatted: Font: 18 pt

Formatted: Font: 18 pt

heritability; HSW, SY, BY, NPS were found highly heritable. Traits with high heritability will be helpful for selecting genotypes ~~high in variation~~ which ~~could~~ exhibit genes of interest and efficient in improvement of the particular trait. In conclusion, 10 genotypes (5664, 5687, 6084, 6062, 6122, 6058, 6087, 5689, 6042~~,~~ and 6074) have been identified hence suggested for future ~~their use for~~ lentil ~~crop~~ breeding programs.

Introduction

Formatted: Font: 18 pt

Lentil (*Lens culinaris* Medik.) is an annual, herbaceous, cool-season, autogamous (self-pollinated) and diploid legume with 4 Gbp genome size ~~[12]~~ that was domesticated with wheat and barley in the Fertile ~~crescent~~Crescent [1, 2]. It is one of the oldest dry legumes domesticated about 9,000 years ago in the Near East and subsequently spread throughout the Mediterranean Basin and Central Asia [3]. Lentil is the 4th major pulse crop in the world after common bean, pea~~,~~ and chickpea, and included seven taxa with four species such as *L. culinaris*, *L. nigricans*, *L. ervoides*~~,~~ and *L. lamottei*, the only cultivated species (*L. culinaris*) have two varietal types as macro-sperma (large seeded) and micro-sperma (small seeded) [4, 5].

Lentil is consumed due to its highest protein contents, vitamins~~,~~ and micronutrients that are a vital part of human nutrition while vegetative parts are being used as fodder for ~~animals~~ livestock [6]. Protein in lentil seed is of high quality ~~that~~ which is 22-35% of total seed mass and consisted of a large number of amino acids including lysine and isoleucine, hence it is a cheap source of protein in many regions of the world both for humans as well as for animals [7].

Additionally, lentil is a rich source of carbohydrates (59%), minerals (2%)~~,~~ and fiber with ~~having~~ very little fat which ~~that~~ is significant to overcome malnutrition and deficiency of micronutrients in developing countries [8]. It is also the best source of iron to prevent iron deficiency [9]. Medicinally it has low glycemic content~~level~~, due to this ~~hence~~ physicians strongly suggest ~~it~~ for patients with diabetes, obesity~~,~~ and heart ~~disorders~~maladies [9]. Seeds of lentil~~s~~ are mucilaginous and laxative, recommended for treating constipation, stomach infections, smallpox~~,~~ and ulcer [6]. The top ten lentil producing countries are Canada, India, Turkey, USA, Kazakhstan, Nepal, Australia, Russia, Bangladesh and China [10].

The imperative causes of the low yield of lentil in Pakistan are cultivation on marginal lands with poor management practices, lack of high yielding varieties, biotic and abiotic stresses, and above all adaptation of low yielding genotypes in different environments with minimum genetic variability ~~is the~~ with critically ~~factor for its~~ low productivity [11, 12]. It is ~~not~~ economically not feasible to reduce biotic stresses~~ors~~ by utilizing pesticides, herbicides and to reduce abiotic stresses by supplementing soil to meet plant demand through reclamation and enhancing fertilizer, as well as by improving irrigation systems [13]. Alternatively, ~~Therefore,~~ the best solution to overcome these problems is to develop ~~make~~ superior planting material. Regarding ~~In~~ this~~ regard~~, ~~plant~~ breeding and modern bio-techniques could improve crops by transferring a gene of interest or genetic modification. The ~~insufficient~~inadequate information of genetic variability in existing lentil is a major hindrance to harvesting the potential ~~of the crop~~ including inadequate use of modern molecular techniques for genetic improvement [13].

Several studies have been carried out ~~conducted~~ worldwide to assess genetic diversity based on phenotypic markers in lentil as well other legumes in the past [3, 7, 11, 14-~~18,~~ 19]. Interestingly, g~~G~~ermplasm repository is a valuable asset that carries a huge treasure of genetic

material for future crop improvement and development programs [20]. As genetic diversity can be estimated with the help of qualitative and quantitative traits, likewise, a large number of morphometric techniques have been used to measure and classify genotypes in mustard, peas, mungbean, alfalfa, cowpea, soybean~~,~~ and lentil [21-26]. ~~The c~~Current studies ~~employed~~engaged ~~used~~ several statistical techniques and aiming to unwind the hidden potential of genetic diversity in the available lentil genetic resources and to assess variability in agro-morphological traits for consecutively two years. Identified novel genotypes would be the base material for future lentil breeding programs.

Material and Methods

Formatted: Font: 18 pt

Ethics statement on experimental research

Formatted: Font: 16 pt

The study was approved by the Institutional Committee through No. UOG/ASRB/Botany/03/15806. Accordingly, all of the current experimental research and field studies comply with relevant institutional, national~~,~~ and international guidelines and legislation.

Plant material, study site, research design, and crop cultivation

Formatted: Font: 16 pt

Two hundred and twenty lentil genotypes representing Pakistan, the USA~~,~~ and Syria including unknown origin were ~~acquired~~ obtained from National Gene bank, National Agriculturale Research Center (NARC), Islamabad, Pakistan (S1 Table). Germplasm ~~was~~ planted under field conditions of NARC in Islamabad, Pakistan located at 33.6701° N latitude

and 73.1261° E longitude during October 2017 and harvested in April 2018, similarly, ~~and~~ same timing and procedure were followed in the second year 2018-19. Both qualitative and quantitative traits were recorded consecutively for two years (2017-18 and 2018-19). While meteorological data ~~were~~ recorded on monthly basis was obtained ~~at the~~ from the experimental station (S1 Fig.). Experiments were laid out in an augmented design within 11 blocks and each block consisted of 20 genotypes along two check varieties, *viz.*, Markaz 2009 and Punjab 2009 after every twenty lines [16]. Three rows of 5 m length in each bed were planted with 10 cm space between plants and 30 cm within rows~~ spacing~~. The trial was conducted under usual agricultural practices without applying any herbicide, fungicide, pesticide~~,~~ or fertilizer so that the actual genetic diversity may be studied. Soil was homogenously fertile and analyzed in ~~by~~ Soil Science Laboratory, NARC, Islamabad, which ~~that~~ was Nabipur series, coarse loamy, mixed, hyperthemic~~,~~ and udic ustochrepts. Recommended cultural practices were followed throughout the crop season [27].

Data collection

Formatted: Font: 14 pt

Ten healthy plants were randomly sampled from each genotype on a plant basis for recording of the data~~ on a plant basis~~. Quantitative traits were recorded at foliage, reproductive and ~~at~~ maturity stages including plant height (PH), number of pods per peduncle (NSP), lower pod height (LPH), pod size (PS), number of seeds per pod (NSP), and days to maturity (DM). Days to maturity were considered ~~counted~~ from the day ~~time~~ of sowing to turning of 90% pods into golden brown color. After harvesting, biological yield (BY), seed yield (SY), 100~~-~~ seed weight (HSW), cooking time (CT)~~,~~ and hard seeds (HS) were recorded ~~counted~~ after boiling 100 seeds. Cooking time was calculated ~~measured~~ in minutes for cooking un-soaked seeds to softness in boiling distil~~led~~ water.

Formatted: Font color: Text 1

For qualitative traits, observations were recorded for ground color of testa (GCT), cotyledon color (CC), color of pattern on testa (CPT), pod dehiscence (PD), pod shedding (PSh), leaflet size (LS), tendril length (TL), ground color of flower (GCF), pod pigmentation (PP), leaf pubescence (LP) and seedling stem pigmentation (SSP). At late vegetative stage, SSP ~~(~~designated as ‘0’ for absent and ‘1’ present~~)~~, LS ~~(~~‘3’ for small, ‘5’ medium and ‘7’ large~~)~~ and LP ~~(~~‘0’ absent, ‘3’ slight and ‘7’ dense~~)~~ ~~were observed~~. At early reproductive stage, GCF ~~(~~‘1’ white color, ‘2’ white with blue lines, ‘3’ blue, ‘4’ violet, ‘5’ pink and ‘6’ for others,~~)~~ were observed. At late reproductive stage, PP ~~(~~‘0’ for absent and ‘1' present~~)~~ were~~as~~ recorded. At the stage of ~~-~~maturity~~ stage~~, PD was categorized and recorded as ~~(~~‘0’ none, ‘3’ low, ‘5’ medium and ‘7’ high~~)~~ and PSh ~~(~~‘0’ none, ‘3’ low, ‘5’ medium and ‘7’ high.~~) were surveyed~~. Tendril length included ~~(~~‘1’ rudimentary tendril and ‘2’ for prominent tendril~~) were also observed during~~ at late reproductive stage. After harvesting, CPT ~~(~~‘0’ absent, ‘1’ olive, ‘2’ grey, ‘3’ brown, ‘4’ black.), CC ~~(~~‘1’ yellow color, ‘2’ orange-red~~,~~ and ‘3’ olive green) while ~~and~~ GCT ~~(‘~~represented ‘1’ green, ‘2’ grey, ‘3’ brown, ‘4’ black and ‘5’ pink~~) were observed~~.

Statistical analysis

Formatted: Font: 14 pt

Descriptive statistics of quantitative traits including mean, standard deviation, standard error of mean, range with minimum and maximum values, coefficient of variance, ~~,~~ Pearson correlation and PCA were performed ~~analyzed~~ through statistical software XLStat 2019. Hierarchical cluster analysis ~~was~~ conducted to construct dendrograms based on Euclidean distances using RStudio. The structural equation model (SEM) was drawn through LISREL (LISREL Student Version 9.2 https://lisrel-for-windows-student.software.informer.com/9.2/) to estimate multiple regressions that ~~were~~ indicating the contribution of quantitative traits toward yield. Moreover, the genotypic, phenotypic, environmental variances and broad sense heritability were estimated by the formulas

as provided below while genetic advance and genetic advance in~~as~~ percentage of mean were derived through variability package in R Studio [28, 29].

Genotypic variance

$$GV = \frac{GMS - EMS}{r}$$

Phenotypic variance

$$PV = \frac{GV - EV}{r}$$

Broad sense heritability

$$h^2 = \frac{GV}{PV}$$

Where GV and PV symbolized ~~denoted~~ as ~~are~~ the gen~~e~~otypic ~~–~~ and phenotypic variances, respectively; GMS, genetic mean square; EMS, error mean square; r, number of replications; EV, environmental variances and h^2 represented as broad sense heritability.

Result

Formatted: Font: 18 pt

Genetic variation based on quantitative traits

High genetic variability was observed among lentil germplasm which could broadened the scope of simple selection and opportunity for new recombinants for genetic improvement. Genotype 6122 collected from Sheikhupura, Pakistan ~~was~~ performed outstanding for SY (68±1.7 g), BY (264±2.8 g), PS (0.61±0.01cm), NSP (2), CT (11 minutes) with no HS. Mean values of SY (30.08±0.98 g) were higher in 2017-18 as compared to SY (25.26 ±0.87 g) in 2018-19 (Table 1). In comparison, check varieties showed low SY in 2018-19 as compared to ~~SY of~~ 2017-18. It

ranged from 5 to 85 g in 2017-18 and 3 to 76 g during 2018-19, respectively. It was noted that genotype 6084 belonged to Narowal, Pakistan produced the highest SY in both years while the lowest SY was recorded in genotype 5475 originated from Gujranwala, Pakistan for~~during~~ 2017-18. Likewise, genotype 5494 from Okara, Pakistan and 5475 from Gujranwala, Pakistan were the lowest in SY during 2018-19. Genotypes 6052 (Syria), 5583 (Muzaffargarh, Pakistan), and 5556 (Hyderabad, Pakistan) produced better SY for ~~during~~ both years. Overall performance of genotypes was highest during first year as compared to the subsequent year which could be attributed due to environmental conditions during second year. Evaluation based on both years with varying environmental conditions provide assistance in selection for not only common genotypes but also suggested varying environmental or location trials in future.

Biological yield is a significant contribution towards SY in lentil and the genotypes 6122 (Sheikhupura, Pakistan), 5689 (Sialkot, Pakistan), 5730 (Layyah, Pakistan), and 6037 (Syria) produced the highest BY 264±2.8 g, 258±1.0 g, 254±1.5 g, and 254±2.0 g, respectively in 2017-18, whereas genotypes 6042 (Syria), 6087 (Narowal, Pakistan) and 5689 (Sialkot, Pakistan) were the best among others for BY i.e., 187.0±1.3 g, 183.0±0.9 g, and 179±1.3 g, in 2018-19. Whereas plant height (PH) during 2018-19 was slightly lower than 2017-18, PH has significant contribution towards acquiring light and high number of branches to produce more seeds.

Table 1. Descriptive statistics for quantitative traits studied in lentil during 2017-18 and 2018-19**.**

		**2018-19**						**2017-18**					
**Traits**	**P values**	**Mean±SE**	**Min.**	**Max**	**SD**	**Markaz 2009**	**Punjab 2009**	**Mean±SE**	**Min.**	**Max.**	**SD**	**Markaz 2009**	**Punjab 2009**
SY	0.0001 s	30.08±0.9	5	85	14.52	41.72±1.0	55.27±2.4	25.26±0.8	3	76	12.91	39.08±0.9	53.27±1.7
HSW	0.254 ns	1.71±0.02	1.05	3.65	0.35	1.91±0.01	2.48±0.01	1.68±0.01	1.17	3.28	0.28	1.85±0.01	2.4±0.1
BY	0.0001 s	114.6±3.0	42	264	44.4	87.5±1.8	112.2±6.39	94.13±2.2	34	187	32.77	88.58±1.6	110.4±5.1
PH	0.0001 s	44.73±0.4	27.38	71.12	6.86	59.09±1.4	55.1±1.4	46.7±0.4	29.6	69	6.94	61.58±1.1	56.54±0.9
LPH	0.958 ns	14.97±0.1	9.14	32.16	2.34	15.32±0.4	15.6±0.36	14.98±0.1	10.72	33.34	2.03	16.56±0.6	16.71±0.6
PS	0.0001 s	0.3±0.01	0.2	0.7	0.08	1.55±0.20	1.36±0.01	0.3±0.01	0.2	0.7	0.08	1.33±0.01	1.37±0.01
NSP	0.0001 s	2.00±0.01	1	3	0.16	2±0.01	2±0.01	2.00±0.01	1	3	0.28	2±0.01	2±0.01
DM	0.0001 s	184±0.3	161	198	5.05	172±0.29	170±0.29	186±0.34	161	198	5.05	172.5±0.2	169±0.3
CT	0.0001 s	14.55±0.1	10	18	1.52	12.09±0.1	11.7±0.19	16.15±0.1	10	18	1.52	12.16±0.1	11.90±1.6
HS	0.0001 s	0.37±0.01	0	2	0.61	0.27±0.1	0.25±0.17	0.41±0.04	0	2	0.61	0.41±0.1	0.09±0.9
NP	0.0001 s	2.04±0.01	2	3	0.19	3±0.01	3±0.01	2.00±0.01	1	3	0.16	3±0.01	3±0.01

SY, seed yield; HSW, hundred seed weight; BY, biological yield; PH, plant height; LPH, lower pod height; PS, pod size; NSP, number of seed per pod; DM, days to maturity; CT, cooking time; HS, hard seed/hundred seed; NP, number of pods per peduncle; SE, standard error of mean; SD, standard deviation; p-value <0.05.

Days to maturity ranged from 161 to 198 days in both years. However, mean values were 184±0.34 days during 2017-18 and 186±0.34 days during 2018-19. Long duration genotypes were 5684 (Narowal, Pakistan), 5988 (USA), 5979 (USA), 5679 (Narowal, Pakistan), 5982 (Narowal, Pakistan), 5683 (Narowal, Pakistan), 24786 (unknown), 5623 (Faisalabad), 6077 (Bahawalnagar, Pakistan) and 5684 (Narowal, Pakistan) need further consideration to reduce the time for maturity, but these would be available for meeting fodder requirement for longer time. Following short duration genotypes (5698, 6015, 5667, 5748, 5555, 6017, 5700, 5571, 5580 and 5981 matured in <178 days. ~~Short duration genotypes are suggested as early maturing genotypes hence required further trials to see there early maturity cultivar status.~~

Both check varieties were insignificant within the same year, however overall performance was better during 2017-18 as compared to 2018-19. It indicates homogeneity of the experimental site and the validity of augmented design due to higher genetic variance. Superior genotypes based on the best performance for individual traits have been identified and presented in Table 2, which are recommended for future crop improvement programs. More than 40 g SY was produced by three genotypes 6084, 6062, and 6122 during both years, hence selected for ~~exploitation~~utilization of yield potential. Cooking time is very crucial particularly in the era of energy crises and higher fuel prices, hence short CT is an emerging selection criterion for most of the legumes including lentil. Keeping in view this important issue, seven genotypes *viz.*, 6074, 5595, 6075, 2018, 6092, 6099 and 5511 which took <12 minutes CT were short listed. A list of genotypes representing the best 5% of the germplasm including check varieties are ~~re~~presented (S2 Table & S3 Table).

Table 2. List of best performing lentil genotypes selected during both years for quantitative traits.

**Traits**	**During 2018**	**During 2019**

SY ≥ 40g	6084, 6062, 6122, 6058, 6087, 5689, 6042, 6074, 5664, 5687	6084, 6062, 6122, 6058, 6087, 5689, 6074, 6042, 5664,5687,
HSW ≥ 1.90g	6052, 5583, 5556, 6101, 5684, 23776, 5643	5583, 6052, 5684, 6101, 5556, 5643, 23776
BY ≥ 100g	6122, 5689, 6037, 6042, 5600, 5982, 5658, 6084	6122, 5689, 6037, 6042, 5600, 5982, 5658, 6084
PH ≥ 55	5549, 5687, 5723, 5549, 5687	5549, 5687, 5723, 5549, 5687
LPH ≥ 15cm	5527, 6062, 5751, 5686, 5744, 5477	5527, 6062, 5751, 5686, 5744, 5477
PS ≥ 0.4 cm	5664, 6124, 6116,6077, 6082	5664, 6124, 6116,6077, 6082
NSP ≥ 2	6122, 5687, 24784, 6012, 6084	6122, 5687, 24784, 6012, 6084
DM ≤ 180 days	24783,5741, 5729, 6081, 5981	24783, 5741, 5729, 6081, 5981
CT ≤ 12 minutes	6074,6075,6010, 6013, 6041	6074,6075,6010, 6013, 6041
HS ≤ 2	6074, 5595, 6075, 6010,6041	6074, 5595, 6075, 6010,6041
NP ≥ 2	5595,5861, 5691, 5671,5689	5595,5861, 5691, 5671,5689,

SY, seed yield; HSW, hundred seed weight; BY, biological yield; PH, plant height; LPH, lower pod height; PS, pod size; NSP, number of seed per pod; DM, days to maturity; CT, cooking time; HS, hard seed/hundred seed; NP, number of pods per peduncle

Genetic variation in qualitative traits

High variation was observed in qualitative traits as various lentil plant descriptors were described by dividing into categories (Table 3). The ground color of testa (GCT) exhibited four categories, out of which more than half of lentil germplasm have grey color, 33.6% as brown, 13.2% black while few genotypes (1.4%) were green. Cotyledon color (CC) was predominantly red, consumers' prefer red cotyledon color which have more market demand. ~~Traits which possess low variability must be required to address through new collection by broadening existing lentil genetic resources through local as well as exotic sources.~~

Table 3. Frequency distribution of qualitative traits of lentil germplasm studied during 2018 and 2019

Variable	No. of Categories	Categories Name	Frequency (%)	Variable	No. of Categories	Categories Name	Frequency (%)
Ground color of testa (GCT)	4	Green	1.36	Pod shedding (PS)	3	Low	55.9
		Grey	51.82			Medium	26.8
		Brown	33.64			High	17.3
		Black	13.18	Leaflet size (LS)	3	Small	62.73

Cotyledon color (CC)	3	Yellow	2.27			Medium	28.18
		Red	96.36			Large	9.09
		Olive green	1.36	Pod pigmentation (PP)	2	Absent	69.09
Color of pattern on testa (CPT)	5	Absent	0.91			Present	30.91
		Olive	7.73	Leaf pubescence (LP)	2	Slight	85.00
		Grey	59.54			Dense	15.00
		Brown	13.64	Seedling stem pigmentation.(SSP)	2	Absent	69.09
		Black	18.18			Present	30.91
Pod dehiscence (PD)	4	None	0.91				
		low	87.7				
		Medium	8.21				
		High	3.20				

Pearson correlation matrix

The correlation matrix for quantitative traits presented in Table 4 revealed positive association of SY with BY, PS, NSP, and NP. ~~HSW exhibited positive correlation with PS and BY which demonstrates that pod size and biological yield of the plant contributed positively if dense and high in number. Additionally, PH and PS also showed positive correlation with NSP and DM, respectively, which had also advocated that suitable plant height and pod size exhibit association with number of seeds per pods and days to maturity, such performance of the germplasm make convenient selection of traits for more positive associations.~~ Significant association among various traits showed effectiveness and contribution of yield and yield contributing traits in a symmetry that can be translated to boost yield and for selection of

genotypes with desirable traits. In the same context, lentil genotypes grown in 2018-19 indicated that SY exhibited a significant positive correlation with HSW, BY and PH, rests assure that these traits contributed directly towards seed yield. HSW was positively correlated with BY, in return BY was also positively correlated with PH. Desirable combinations described high potential of the germplasm that can be utilized in future crop improvement programs through selection.

Table 4. Pearson Correlation matrix for SY with other quantitative traits of lentil grown in 2017-18 and 2018-19

**Traits**	**Years**	**HSW**	**BY**	**PH**	**LPH**	**PS**	**NSP**	**DM**	**HS**	**NP**
**SY**	2018	0.09	0.73*	-0.09	0.03	0.32*	0.15*	0.15*	0.11	0.18*
	2019	0.27*	0.83*	0.21*	-0.16*	-0.11	-0.11	-0.05	-0.01	0.08

SY, seed yield; HSW, hundred seed weight; BY, biological yield; PH, plant height; LPH, lower pod height; PS, pod size; NSP, number of seed per pod; DM, days to maturity; HS, hard seed; NP, number of pods per peduncle

Principal component analysis (PCA)

The PCA for 2018 indicated that first five components have > 1.0 eigenvalue as 2.09, 1.37, 1.19, 1.08 and 1.02, respectively (S4 Table). SY, BY and PS contributed more toward component 1. Similarly, component 2 can be selected for HSW, PH and HS. PCA advocates further selection of genotypes with specific trait/s without losing total variation and mush better idea about evaluated genotypes. For second year selection can be made for factor 1 including SY (0.84), BY (0.84) and NP (0.02) which contributed highest factor loading values while factor 2 can be selected for HSW (0.32), PS (0.63) and DM (0.15) as shown in Fig. 1.

Fig. 1. PCA showing the contribution of quantitative traits among 220 lentil genotypes grown in 2017-18.

Further, s~~S~~cattered diagram based on first two factors presented in Fig. 2 showed that the genotypes *viz*., 5638, 5583, 6124, 6052, 6010, 5684, 5979 and 6101 were present in the extreme upper right side of the scattered plot due to response towards HSW, DM and PS. The genotypes

6054, 5556, 23776 and 5650 were placed at an extreme upper left side with mixed performance of the genotypes for various traits. Such type of genotypes required further evaluation and characterization to explore full potential of the germplasm. Similarly, the genotypes 6122, 6084, 5689 and 5590 presented on the lower right side of the scattered plot were contributed through BY, SY, NP, NSP and HS.

Fig. 2. Scattered plot of PCA for lentil genotypes studied during 2017-18.

Moreover, b~~B~~ased on PCA during 2019, first five components had >1 Eigenvalue, whereas other PCs contributed low in cumulative variation (S5 Table). Contribution by SY, BY, and HSW were~~as~~ highest toward factor 1 while factor 2 was contributed more by CT, SP and HS. The separation of genotypes and their accumulation in the particular principal components indicated the performance and association towards yield. Resultantly, f~~F~~actor 1 showed 19.91% variability while factor 2 as 12.19% with an overall contribution of 32.10% (Fig. 3).

Fig. 3. PCA showing the contribution of quantitative traits among 220 lentil genotypes grown in 2018-19.

Furthermore, g~~G~~enotypes 5506, 5729, 5510, 5672, 5636, 5581, 5491 and 5483 at the upper right corner were grouped on the basis of HSW, BY and SY indicating ~~promising~~ elite genotypes while in the lower right corner genotypes 5472, 5512, 5479, 5486, 5650, 5600 and 5531 reflected promising results for PH, NP and HS. Therefore, increase in height would result in higher number of seeds per pods which ultimately would contribute towards high yield. Similarly, at the upper left side of the scattered plot, genotypes 6080, 6043, 5653, 6043, 6002, 6060 and 5677 showed variability and high response to CT, NSP and PS which could be helpful in selection for genotypes with desirable CT. While on the other hand, at lower left side

Formatted: Indent: First line: 0.5"

genotypes 23787, 24787, 23779, 5742 and 5712 were placed with the highest variability for DM and LPH which supports to select such genotypes that showed a positive ~~correlation~~ association between both of these traits (Fig. 4). Likewise, l~~L~~ower pod height helps in early or timely maturity of the crop, as stature of plant helps in gathering more light and less shade, probably high metabolism and access to resources.

Fig. 4. Scattered plot of PCA for lentil genotypes grown in 2019

Hierarchical cluster analysis

Cluster analysis based on quantitative data in 2017-18 is presented in Fig. 5 which had divided the genotypes into 4 main clusters based on 50% linkage distance (LD) of 14.5. Cluster 1 consisted of 106 diverse genotypes including 6015 originated from USA followed by cluster 2 which contains 85 genotypes including 5698 originated from Sialkot, Pakistan with distinct position. Cluster 3 consisted of 20 genotypes where 3 are representing Pakistan found distinct (5689 Sialkot, 5687 and 6084 from Narowal). Cluster 4 composed of only 9 genotypes with unique performance for SY, BY, PS, NSP, CT and HS. Selection could be made for clusters having unique performance with desired traits, hence suggested to use for future lentil breeding.

The hierarchical cluster pattern obtained for 2nd year 2018-19 indicated 7 main clusters at 50% linkage distance of 8.4 (Fig. 6). Cluster 1 consisted of 37 genotypes, cluster 2 (115), cluster 3 (12~~) ,~~), while cluster 4 and 5 were ~~of 1~~of 1 (24783) and 4 genotypes (6084, 6058, 6062 and 6122), respectively. Further, c~~C~~luster 6 consisted of 49 genotypes and cluster 7 only 2 genotypes. Best performing genotypes were represented in C3 during 2017-18 and C5 during 2018-19 due to their performance as SY, BY, PS, NSP, CT~~,~~ and HS. Cluster 2 represents HSW > 1.19 producing genotypes during 2017-18, on the other hand, those genotypes which produced HSW

> 1.19 during 2018-19 were grouped into cluster 3. Similarly, c~~C~~luster 2 was also consisted of those genotypes which had PH > 55.1 and DM > 170 in both years. While c~~C~~luster 1 consisted of genotypes having SY > 40 or near to 40 g during both years.

Fig. 5. Hierarchical dendrogram showing the relationship of lentil genotypes for ~~agronomic~~ quantitative traits studied during 2017-18.

Fig. 6. Hierarchical dendrogram showing the relationship of lentil genotypes for quantitative~~agronomic~~ traits studied during 2018-19.

Structural equational model (SEM)

SEM was constructed for SY based on covariance studies as shown in Table 5. SY was taken as a dependent variable whereas other traits such as HSW, BY, PH, LPH, PS, DM, NP and NSP were taken as independent variables (Fig. 7). Contribution of independent variables like HSW (P values 0.10), BY (P values - 0.09), PH (P values 0.00), LPH (P values 0.01), NSP (P values 0.81), PS (P values 0.00), DM (P values 0.00) and NP (P values 0.04) observed significant at level < 1. This was further verified by the lowest error variance of multiple linear regressions. The value of coefficient of determination (R^2 ~~-~~= 0.64) of these multiple linear relationships supports this hypothesis that real yield contributors are given in the equation and these individual traits are contributing in their percent capacity share as shown in figure.

Fig. 7. Structural equations of model for seed yield (SY) during 2017-18 and 2018-19

Table 5. Structural equationals ~~of~~ model representing regression for agronomic traits studied during 2017-18 and 2018-19.

SY	HSW	BY	PH	LPH	NSP	PS	DM	NP
Z-values	2.12	0.27	0.02	0.43	3.69	5.43	-0.04	2.21
P-values	0.10	0.09	0.00	0.01	0.81	0.0	0.0	0.04

SY = 2.16*HSW + 0.27*BY + 0.025*PH + 0.0.43*LPH + 1.826*NSP + 6.34*PS - 0.039*DM + 2.298*NP

Formatted: Font: 10 pt

SY, seed yield; BY, biological yield; PH, plant height; LPH, lower pod height; PS, pod size; DM, days to maturity; NP, number of pods per peduncle; NSP, number of seeds per pod.

Heritability analysis

Phenotypic coefficient of variance founded to be greater than the genotypic coefficient of variance during 2017-18 and 2018-19 for all the traits under study (Table 6). High heritability was observed in 220 lentil genotypes along with two check verities. Seeds per pod showed low heritability value 0.46 during 2017-18 while considerably high during 2018-19 as 0.88. Other traits showed high heritability such as 0.99 for BY, PH, LPH~~,~~ and DM during 2017-18 while during 2018-19 LPH and DM showed the same heritability value. The high heritability was observed for BY (0.99) along with high genetic advance as a percentage of mean 79.71 for 2017-18 while for the subsequent year LPH was 27.94. It indicates predominance of additive gene action for these traits under prevailing conditions. Further, high genetic advance along high heritability estimates provides ample opportunity for most suitable selection of traits for future application through crossing or hybridization for varietal development.

359

Table 6. Estimation of heritability and genetic advance for the years 2017-18 and 2018-19

Traits/Years	Environmental Variance		Genotypic Variance		Phenotypic Variance (σ_p)		Environmental Coefficient of Variance		Genotypic Coefficient of Variance		Phenotypic Coefficient of Variance		Heritability (Broad Sense) (h^2)		Genetic Advance		Genetic Advance as percentage of mean	
	2018	2019	2018	2019	2018	2019	2018	2019	2018	2019	2018	2019	2018	2019	2018	2019	2018	2019
SY	6.21	3.75	206	163.9	212.9	167.72	8.23	7.69	47.47	50.84	48.18	51.41	0.97	0.97	29.18	26.08	96.36	103.55
HSW	0.01	0.00	0.11	0.07	0.12	0.08	7.64	5.66	19.3	15.83	20.80	16.81	0.86	0.88	0.63	0.51	37.06	30.71
BY	16.9	33.09	1978	1050.9	1995	1084.	3.59	6.11	38.85	34.43	39.02	34.97	0.99	0.96	91.24	65.75	79.71	69.85
PH	0.46	0.61	47.04	48.10	47.5	48.72	1.51	1.61	15.26	14.23	15.34	14.32	0.99	0.98	14.06	14.19	31.29	29.13
LPH	0.02	0.02	5.52	4.16	5.54	4.18	1.01	1.10	15.87	13.61	15.90	13.65	0.99	0.99	4.83	4.18	32.63	27.94
PS	0.00	0.00	0.01	0.01	0.01	0.01	3.07	4.88	20.4	21.92	20.70	21.92	0.97	0.95	0.17	0.16	41.48	44.24
SP	0.01	0.00	0.01	0.07	0.02	0.08	5.69	3.21	5.34	13.66	7.81	14.04	0.46	0.94	0.15	0.56	7.52	27.40
DM	0.13	0.13	25.60	25.60	25.73	25.74	0.19	0.19	2.73	2.73	2.74	2.74	0.99	0.99	10.39	10.39	5.62	5.62
HS	0.00	0.01	0.37	0.36	0.38	0.38	25.88	30.52	163.9	163.12	165.9	165.9	0.97	0.96	1.24	1.23	333.5	330.32
NP	0.01	0.00	0.04	0.02	0.05	0.03	5.50	2.51	9.69	7.90	11.15	8.29	0.75	0.90	0.35	0.31	17.36	15.53

SY, seed yield; HSW, hundred seed weight; BY, biological yield; PH, plant height; LPH, lower pod height; PS, pod size; NSP, number of seeds per pod; DM, days to maturity; CT, cooking time; HS, hard seed; NP, number of pods per peduncle.

366

21

Discussion

Selection of the promising genotypes is the ultimate objective of scientists to develop cultivars for farmers' field. Genetic diversity has been used to characterize the genotypes in a range of plant species including lentil [30]. Wide variation was observed in the present study in lentil including SY from 3 to 85 g by 6084, which produced highest SY for both years [16]. It is always to be desirable by breeders to choose high yielding genotypes exhibit highest yield potential to develop varieties or to carry out multi-location trials for varying environments [31]. High BY increases the photosynthetic area which in turn enhances food quality and SY. In the present study, 6122, 5689, 5730 and 6037 exhibited the best BY during 2017-18, likewise genotypes 6042, 6087 and 5689 were best for 2018-19. Improvement in BY at the vegetative growth stage before flowering leads to further increase in N-assimilation and thereby in SY [24, 33-35]. Nitrogen assimilation transfer atmospheric nitrogen which is available in the environment into amino acids, therefore it is one of the natural property of legumes which make them more proteinaceous food.

Overall performance was better in the first year as compared to subsequent year that is more likely to be attributed to temperature fluctuation during both of the years, from 21°C in March to 26°C in April during 2017-18 and 19°C in March to 24°C in April during 2018-19. It is presumed that low temperature delayed flowering and pod filling process, however lentil requires low temperature during vegetative stage, while the reproductive stage needs high temperatures [36]. The optimum temperature for vegetative stage of lentil ranged from 6.1-27.3°C while 17.4-31.5°C during reproductive stage [37]. Likewise, plant height (PH) is a major yield contributing trait which supports sufficient vegetative growth and branching, ultimately develops more pods that means increased SY [7, 38].

High variation in native as well as exotic germplasm was found for both years. There were additionally several environmental factors which influence crop performance as water scarcity affects productivity of legumes at growth stage, perhaps drought during reproductive and grain filling stages (terminal drought) are more critical and usually result in a significant loss of SY and of course negative effects on yield contributing traits too [39, 40]. In the present studies, genotypes 5698 (Sialkot, Pakistan) and 6015 (USA) were found as early maturing genotypes in 2017-18 while 24783 (unknown) and 5561 (Sanghar, Pakistan) for 2018-19 with 161-172 days. Current findings are more appealing as compared to previous reports of 168-190 days [16]. These early maturing genotypes are suggested for further crop improvement program as well against drought hit areas during cold season after adopting breeding strategy for lentils [41].

It is evident that SY is directly proportional to NSP, NP ranged from 2-3 in 2017-18 and 1-3 in 2018-19, selection based on stable genotypes for NP would proof baseline material for starting multi-location trials under different environmental conditions to develop new varieties [17]. Similarly, HSW is considered as an indicator of seed quality and a measure of grain bulk density. In this study HSW varied between the years with 3.65 g in 2017-18 and 3.28 g in 2018-19, showed wide variation during both years. Similar type of studies were also conducted for wide range of crops including lentil not only in Pakistan and Turkey but also in other countries of the world with respect to agro-morphological characterization [3, 7, 11, 14-18, 39, 42].

Further, cooking time (CT) is a favorite trait for consumers in case of legume crops, it saves energy. CT of 10 minutes was observed in genotypes 6074 and 5745. It is not only saves energy and time but also it develops adequate flavor, texture and improves protein digestibility in lentils [8]. More CT causes loss of proteins, vitamins, minerals and other secondary metabolites

[9]. Overall, the genotype 6122 performed the best out of 220 genotypes, therefore, suggested further for trials and breeding programs to develop as a new variety for commercial purposes.

Inheritance studies of qualitative traits like GCT and GPT evidenced that GCT is determined by two independent loci [43]. The dominant allele at one locus (Ggc) determines grey seed coat color and dominant allele at second locus (Tgc) determines tan seed coat color. Two dominant alleles (Ggc Tgc) interact to produce a brown seed GCT whereas double recessive (ggc tgc) as green color. Seed coat pattern is determined by a series of five alleles at another locus [41]. Moreover, pod pigmentation was absent in 69% of genotypes with low PD and PSh. Low PD and PSh are considered most favorite traits from a farmer’s perspective because it reduces grain loss during harvesting. Further, slight LP with a small leaf and prominent tendril were most frequently found. While dense LP was found in 15% of genotypes. Presence of dense LP (leaf hairs) reduces absorbance of photosynthetically active radiation (400 to 700 nm). Reduction in photosynthetic rate is caused by decreased light absorption rather than decreased carbon dioxide conductance through boundary layer [17]. It has been also reported that light LP with a small leaf and prominent tendril exhibit high resistance to lodging. Likewise furthermore Additionally, seedling stem pigmentation was present in most varieties. All of the genotypes exhibited white blue line flowers [42].

Improvement in a target character can be achieved by indirect selections which are more heritable and easier to select [43]. Furthermore, this strategy of selection requires understanding interrelationships among characters and with target traitscharacters. A significant correlation between SY and most of the quantitative traits were documented in both years. Positive combinations can be helpful in future hybridization programs to obtain highest outcomes. -It is recognized that sSeed yield per hectare is mainly associated with an increase in the percentage of

seed germination, NP and seed index, in contrary, while prolonged flowering and delayed maturity in lentil crop reduces SY [44, 45]. Therefore, someone should be very careful about sowing time and its stage. The number of pods per peduncle had a significant positive correlation with SY and yield contributing traits like BY and HSW [16, 46].

In PCA contribution of variables toward traits revealed that SY, BY and PH had highest values in factor 1 which represent high variability among these traits whereas in factor 2 highest values were recorded in HSW, PH, and HS which are recommended for further selection and characterization. Similar results were observed in lentil collected from Pakistan and Turkey in the past [16, 17]. Scattered diagrams reflected high variation as spreads genotypes where PC showed that genotypes with better performance for SY, BY, PS, HSW, PH, and HS displayed at extreme position. Previous studies showed that > 80 % of genetic variation is contributed through first two components [24, 47-48]. It was revealed that component factor 11 indicated high potential for both SY and BY, therefore this component is also suggested for further usestudies. Similarly, factor 4 can be shortlisted for DM as well factor 7 for HSW and factor 8 for PS. Genotypes with best CT placed in factor 6 during 2017-18 and factor 2 during 2018-19. Correlation matrix for DM showed negative association with CT and supported by the fact that time of maturity always influenced by variation in environmental factors. Therefore, it is assumed that probably genotypes with best CT placed in different components during both of the years. Individual components can also be selected for NP, LPH, and PH which were placed in factors 3, 4, and 5, respectively. It can be derived that PCs which showed consistent performance in both years would be more beneficial to cater genotypes potential.

Hierarchical cluster analysis revealed that genotype 6015 (USA) and 5698 (Sialkot, Pakistan) showed an outlier from all other clusters in 2017-18 which indicated that these

genotypes performed well for DM. Genotypes 5664 (Sialkot, Pakistan) and 6074 (Bhawalnager, Pakistan) showed outliers from other clusters in 2018-19, resultantly~~,~~ placed in cluster 7. These genotypes performed best for the maximum number of quantitative traits as well as SY. It was also recorded that Syrian germplasm grouped into cluster 2 in 2018-19 while collections representing USA and Pakistan grouped into different clusters. On the other hand, genotypes of Syria, USA~~,~~ and Pakistan during 2017-18 were grouped in C2 clusters that might be due to common ancestry. It is assumed that distribution of genotypes during both of the years in separate clusters was due to environmental conditions especially temperature at experimental site or could be genetic material that carries genes of interest which can play significant role in lentil breeding program as well as in the improvement of these traits through selection [49-51]. Inter-cluster distance between genotypes of any two clusters indicated that these would be more genetically divergent [52, 53].

Another statistical approach used as structural equation model provides illustration of association among multifactorial traits which drives that SY is dependent on other traits in a casual way and path of the characters are towards SY. In the current findings, SEM showed significant level <_1 contribution of independent variables like LPH, PS, DM, HS, NP, NSP toward SY [16]. The high value of phenotypic coefficient of variance as compared to genotypic coefficient of variance indicated~~s~~ influence of environment on traits as well as it specified~~s~~ the possibility for selection of traits. Result showed that genotypic coefficient of variance was found to be less than the phenotypic coefficient of variance that might be due to environmental factors influenced the expression of the phenotypes. Most of the agro-morphological traits had fairly higher heritability [18]. A trait with high heritability indicates that these have more potential for utilizing available genetic variability as compared to those showing low values. Traits that

showed low level of heritability need further consideration like NSP. The high heritability value along with high genetic advance for SY, BY~~,~~ and LPH signifies the selection of these traits for further crop improvement [54]. It is assumed that a high genetic advance under selection could be achieved when characters are highly heritable and stable due to additive gene action. Traits with low heritability can be improved through selection using heterosis breeding [55, 56]. Values for phenotypic variance inferred those environmental factors played significant role as compared to genotypic variance for heritable quantitative traits [57]. Therefore, characters with high heritability are more important to gather maximum variation for starting varietal development program.

Conclusion

Genotype 6122 originated from Sheikhupura, Pakistan performed best for several traits during both years, hence recommended for farmers' field cultivation after conducting necessary trials. Yield per plant was recorded ~~accounted~~ as 8.05 g and ~~which~~ ultimately provides estimates of yield tons/hectare as 2.68, while ~~as~~ check variety Markaz-09 have~~d~~ yield potential of 3.2 tons/hectare, advocates high future prospective for current lentil germplasm. Genotypes representing Pakistan and Syria exhibited best values for BY~~biological yield~~ which ~~that~~ possess ~~have~~ potential to be used as fodder or forage purposes, too. Present investigation reported DM ranged from 161-198 days during both of the years while earlier reports showed more time for maturity. Short duration genotypes (5698, 6015, 5667, 5748, 5555, 6017, 5700, 5571, 5580, 5981~~,~~ matured in < 178 days should be further tested for selecting early ~~crop~~ maturity lines~~improvement~~. ~~PCA suggests selection of individual traits that carry high variability due to occurrence and gathering genotypes at the unique positions through scattered diagrams.~~

Moreover, hierarchical cluster analysis advocates geographical distribution of germplasm that is independent of origin and gathered genotypes of different origin into same cluster. The high GCV along with heritability and genetic advance depicted wide variation for selection with improving yield traits in lentil cultivars. Ten genotypes including 5664, 5687, 6084, 6062, 6122, 6058, 6087, 5689, 6042, and 6074 were selected based on high yield and recommended for future crop improvement programs.

Declaration

Not applicable

Funding

Not applicable

Conflicts of interest/competing interests

Authors declare no competing interests

Availability of data and material

All of the relevant data is already included

Authors' contributions

Conceptualization: Muhammad Sajjad Iqbal, Abdul Ghafoor, Data curation: Syed Atiq Hussain, Formal analysis: Saba Munir, Ayesha Tahir[2], Methodology: Muhammad Akbar[1], Noshia Arshad[1]. Resources: Muhammad Ahson Khan[3], Muhammad Kashif Ilyas[3]. Software:

Muhammad Azhar Ali[1], Syed Atiq Hussain. Supervision: Muhammad Sajjad Iqbal. Validation: Hajra Masood[1], Tahira Ahmad[1], Nazra Shaheen[1], Writing – original draft: Syed Atiq Hussain, Muhammad Sajjad Iqbal. Writing – review & editing: Muhammad Sajjad Iqbal, and Abdul Ghafoor.

Consent to participate

Not applicable

Consent for publication

All authors are willing to publish this manuscript in this journal

References

1. Ates D, Aldemir S, Yagmur B, Kahraman A, Ozkan H, Vandenberg A, et al. QTL mapping of genome regions controlling manganese uptake in lentil seed. G3. 2018; 8: 1409-1416.
2. Ates D. Genetic diversity in lentil landraces revealed by diversity array technology (DArT). Turkish J. Field Crops. 2019; 24: 252-260.
3. Scippa GS, Trupiano D, Rocco M, Viscosi V, Di Michele M, Dandrea A, et al. An integrated approach to characterization of two autochthonous lentil (*Lens culinaris*) landraces of Molise (~~south~~South-~~central~~ Central Italy). Heredity. 2008; 101: 136-144.
4. Thavarajah P. Evaluation of chickpea (*Cicer arietinum* L.) micronutrient composition: Biofortification opportunities to combat global micronutrient malnutrition. Food Res. Intern. 2012; 49; 99-104.

5. Mbasani MJ, Briache FZ, Ennami M, Gaboun F, Benbrahim N, Triqui ZEA, et al. Resistance of Moroccan lentil genotypes to *Orobanche crenata* infestation. J. Crop Improve. 2019; 33: 306-326.
6. Pandey S, Bhatore A. Genetic diversity analysis for quantitative traits in indigenous genotypes of lentil in Madhya Pradesh. J. Pharmacog. Phytochem. 2018; **7**, 279-283.
7. Gaad D, Laouar M, Abdelguerfi A, Gaboun F. Collection and agro-morphological characterization of Algerian accessions of lentil (*Lens culinaris*). Biodiversitas J. Biol. Divers. 2018; 19: 183-193.
8. Ramírez OAM, Moreno RR, Camara MF. Mineral and trace element content in legumes (lentils, chickpeas and beans): Bioaccesibility and probabilistic assessment of the dietary intake. J. Food Composition and Analysis, 2018; 73: 17-28.
9. Shi L, Arntfield SD, Nickerson M. Changes in levels of phytic acid, lectins and oxalates during soaking and cooking of Canadian pulses. Food Res.~~earch~~ Intern.~~ational.~~ 2018; 107:660-8.
10. FAOSTAT. http://www.fao.org/faostat/en/#data. 2019; Accessed 26 March 2019.
11. Noor R, Khan SM, Ahmad F, Hussain M, Abd-Allah EF, Alqarawi AA, et al. Morpho-agronomic characterization study of *Lens culinaris* genotypes under salt marsh habitat in Swat, Pakistan. Saudi J. Biol. Sci. 2017; 24: 1639-1645.
12. Khan A, Ihsan M, Nisar M, Hazrat A, Ali M, Ul-Haq R, et al. Evaluation of genetic diversity in barley landraces through agromorphological and Biochemical Characterization. Sarhad J. Agri. 2011; 37: 984-992.

13. Ullah J, Hussain SA, Nisar M, Khan W, Khan U, Khan J et al. Biochemical characterization of lentil genotypes for genetic diversity. Plant Cell Biotech. Mol. Biol. 2016; 17: 7-13.

14. Gupta R, Begum SN, Islam MM, Alam MS. Characterization of lentil (*Lens culinaris* M.) genotypes through phenotypic marker. J. Bangladesh Agri. Uni. 2012; 10: 197-204.

15. Cristobal MD, Pando V, Herrero B. Morphological characterization of lentil (*Lens culinaris* Medik.) landraces from Castilla y León, Spain. Pakistan. J. Bot. 2014; 46: 1373-1380.

16. Bilal M, Jamil M, Ali A, Ilyas MK, Ghafoor A. Multivariate analysis of genetic diversity in lentil (*Lens culinaris*) germplasm. Fres. Environ. Bull. 2018;~~.~~ 27: 2151-2159.

17. Mohammed NA, Refay YA, Migdadi HM, AL-Somain BH, Muharram AA, Al-Selwey, et al. Agro~~-~~morphological characterization of lentil genotypes in dry environments. Intern. J. Agri. 2019; 22: 1320-1330.

18. Naoura G, Sawadogo N, Atchozou EA, Emendack Y, Hassan MA, Reoungal. Assessment of agro~~-~~morphological variability of dry ~~-~~season sorghum cultivars in Chad as novel sources of drought tolerance. Scientific Rep. 2019; 9: 1-12.

19. Santos CS, Silva B, Valente LMP, Gruber S, Vasconcelos MW. The effect of sprouting in lentil (*Lens culinaris*) nutritional and microbiological profile. Foods. 2020; 9: 400.

20. Iqbal MS, Akbar M. Phenotypic diversity analysis and performance of elite lines of *Brassica napus* L. Bangladesh J. Bot. 2021; 50(1): 1-6~~, 2021~~.

21. Nisar M, Ghafoor A, Ahmad H, Khan MR, Qureshi AS, Ali H, Islam M. Evaluation of genetic diversity of pea germplasm through phenotypic trait analysis. Pakistan. J. Bot.~~,~~ 2008; 40(5): 2081-2086~~, 2008~~.

22. Ghafoor A, Arshad M. Multivariate analyses for quantitative traits to determine genetic diversity of blackgram [*Vigna mungo* (L.) Hepper] germplasm. Pakistan. J. Bot., 2008; 40(6): 2307-2313, 2008.

23. Tucak M, Popović S, Cupic T, Šimić G, Gantner R, Meglic V. Evaluation of alfalfa germplasm collection by multivariate analysis based on phenotypic traits. Romanian Agri. Res. 2009; 26: 47-52(26), 2009.

24. Iqbal MS, Akbar M, Akhtar S, Fatima S, Ghafoor A. Multivariate analysis and its application for characterizing cowpea landraces. Legume Res. 2021; 44 (1): 1-7. 2021.

25. Iqbal Z, Arshad MG, Ashraf M, Mahmood T, Waheed A. Evaluation of soybean [*Glycine Max* (L.) Merrill] germplasm for some important morphological traits using multivariate analysis. Pakistan. J. Bot. 2008; 40(6):2323-2328., 2008.

26. Roy S, Islam MA, Sarker A, Malek MA, Rafii MY, Ismail MR. Determination of genetic diversity in lentil germplasm based on quantitative traits. AJCS. 2013; 7(1):14-21, 2013.

27. Iqbal MS, Qureshi AS, Ghafoor A, Qayyum A. Identification of superior genotypes based on morphological, physiological and agronomic traits in local and exotic cowpea germplasm. Pakistan. J. Bot. 2003; 35(1):69-77.2003.

28. Singh RK, Choudhury BD. Biometrical method in quantitative genetic analysis. Kalyani Publishers, Ludhiana, New Delhi, 1985; , pp. 54-57.

29. Popat R, Patel R, Parmer D. Variability: Genetic variability analysis for plant breeding research. R package version. http://cran.rproject.org/web/packages/variability/index.html. 2020. Accessed 07 September 2021.

30. Hussain N, Yaqoob M, Rashid A. Genetic competition among lentil (*Lens culinaris*) candidate lines for yield and yield components under rainfed conditions. J. Agri. Res. 2014; 52.

31. Iqbal MS, Ghafoor A, Abbasi FM, Qureshi AS, Ahmad H. Study of nutritional characteristics, mineral nutrients and agro-biodiversity in black cumin (*Nigella sativa* L.) genotypes from Pakistan. Afri. J. Biotech. 2011; 10: 14757-14766.

32. Paliya S, Saxena A, Tikle AN, Singh M, Tilwari A. Genetic divergence and character association of seed yield and component traits of lentil (*Lens culinaris* M.). Adv. Biores. 2015; 6: 53-59.

33. Bhartiya A, Aditya JP, Singh S. Assessment of variability for agro-morphological traits in elite lentil (*Lens culinaris*) lines using multivariate analysis. Indian J. Agri. Res. 2015; 49: 539-543.

34. Ghanem, M. E., Marrou, H., Soltani, A., Kumar, S, Sinclair, TR. Lentil variation in phenology and yield evaluated with a model. Agron. J. 2015; 107: 1967-1977.

35. Lee YJ, Sung JK, Lee SB, Lim JE, Song YS, Lee DB. Yield and free sugar contents of burdock (*Arctium lappa* L.) depending on nitrogen levels. Korean J. Soil Sci. Fertilizer. 2016; 49: 677-681.

36. Roy S, Islam MA, Sarker A, Ismail MR, Rafii MY, Mondal MMA, Malek MA. Morphological characterization of lentil accessions: qualitative characters. Bangladesh J. Bot. 2012; 41: 187-190.

37. Sita K, Sehgal A, Kumar J, Kumar S, Singh S, Siddique K H, Nayyar H. Identification of high temperature tolerant lentil (*Lens culinaris* Medik.) genotypes through leaf and pollen traits. Front. Plant Sci. 2017; 8: 744.

38. Kumar S, Hamwieh A, Manickavelu A, Kumar J, Sharma TR, Baum M. Advances in lentil genomics. In: *Legumes in Omic*. Springer Science + Business Media, New York, USA. 2014; 111-130.

39. Ma Y, Dias MC, Freitas H. Drought and salinity stress responses and microbe-induced tolerance in plants. Frontiers in Plant Sci. 2020; 11: 1750.

40. Pushpavalli R, Zaman-Allah M, Turner NC, Baddam R, Rao MV, Vadez V. Higher flower and seed number leads to higher yield under water stress conditions imposed during reproduction in chickpea. Functional Plant Biol. 2014; 42: 162-174.

41. Nadeem M, Li J, Yahya M, Sher A, Ma C, Wang X, et al. Research progress and perspective on drought stress in legumes: a review. Intern. J. Mole Sci. 2019; 20: 2541.

42. Santos CS, Carbas B, Castanho A, Bronze MR, Serrano C, Vasconcelos MW, Brites C. Relationship between seed traits and pasting and cooking behavior in a pulse germplasm collection. Crop and Pasture Sci. 2018; 69; 892-903.

43. Mirali M, Purves RW, Stonehouse R, Song R, Bett K, Vandenberg A. ~~(2016).~~ Genetics and biochemistry of zero-tannin lentils. PLoS One. (2016); 11(10), e0164624.

44. Dixit GP, Katiyar PK, Singh BB. Characterization of lentil (*Lens culinaris* Medik.) varieties based on morphological traits. J. Food Leg. 2011; 24: 194-197.

45. Dugassa A, Legesse H, Geleta N. Genetic variability, yield and yield associations of lentil (*Lens culinaris* Medic.) genotypes grown at Gitilo Najo, Western Ethiopia. Sci. Tech. Arts Res. J. 2015; 3: 10-18.

46. Depar MS, Soomro NA, Chandio AS, Samoon HA, Meghwar BL, Pathan AK, Kalroo MW. Evaluation of morpho-yield traits and their correlation with seed yield in lentil (*Lens culinaris*) genotypes. Sci. Intern. 2016; 28.

47. Abo-Hegazy SRE, Selim T, El-Emam EAA. Correlation and path coefficient analyses of yield and some yield components in lentil. Egyptian J. Plant Breed. 2012; 203: 1-14.

48. Shrestha J, Subedi S. Improving crop productivity through sustainable intensification. South Asian Res. J. Agri. Fish. 2019; 1: 8-11.

49. Afuape SO, Okocha PI, Njoku D. Multivariate assessment of the agro–morphological variability and yield components among sweet potato (*Ipomoea batatas* (L. Lam) landraces. African J. Plant Sci. 2011; 5: 123-132.

50. Benbrahim N, Taghouti M, Zouahri A, Gaboun F. On-farm conservation of Zaer lentil landrace in context of climate change and improved varieties competition. Univ. J. Agric. Res. 2017; 5: 27–38.

51. Idrissi O, Piergiovanni A, Toklu F, Houasli C, Udupa S, De Keyser E, De Riek J. Molecular variance and population structure of lentil (*Lens culinaris* Medik.) landraces from Mediterranean countries as revealed by simple sequence repeat DNA markers: Implications for conservation and use. Plant Genet. Resour. 2018; 16: 249-259.

52. Raza I, Masood MA, Abid S, Farooq M A, Mustafa R. Exploring relationship among quantitative traits of sugarcane varieties using principal component analysis. Sci. Tech. Develop. 2017; 36: 142-146.

53. Nath UK, Rani S, Paul MR, Alam MN, Horneburg B. Selection of superior lentil (*Lens esculenta* M.) genotypes by assessing character association and genetic diversity. The Sci. World J. 2014; 6.

54. Tyagi SD, Khan MH. Studies on genetic variability and interrelationship among different traits in Microsperma lentil (*Lens culinaris* Medik.). J. Agri Biotech Sustain. Devel. 2010; 2: 15.

55. Asghar MJ, Abbas G, Shah TM, Atta BM. Study of genetic diversity in some local and exotic lentil (*Lens culinaris* Medik) genotypes. Pakistan. J. Bot. 2010; 42: 2681-2690.
56. Bicer BT, Sakar D. Heritability of yield and its components in lentil (*Lens culinaris* Medik.). Bulgarian J. Agri. Sci. 2010; 16: 30-35.
57. Sarfraz Z, Shah MM, Iqbal MS. Genetic variability, heritability and genetic advance for agronomic traits among genome donor wheat genotypes. J. Agric. Res. 2016; 54: 15-20.

Supporting information

S1 Table. List of lentil genotypes studied during 2017-18 and 2018-19. Two varieties Markaz 2009 and Punjab 2009 were used as check.

S2 Table. Mean±SE for quantitative traits of 5% best performing lentil genotypes during 2017-18.

S3 Table. Mean±SE for quantitative traits of 5% best performing lentil genotypes during 2018-19.

S4 Table. Principal component analysis for quantitative traits of lentil studied during 2017-18.

S5 Table. Principal Component Analysis for quantitative traits studied during 2018-19.

S1 Fig. Average temperature during lentil crop season October to April 2017-18 and 2018-19 at the experimental station

Table 6. Estimation of heritability and genetic advance for the years 2017-18 and 2018-19

Traits/Years	Environmental Variance		Genotypic Variance		Phenotypic Variance (σ_p)		Environmental Coefficient of Variance		Genotypic Coefficient of Variance		Phenotypic Coefficient of Variance		Heritability (Broad Sense) (h^2)		Genetic Advance		Genetic Advance as percentage of mean	
	2018	2019	2018	2019	2018	2019	2018	2019	2018	2019	2018	2019	2018	2019	2018	2019	2018	2019
SY	6.21	3.75	206	163.9	212.9	167.72	8.23	7.69	47.47	50.84	48.18	51.41	0.97	0.97	29.18	26.08	96.36	103.55
HSW	0.01	0.00	0.11	0.07	0.12	0.08	7.64	5.66	19.3	15.83	20.80	16.81	0.86	0.88	0.63	0.51	37.06	30.71
BY	16.9	33.09	1978	1050.9	1995	1084.	3.59	6.11	38.85	34.43	39.02	34.97	0.99	0.96	91.24	65.75	79.71	69.85
PH	0.46	0.61	47.04	48.10	47.5	48.72	1.51	1.61	15.26	14.23	15.34	14.32	0.99	0.98	14.06	14.19	31.29	29.13
LPH	0.02	0.02	5.52	4.16	5.54	4.18	1.01	1.10	15.87	13.61	15.90	13.65	0.99	0.99	4.83	4.18	32.63	27.94
PS	0.00	0.00	0.01	0.01	0.01	0.01	3.07	4.88	20.4	21.92	20.70	21.92	0.97	0.95	0.17	0.16	41.48	44.24
SP	0.01	0.00	0.01	0.07	0.02	0.08	5.69	3.21	5.34	13.66	7.81	14.04	0.46	0.94	0.15	0.56	7.52	27.40
DM	0.13	0.13	25.60	25.60	25.73	25.74	0.19	0.19	2.73	2.73	2.74	2.74	0.99	0.99	10.39	10.39	5.62	5.62
HS	0.00	0.01	0.37	0.36	0.38	0.38	25.88	30.52	163.9	163.12	165.9	165.9	0.97	0.96	1.24	1.23	333.5	330.32
NP	0.01	0.00	0.04	0.02	0.05	0.03	5.50	2.51	9.69	7.90	11.15	8.29	0.75	0.90	0.35	0.31	17.36	15.53

SY, seed yield; HSW, hundred seed weight; BY, biological yield; PH, plant height; LPH, lower pod height; PS, pod size; NSP, number of seeds per pod; DM, days to maturity; CT, cooking time; HS, hard seed; NP, number of pods per peduncle.

Formatted: Width: 8.5", Height: 11"

Discussion

Formatted: Font: 18 pt

Formatted: Indent: First line: 0"

Selection of the promising genotypes is the ultimate objective of scientists to develop cultivars for farmers' field. Genetic diversity has been used to characterize the genotypes in a range of plant species including lentil [30]. Wide variation was observed in the present study on lentil for SY from 3 to 85 g by 6084, which produced highest SY for both years [16]. It is always to be desirable by breeders to choose high yielding genotypes exhibit highest yield potential to develop varieties or to carry out multi location trials for varying environments [31]. High biological yield increases the photosynthetic area which in return enhances food quality and SY. In the present study, 6122, 5689, 5730 and 6037 exhibited the best BY during 2017-18, whereas genotypes 6042, 6087 and 5689 were best for 2018-19. Improvement in BY at the vegetative growth stage before flowering leads to further increase in N-assimilation and thereby in SY [24, 33-35]. Nitrogen assimilation transfer atmospheric nitrogen which is available in the environment into amino acids, therefore it is one of the natural property of legumes which make them more proteinaceous food.

Overall performance was better in the first year as compared to subsequent year that is more likely to be attributed to temperature fluctuation during two years, from 21°C in March to 26°C in April during 2017-18 and 19°C in March to 24°C in April during 2018-19. It is presumed that low temperature delayed flowering and pod filling process, however lentil requires low temperature during vegetative stage, while the reproductive stage needs high temperatures [36]. The optimum temperature for vegetative stage of lentil ranged from 6.1- 27.3°C while 17.4-31.5°C during reproductive stage [37]. Likewise, plant height (PH) is a major yield contributing trait which supports sufficient vegetative growth and branching, which ultimately develops more pods that means increased SY [7, 38].

High variation in native as well as exotic germplasm was found for both years. There were additionally several environmental factors which influence crop performance as water scarcity affects productivity of legumes at growth stage, perhaps drought during reproductive and grain filling stages (terminal drought) are more critical and usually result in a significant loss of SY and of course negative effects on yield contributing traits too [39, 40]. In the present studies, genotypes 5698 (Sialkot, Pakistan) and 6015 (USA) were found as early maturing genotypes in 2017-18 while 24783 (unknown) and 5561 (Sanghar, Pakistan) for 2018-19 with 161-172 days. Current findings are more appealing as compared to previous report of 168-190 days [16]. These early maturing genotypes are suggested for further crop improvement program as well against drought hit areas during cold season after adopting breeding strategy for lentils [41].

It is evident that SY is directly proportional to NSP, NP ranged from 2-3 in 2017-18 and 1-3 in 2018-19, selection based on stable genotypes for NP would proof baseline material for starting multi-location trials under different environmental conditions to develop new varieties [17]. Similarly, HSW is considered as an indicator of seed quality and is a measure of grain bulk density. Present findings elaborated the highest HSW was 3.65 g in 2017-18 followed by 3.28 g in 2018-19, showed wide variation during both years. Similar type of studies were also conducted for wide range of crops including lentil not only in Pakistan and Turkey but also in other countries of the world with respect to agro-morphological characterization [3, 7, 11, 14-18, 39, 42].

Cooking time (CT) is a favorite trait for consumers in case of legume crops, it saves energy. CT of 10 minutes was observed in genotypes 6074 and 5745. It is not only saves energy and time but develops adequate flavor, texture and improves protein digestibility in lentils [8]. More CT causes loss of proteins, vitamins, minerals and other secondary metabolites [9]. Overall, the

genotype 6122 performed the best out of 220 genotypes, therefore, suggested further for trials and breeding programs to develop as a new variety.

Inheritance studies of qualitative traits like GCT and GPT evidenced that GCT is determined by two independent loci [43]. The dominant allele at one locus (Ggc) determines grey seed coat color and dominant allele at second locus (Tgc) determines tan seed coat color. Two dominant alleles (Ggc Tgc) interact to produce a brown seed GCT whereas double recessive (ggc tgc) as green color. Seed coat pattern is determined by a series of five alleles at another locus [41]. Pod pigmentation was absent in 69% of genotypes with low PD and PSh. Low PD and PSh are considered most favorite traits from a farmer's perspective because it reduces grain loss during harvesting. Further, slight LP with a small leaf and prominent tendril were most frequently found. While dense LP was found in 15% of genotypes. Presence of dense LP (leaf hairs) reduces absorbance of photosynthetically active radiation (400 to 700 nanometers). Reduction in photosynthetic rate is caused by decreased light absorption rather than decreased carbon dioxide conductance through boundary layer [17]. It has been also reported that light LP with a small leaf and prominent tendril exhibited high resistance to lodging. Furthermore, seedling stem pigmentation was present in most varieties. All of the genotypes exhibited white blue line flowers [42].

Improvement in a target character can be achieved by indirect selections which are more heritable and easier to select [43]. Furthermore, this strategy of selection requires understanding interrelationships among characters and with target characters. A significant correlation between SY and most of the quantitative traits were documented in both years. Positive combinations can be helpful in future hybridization programs to obtain highest outcomes It is recognized that Seed yield per hectare is mainly associated with an increase in the percentage of seed germination, NP

and seed index, while prolonged flowering and delayed maturity in lentil crop reduces SY [44, 45]. Therefore, someone should be very careful about sowing time and its stage. The number of pods per peduncle had a significant positive correlation with SY and yield contributing traits like BY & HSW [16, 46].

In PCA contribution of variables toward traits revealed that SY, BY and PH had highest values in factor 1 which represent high variability among these traits whereas in factor 2 highest values were recorded in HSW, PH, and HS which are recommended for further selection and characterization. Similar type of findings was observed in lentil collected from Pakistan and Turkey in the past [16, 17]. Scattered diagrams reflected high variation as spreads genotypes where PC showed that genotypes with better performance for SY, BY, PS, HSW, PH, and HS displayed at extreme position. Previous studies showed that > 80% of genetic variation is contributed through first two components [24, 47-48]. It was revealed that component factor 11 indicated high potential for both SY and BY, therefore this component is also suggested for further studies. Similarly, factor 4 can be shortlisted for DM as well factor 7 for HSW and factor 8 for PS. Genotypes with best CT placed in factor 6 during 2017-18 and factor 2 during 2018-19. Correlation matrix for DM showed negative association with CT and supported by the fact that time of maturity always influenced by variation in environmental factors. Therefore, it is assumed that probably genotypes with best CT placed in different components during both of the years. Individual components can also be selected for NP, LPH, and PH which were placed in factors 3, 4, and 5, respectively. It can be derived that PCs which showed consistent performance in both years would be more beneficial to cater genotypes potential.

Hierarchical cluster analysis revealed that genotype 6015 (USA) and 5698 (Sialkot, Pakistan) showed an outlier from all other clusters in 2017-18 which indicated that these genotypes

performed well for DM. Genotypes 5664 (Sialkot, Pakistan) and 6074 (Bhawalnager, Pakistan) showed outliers from other clusters in 2018-19 resultantly, placed in cluster 7. These genotypes performed best for the maximum number of quantitative traits as well as SY. It was also recorded that Syrian germplasm grouped into cluster 2 in 2018-19 while collections representing USA and Pakistan grouped into different clusters. On the other hand, genotypes of Syria, USA, and Pakistan during 2017-18 were grouped in C2 clusters that might be due to common ancestry. It is assumed that distribution of genotypes during both of the years in separate clusters was due to environmental conditions especially temperature at experimental site or could be genetic material that carries gene of interest which can play significant role in lentil breeding program as well as in the improvement of these traits through selection [49-51]. Inter-cluster distance between genotypes of any two clusters indicated that would be more genetically divergent [52, 53].

Another statistical approach used as structural equation model provides illustration of association among multifactorial traits which drives that SY is dependent on other traits in a casual way and path of the characters are towards SY. In the current findings, SEM showed significant level <1 contribution of independent variables like LPH, PS, DM, HS, NP, NSP toward SY [16]. The high value of phenotypic coefficient of variance as compared to genotypic coefficient of variance indicates influence of environment on traits as well as it specifies the possibility for selection of traits. Result showed that genotypic coefficient of variance was found to be less than the phenotypic coefficient of variance that might be due to environmental factors influenced the expression of the phenotypes. Most of the agro-morphological traits had fairly higher heritability [18]. A trait with high heritability indicates that these have more potential for utilizing available genetic variability as compared to those showing low values. Traits that showed low level of

heritability need further consideration like NSP. The high heritability value along with high genetic advance for SY, BY, and LPH signifies the selection of these traits for further crop improvement [54]. It is assumed that a high genetic advance under selection could be achieved when characters are highly heritable and stable due to additive gene action. Traits with low heritability can be improved through selection using heterosis breeding [55, 56]. Values for phenotypic variance inferred those environmental factors played significant role as compared to genotypic variance for heritable quantitative traits [57]. Therefore, characters with high heritability are more important to gather maximum variation for starting varietal development program.

Conclusion

Formatted: Font: 18 pt

Genotype 6122 originated from Sheikhupura, Pakistan performed best for several traits during both years, hence recommended for farmers' field cultivation after conducting necessary trials. Yield per pant was accounted as 8.05 g which ultimately provides estimates of yield tons/hectare as 2.68 as check variety Markaz-09 had yield potential of 3.2 t/h, advocates high future prospective for current lentil germplasm. Genotypes representing Pakistan and Syria exhibited best values for biological yield that have potential to be used as fodder or forage purposes, too. Present investigation reported DM ranged from 161-198 days during both of the years while earlier reports showed more time for maturity. Short duration genotypes (5698, 6015, 5667, 5748, 5555, 6017, 5700, 5571, 5580, 5981, matured in <178 days should be further tested for crop improvement. PCA suggests selection of individual traits that carry high variability due to occurrence and gathering genotypes at the unique positions through scattered diagrams. Moreover, hierarchical cluster analysis advocates geographical distribution of germplasm that is

Formatted: Indent: First line: 0"

Formatted: Font color: Accent 1

independent of origin and gathered genotypes of different origin into same cluster. The high GCV along with heritability and genetic advance depicted wide variation for selection with improving yield traits in lentil cultivars. Ten genotypes including 5664, 5687, 6084, 6062, 6122, 6058, 6087, 5689, 6042, and 6074 were selected based on high yield and recommended for future crop improvement programs.

Declaration

Not applicable

Funding

Not applicable

Conflicts of interest/competing interests

Authors declare no competing interests

Availability of data and material

All of the relevant data is already included

Authors' contributions

Conceptualization: Muhammad Sajjad Iqbal, Abdul Ghafoor, Data curation: Syed Atiq Hussain, Formal analysis: Saba Munir, Ayesha Tahir[2], Methodology: Muhammad Akbar[1], Noshia Arshad[1]. Resources: Muhammad Ahson Khan[3], Muhammad Kashif Ilyas[3]. Software: Muhammad Azhar Ali[1], Syed Atiq Hussain. Supervision: Muhammad Sajjad

Formatted: Adjust space between Latin and Asian text, Adjust space between Asian text and numbers

Formatted: Normal

Iqbal. Validation: Hajra Masood[1], Tahira Ahmad[1], Nazra Shaheen[1], Writing – original draft: Syed Atiq Hussain, Muhammad Sajjad Iqbal. Writing – review & editing: Muhammad Sajjad Iqbal, and Abdul Ghafoor.

Consent to participate

Not applicable

Consent for publication

All authors are willing to publish this manuscript in this journal

References

58. Ates D, Aldemir S, Yagmur B, Kahraman A, Ozkan H, Vandenberg A, et al. QTL mapping of genome regions controlling manganese uptake in lentil seed. G3. 2018; 8: 1409-1416.

59. Ates D. Genetic diversity in lentil landraces revealed by diversity array technology (DArT). Turkish J. Field Crops.2019; 24: 252-260.

60. Scippa GS, Trupiano D, Rocco M, Viscosi V, Di Michele M, Dandrea A, et al. An integrated approach to characterization of two autochthonous lentil (*Lens culinaris*) landraces of Molise (south-central Italy). Heredity. 2008; 101: 136-144.

61. Thavarajah P. Evaluation of chickpea (*Cicer arietinum* L.) micronutrient composition: Biofortification opportunities to combat global micronutrient malnutrition. Food Res. Intern. 2012; 49; 99-104.

Formatted: Adjust space between Latin and Asian text, Adjust space between Asian text and numbers

Formatted: Tab stops: Not at 7.19"

Formatted: Indent: First line: 0"

Formatted: Font: 18 pt

Formatted: Normal

Formatted: Normal, No bullets or numbering

62. Mbasani MJ, Briache FZ, Ennami M, Gaboun F, Benbrahim N, Triqui ZEA, et al. Resistance of Moroccan lentil genotypes to *Orobanche crenata* infestation. J. Crop Improve. 2019; 33: 306-326.

63. Pandey S, Bhatore A. Genetic diversity analysis for quantitative traits in indigenous genotypes of lentil in Madhya Pradesh. J. Pharmacog. Phytochem. 2018; 7, 279-283.

64. Gaad D, Laouar M, Abdelguerfi A, Gaboun F. Collection and agro morphological characterization of Algerian accessions of lentil (*Lens culinaris*). Biodiversitas J. Biol. Divers. 2018; 19: 183-193.

65. Ramírez OAM, Moreno RR, Camara MF. Mineral and trace element content in legumes (lentils, chickpeas and beans): Bioaccesibility and probabilistic assessment of the dietary intake. J. Food Composition and Analysis, 2018; 73: 17-28.

66. Shi L, Arntfield SD, Nickerson M. Changes in levels of phytic acid, lectins and oxalates during soaking and cooking of Canadian pulses. Food Research International. 2018; 107:660-8.

67. FAOSTAT. http://www.fao.org/faostat/en/#data. 2019; Accessed 26 March 2019.

68. Noor R, Khan SM, Ahmad F, Hussain M, Abd-Allah EF, Alqarawi AA, et al. Morpho-agronomic characterization study of *Lens culinaris* genotypes under salt marsh habitat in Swat, Pakistan. Saudi J. Biol. Sci. 2017; 24: 1639-1645.

69. Khan A, Ihsan M, Nisar M, Hazrat A, Ali M, Ul-Haq R, et al. Evaluation of genetic diversity in barley landraces through agromorphological and Biochemical Characterization. Sarhad J. Agri. 2011; 37: 984-992.

70. Ullah J, Hussain SA, Nisar M, Khan W, Khan U, Khan J et al. Biochemical characterization of lentil genotypes for genetic diversity. Plant Cell Biotech. Mol. Biol. 2016; 17: 7-13.

71. Gupta R, Begum SN, Islam MM, Alam MS. Characterization of lentil (*Lens culinaris* M.) genotypes through phenotypic marker. J. Bangladesh Agri. Uni. 2012; 10: 197-204.

72. Cristobal MD, Pando V, Herrero B. Morphological characterization of lentil (*Lens culinaris* Medik.) landraces from Castilla y León, Spain. Pakistan. J. Bot. 2014; 46: 1373-1380.

73. Bilal M, Jamil M, Ali A, Ilyas MK, Ghafoor A. Multivariate analysis of genetic diversity in lentil (*Lens culinaris*) germplasm. Fres. Environ. Bull. 2018. 27: 2151-2159.

74. Mohammed NA, Refay YA, Migdadi HM, AL-Somain BH, Muharram AA, Al-Selwey, et al. Agro-morphological characterization of lentil genotypes in dry environments. Intern. J. Agri. 2019; 22: 1320-1330.

75. Naoura G, Sawadogo N, Atchozou EA, Emendack Y, Hassan MA, Reoungal. Assessment of agro-morphological variability of dry-season sorghum cultivars in Chad as novel sources of drought tolerance. Scientific Rep. 2019; 9: 1-12.

76. Santos CS, Silva B, Valente LMP, Gruber S, Vasconcelos MW. The effect of sprouting in lentil (*Lens culinaris*) nutritional and microbiological profile. Foods. 2020; 9: 400.

77. Iqbal MS, Akbar M. Phenotypic diversity analysis and performance of elite lines of *Brassica napus* L. Bangladesh J. Bot. 50(1): 1-6, 2021.

78. Nisar M, Ghafoor A, Ahmad H, Khan MR, Qureshi AS, Ali H, Islam M. Evaluation of genetic diversity of pea germplasm through phenotypic trait analysis. Pakistan. J. Bot., 40(5): 2081-2086, 2008.

79. Ghafoor A, Arshad M. Multivariate analyses for quantitative traits to determine genetic diversity of blackgram [*Vigna mungo* (L.) Hepper] germplasm. Pakistan. J. Bot., 40(6): 2307-2313, 2008.

80. Tucak M, Popović S, Cupic T, Šimić G, Gantner R, Meglic V. Evaluation of alfalfa germplasm collection by multivariate analysis based on phenotypic traits. Romanian Agri. Res. 26(26), 2009.

81. Iqbal MS, Akbar M, Akhtar S, Fatima S, Ghafoor A. Multivariate analysis and its application for characterizing cowpea landraces. Legume Res. 44 (1): 1-7. 2021.

82. Iqbal Z, Arshad MG, Ashraf M, Mahmood T, Waheed A. Evaluation of soybean [*Glycine Max* (L.) Merrill] germplasm for some important morphological traits using multivariate analysis. Pakistan. J. Bot. 40(6):2323-2328, 2008.

83. Roy S, Islam MA, Sarker A, Malek MA, Rafii MY, Ismail MR. Determination of genetic diversity in lentil germplasm based on quantitative traits. AJCS 7(1):14-21, 2013.

84. Iqbal MS, Qureshi AS, Ghafoor A, Qayyum A. Identification of superior genotypes based on morphological, physiological and agronomic traits in local and exotic cowpea germplasm. Pakistan. J. Bot. 35(1):69-77.2003.

85. Singh RK, Choudhury BD. Biometrical method in quantitative genetic analysis. Kalyani Publishers, Ludhiana, New Delhi, 1985. pp. 54-57.

86. Popat R, Patel R, Parmer D. Variability: Genetic variability analysis for plant breeding research. R package version. http://cran.rproject.org/web/packages/variability/index.html. 2020. Accessed 07 September 2021.

87. Hussain N, Yaqoob M, Rashid A. Genetic competition among lentil (*Lens culinaris*) candidate lines for yield and yield components under rainfed conditions. J. Agri. Res. 2014; 52.

88. Iqbal MS, Ghafoor A, Abbasi FM, Qureshi AS, Ahmad H. Study of nutritional characteristics, mineral nutrients and agro-biodiversity in black cumin (*Nigella sativa* L.) genotypes from Pakistan. Afri. J. Biotech. 2011; 10: 14757-14766.

89. Paliya S, Saxena A, Tikle AN, Singh M, Tilwari A. Genetic divergence and character association of seed yield and component traits of lentil (*Lens culinaris* M.). Adv. Biores. 2015; 6: 53-59.

90. Bhartiya A, Aditya JP, Singh S. Assessment of variability for agro-morphological traits in elite lentil (*Lens culinaris*) lines using multivariate analysis. Indian J. Agri. Res. 2015; 49: 539-543.

91. Ghanem, M. E., Marrou, H., Soltani, A., Kumar, S, Sinclair, TR. Lentil variation in phenology and yield evaluated with a model. Agron. J. 2015; 107: 1967-1977.

92. Lee YJ, Sung JK, Lee SB, Lim JE, Song YS, Lee DB. Yield and free sugar contents of burdock (*Arctium lappa* L.) depending on nitrogen levels. Korean J. Soil Sci. Fertilizer. 2016; 49: 677-681.

93. Roy S, Islam MA, Sarker A, Ismail MR, Rafii MY, Mondal MMA, Malek MA. Morphological characterization of lentil accessions: qualitative characters. Bangladesh J. Bot. 2012; 41: 187-190.

94. Sita K, Sehgal A, Kumar J, Kumar S, Singh S, Siddique K H, Nayyar H. Identification of high temperature tolerant lentil (*Lens culinaris* Medik.) genotypes through leaf and pollen traits. Front. Plant Sci. 2017; 8: 744.

95. Kumar S, Hamwieh A, Manickavelu A, Kumar J, Sharma TR, Baum M. Advances in lentil genomics. In: *Legumes in Omic*. Springer Science + Business Media, New York, USA. 2014; 111-130.

96. Ma Y, Dias MC, Freitas H. Drought and salinity stress responses and microbe-induced tolerance in plants. Frontiers in Plant Sci. 2020; 11: 1750.

97. Pushpavalli R, Zaman-Allah M, Turner NC, Baddam R, Rao MV, Vadez V. Higher flower and seed number leads to higher yield under water stress conditions imposed during reproduction in chickpea. Functional Plant Biol. 2014; 42: 162-174.

98. Nadeem M, Li J, Yahya M, Sher A, Ma C, Wang X, et al. Research progress and perspective on drought stress in legumes: a review. Intern. J. Mole Sci. 2019; 20: 2541.

99. Santos CS, Carbas B, Castanho A, Bronze MR, Serrano C, Vasconcelos MW, Brites C. Relationship between seed traits and pasting and cooking behavior in a pulse germplasm collection. Crop and Pasture Sci. 2018: 69; 892-903.

100. Mirali, M., Purves, R. W., Stonehouse, R., Song, R., Bett, K., Vandenberg, A. (2016). Genetics and biochemistry of zero-tannin lentils. PLoS One, 11(10), e0164624.

101. Dixit GP, Katiyar PK, Singh BB. Characterization of lentil (*Lens culinaris* Medik.) varieties based on morphological traits. J. Food Leg. 2011; 24: 194-197.

102. Dugassa A, Legesse H, Geleta N. Genetic variability, yield and yield associations of lentil (*Lens culinaris* Medic.) genotypes grown at Gitilo Najo, Western Ethiopia. Sci. Tech. Arts Res. J. 2015; 3: 10-18.

103. Depar MS, Soomro NA, Chandio AS, Samoon HA, Meghwar BL, Pathan AK, Kalroo MW. Evaluation of morpho-yield traits and their correlation with seed yield in lentil (*Lens culinaris*) genotypes. Sci. Intern. 2016; 28.

104. Abo-Hegazy SRE, Selim T, El-Emam EAA. Correlation and path coefficient analyses of yield and some yield components in lentil. Egyptian J. Plant Breed. 2012; 203: 1-14.

105. Shrestha J, Subedi S. Improving crop productivity through sustainable intensification. South Asian Res. J. Agri. Fish. 2019; 1: 8-11.

106. Afuape SO, Okocha PI, Njoku D. Multivariate assessment of the agro morphological variability and yield components among sweet potato (*Ipomoea batatas* (L. Lam) landraces. African J. Plant Sci. 2011; 5: 123-132.

107. Benbrahim N, Taghouti M, Zouahri A, Gaboun F. On-farm conservation of Zaer lentil landrace in context of climate change and improved varieties competition. Univ. J. Agric. Res. 2017; 5: 27–38.

108. Idrissi O, Piergiovanni A, Toklu F, Houasli C, Udupa S, De Keyser E, De Riek J. Molecular variance and population structure of lentil (*Lens culinaris* Medik.) landraces from Mediterranean countries as revealed by simple sequence repeat DNA markers: Implications for conservation and use. Plant Genet. Resour. 2018; 16: 249-259.

109. Raza I, Masood MA, Abid S, Farooq M A, Mustafa R. Exploring relationship among quantitative traits of sugarcane varieties using principal component analysis. Sci. Tech. Develop. 2017; 36: 142-146.

110. Nath UK, Rani S, Paul MR, Alam MN, Horneburg B. Selection of superior lentil (*Lens esculenta* M.) genotypes by assessing character association and genetic diversity. The Sci. World J. 2014; 6.

111. Tyagi SD, Khan MH. Studies on genetic variability and interrelationship among different traits in Microsperma lentil (*Lens culinaris* Medik.). J. Agri Biotech Sustain. Devel. 2010; 2: 15.

112. Asghar MJ, Abbas G, Shah TM, Atta BM. Study of genetic diversity in some local and exotic lentil (*Lens culinaris* Medik) genotypes. Pakistan. J. Bot. 2010; 42: 2681-2690.

113. Bicer BT, Sakar D. Heritability of yield and its components in lentil (*Lens culinaris* Medik.). Bulgarian J. Agri. Sci. 2010; 16: 30-35.

114. Sarfraz Z, Shah MM, Iqbal MS. Genetic variability, heritability and genetic advance for agronomic traits among genome donor wheat genotypes. J. Agric. Res. 2016; 54: 15-20.

Formatted: Justified

Supporting information

S1 Table. List of lentil genotypes studied during 2017-18 and 2018-19. Two varieties Markaz 2009 and Punjab 2009 were used as check.

S2 Table. Mean±SE for quantitative traits of 5% best performing lentil genotypes during 2017-18.

S3 Table. Mean±SE for quantitative traits of 5% best performing lentil genotypes during 2018-19.

S4 Table. Principal component analysis for quantitative traits of lentil studied during 2017-18.

S5 Table. Principal Component Analysis for quantitative traits studied during 2018-19.

S1 Fig. Average temperature during lentil crop season October to April 2017-18 and 2018-19 at the experimental station

Formatted: Font: 18 pt

Formatted: Justified, Pattern: Clear

Formatted: Justified

Formatted: Justified, Line spacing: Double

Formatted: Justified, Space After: 0 pt, Line spacing: Double

Response to Reviewers comments

Authors pay thanks to the honorable Reviewers to improve this article a standard MS for wide readership. Further, MS has again reviewed thoroughly and edited sufficiently to make intangible and according to Standard English.

Below is the response to the Reviewers comments please;

Line 141: remove double comma and adjust spacing.

Double commas have been removed and spacing adjusted accordingly, now at line 145 of the manuscript

P11, L 8-9: What do you mean by there in "Short duration genotypes are suggested as early maturing genotypes hence required further trials to see there early maturity cultivar status." Delete the entire sentence.

Deleted at line 197 of the final manuscript without track changes

P12, L5-7: Remove "Traits which possess low variability must be required to address through new collection by broadening existing lentil genetic resources through local as well as exotic sources." There are sentences at the end of each paragraph which are not making any sense. In the result section only point out to important findings. Discussion of results should be in the discussion section.

Authors have removed all of the unnecessary paragraphs as advised by the respectable reviewer, above mentioned lines have been removed as at line 220 of the manuscript

P13, L4-7: Under Pearson correlation matrix: authors indicated that HSW exhibited positive correlation with PS and BY. PH and PS also showed positive correlation with NSP
and DM but Table 4 only shows correlation of SY with other traits in both years. Either you have to show all pairwise correlation in table 4 or don't write about results that are not shown in the table.

Authors have removed the suggested characters and restricted to the traits as recommended.

P 21, L 16: Revise the sentence as "In this study, HSW varied between the years with 3.65 g in 2017-18 and 3.28 g in 2018-19"

Revised accordingly at line 386

P21, L 22: Cooking time not only saves energy and time but also it develops ….
Amended as suggested

P 23, L5: replace & with and
Replaced accordingly at line 420 and also in other paragraphs

P 23, L 9: Revise as “Similar results were observed”

Revised accordingly as suggested in the line 424

Conclusion: tons/hectare versus t/h be consistent throughout

Tons/hectare added as suggested in the line 477.
