# Supplementary material for: Estimating genetic variability among diverse lentil collections through novel multivariate techniques
Source: PLoS One. 2022 Jun 30;17(6):e0269177. doi: 10.1371/journal.pone.0269177 (PMC9246128; doi:10.1371/journal.pone.0269177)
Supplement: Attachment — Submitted filename: PONE-D-21-20067_R1_Reviewer_comments.docx [file pone.0269177.s008.docx]

Please address my comments below.

Abstract

L25 The numbers don’t add up. It is 224 instead of 220??

L36: Define the acronym NP at first mention. Acronyms should be defined at first mention and used consistently. Example: CT was defined on L29 but “cooking time” used on L33 and “CT” on L34. Be consistent!

L37-38: Revise “The PCA revealed 11 components, 38 make convenience selection for individual components for future hybridization”

L40: …showed a perfect relationship OR ..showed that the relationship is perfect.

Introduction

L50-53: “Lentil is the 4th major pulse crop in the world after common bean, pea, and

chickpea, and includes seven taxa with four species such as *L. culinaris, L. nigricans, L.*

*ervoides,* and *L. lamottei*, having two varietal types as macro-sperma (large seeded) and micro-

sperma (small seeded) [4,5].” Revise this sentence which states that all four species have two varietal types but only the cultivated *culinaries* has two types. The others are wild not cultivated.

L62: developing countries

L71: Economically feasible is better expression

L86: Therefore, the objective of this study was to assess…

Materials and Methods

L98: Two hundred and twenty-four lentil

L106: Experiments were laid out in an augmented design. Not lentil seeds!!

L111: Find a better word than “unwinding”. May be studied

L122: and hard seeds (HS) were counted after boiling 100 seeds.

L133: define 0 to 4 and 1 to 3 scales similar to flower ground color above.

L151 change Via through R Studio to using R Studio: Via means through.

Results

L180: full stop after 2.71 g.

L182: change the expression “was ranged” to “ranged” throughout the text

L205: change the expression “both the years” to “both years” throughout the text.

L215: Where is Faisalabad? In Pakistan?

Use of grams and g throughout the paper be consistent!

Page 12 L1: which can be used to describe lentil on…

Page 13: Like, hundred seed weight exhibited… Similarly, hundred seed weight exhibited

Page16: The paragraph below Fig. 3 caption needs revision for grammatical correctness.

Example: scattered plots were (…..)

indicating a way to screen genotypes

therefore, increase in height would result in higher number of seeds per pods

that can help in selection for genotypes

Page 17: The authors keep talking about 220 lines but there are 184 Pakistan +14 Syria + 22 USA +04 Unknown with a total of 224 lines. Where did the 4 lines go? It’s really surprising the authors don’t know the exact number of lines in their 2 year study!

What does C2bii mean on page 17?

Page17: Cluster C7 consisted of only 2 genotypes (5664 Layyah and 6074 Bahawalnagar) which belong to Pakistan. Best performing genotypes were considered in C4 during 2018 and C5 during 2019 due to their performance.

Page 18: Structural Equational Model (SEM): I still don’t get it how cooking time and hard seed count after cooking influence yield? The statistical software are garbage in garbage out. The authors should be cautious in making sense of the data. This doesn’t make sense!

Page 19: with high genetic advance as percentage of mean 79.71 for 2018. What does this mean?

Page 19: Heritability analysis: “The high heritability was observed for BY (0.99) along with high genetic advance as percentage of mean 79.71 for 2018 while for the subsequent year lower pod height 27.94. High genetic gain 2065.13 for the year 2018 was recorded for SY while low genetic gain 0.07 for LPH. Similarly, BY showed high genetic gain 1638.62 for second year and low 0.07 for LPH.” The authors are talking about genetic gain and genetic advance here and in Table 6 but no mention of how they are calculated in the materials and methods section. Genetic gain is response to selection over time. I don’t think it is the right term here. Please revise!

Discussion

Page 21 L9: Put comma following In our study, throughout

Page 22: Although water scarcity affects productivity of legumes at any growth stage, drought during reproductive and grain filling stages (terminal drought) are more critical and usually result in a significant loss of grain yield [39]

Page 22 L10-14: In our study, 5698 (Sialkot, Pakistan) and 6015 (USA), were considered as early maturing genotypes with 161-172 days in 2018 while 24783 (unknown) and 5561 (Sanghar, Pakistan) were found early maturing 161-172 days during 2019 which is more appealing as compared to previous reports of 168-190 days (cite the reference of the previous report here).

Page 22 2^nd^ paragraph: It is evident that seed yield is directly proportional to number of seeds per pod, higher number of seed per pod will result in higher seed yield.

Page 22: lowest cooking time 10 mint should be minutes (or m).

Page 23: Grey ground color of testa and orange-red cotyledon color with grey color of pattern on testa was observed in most lines.

Page 24: use of & instead of and in the text

Page 25: Revise “It is also infer that presence of genotypes…” not grammatically correct. It can be inferred that…

Page 26: Most of the agro-morphological traits had fairly higher heritability [18]
