# Supplementary material for: Estimating genetic variability among diverse lentil collections through novel multivariate techniques
Source: PLoS One. 2022 Jun 30;17(6):e0269177. doi: 10.1371/journal.pone.0269177 (PMC9246128; doi:10.1371/journal.pone.0269177)
Supplement: Attachment — Submitted filename: Response to Reviewers.docx [file pone.0269177.s009.docx]

**Response to Reviewer’s comments**

Abstract

**L25 The numbers don’t add up. It is 224 instead of 220??**

Current collection is of 220 genotypes representing Pakistan (178), Syria (14), USA (22) and 6 are of unknown origin. Exact number included throughout the text.

**L36: Define the acronym NP at first mention. Acronyms should be defined at first mention and used consistently. Example: CT was defined on L29 but “cooking time” used on L33 and “CT” on L34. Be consistent!**

Corrected as advised.

**L37-38: Revise “The PCA revealed 11 components, 38 make convenience selection for individual components for future hybridization”**

We revised the text as suggested and excluded that deem unfit.

The PCA revealed a considerable reduction in components for selection of suitable genotypes with desired traits that be utilized for future lentil breeding.

**L40: …showed a perfect relationship OR ..showed that the relationship is perfect.**

Revised as commented.

Structural Equational Model (SEM) for SY based on covariance studies indicated the perfect relationship among variables.

**Introduction**

**L50-53: “Lentil is the 4th major pulse crop in the world after common bean, pea, and**

**chickpea, and includes seven taxa with four species such as *L. culinaris, L. nigricans, L.***

***ervoides,* and *L. lamottei*, having two varietal types as macro-sperma (large seeded) and micro-sperma (small seeded) [4,5].” Revise this sentence which states that all four species have two varietal types but only the cultivated *culinaries* has two types. The others are wild not cultivated.**

Sentence revised as like following

Lentil is the 4^th^ major pulse crop in the world after common bean, pea, and chickpea, and included seven taxa with four species such as *L. culinaris, L. nigricans, L. ervoides,* and *L. lamottei*, the only cultivated species (*L. culinaris*) have two varietal types as macro-sperma (large seeded) and micro-sperma (small seeded) [4,5].

**L62: developing countries**

Corrected

**L71: Economically feasible is better expression**

Replaced.

It is not economically feasible to reduce biotic stressors by utilizing pesticides, herbicides and to reduce abiotic stresses by supplementing soil to meet plant demand through reclamation and enhancing fertilizer, as well as by improving irrigation systems [13].

**L86: Therefore, the objective of this study was to assess…**

Text revised as stated as;

The current studies used several statistical techniques to unwind the hidden potential of genetic diversity in the available lentil genetic resources and to assess variability in agro-morphological traits for consecutively two years. Identified novel genotypes would be the base material for future lentil breeding program.

**Materials and Methods**

**L98: Two hundred and twenty-four lentil**

Corrected as the number is of 220 and replaced throughout the text where applicable. Ambiguity of 224 is removed.

**L106: Experiments were laid out in an augmented design. Not lentil seeds!!**

Corrected.

Experiments were laid out in an augmented design within 11 blocks and each block consisted of 20 genotypes along with two check varieties, *viz.*, Markaz 2009 and Punjab 2009 after every twenty lines [16].

**L111: Find a better word than “unwinding”. May be studied**

Revised as; actual genetic diversity may be studied.

The trial was conducted under usual agricultural practices without applying any herbicide, fungicide, pesticide, or fertilizer so that the actual genetic diversity may be studied.

**L122: and hard seeds (HS)** **were counted after boiling 100 seeds.**

Correction made as per suggestion.

After harvesting, biological yield (BY), seed yield (SY), 100-seed weight (100-HSW), cooking time (CT), and hard seeds (HS) were counted after boiling 100 seeds.

**L133: define 0 to 4 and 1 to 3 scales similar to flower ground color above.**

Well defined all of the traits accordingly.

At late vegetative stage, SSP (rated as 0; absent and 1; present), LS (3; small, 5; medium, and 7; large) and LP (0; absent, 3; slight, and 7; dense) were observed. At early reproductive stage, GCF (1; white color, 2; white with blue lines, 3; blue, 4; violet, 5; pink, and 6; other) was observed. At late reproductive stage, PP (0; absent and 1; present) was observed. At maturity stage, PD (0; none, 3; low, 5, medium, and 7; high) and PSh (0; none, 3; low, 5, medium, and 7; high) were surveyed. Tendril length (1; rudimentary tendril and 2; prominent tendril) was also observed during late reproductive stage. After harvesting, CPT (0; absent, 1; olive, 2; grey, 3; brown, 4; black.), CC (1; yellow color, 2; orange-red, and 3; olive green) and GCT (1; green, 2; grey, 3; brown, 4; black, and 5; pink) was observed.

**L151 change Via through R Studio to using R Studio: Via means through.**

Changed and the whole paragraph is revised in view of the suggestion.

using R Studio

Results

**L180: full stop after 2.71 g.**

Sentence paragraphed revised**.**

SY in 2018-19 as compared to SY of 20117-18. Seed yield ranged from 5 to 85 g in 2017-18 and 3 to 76 g during 2018-19, respectively. Likewise, 6084 belonged to Narowal, Pakistan produced the highest SY in both years. The lowest SY was recorded in genotype 5475 originated from Gujranwala, Pakistan during 2017-18, whereas the genotype 5494 (Okara, Pakistan) and 5475 (Gujranwala, Pakistan) were the lowest in SY during 2018-19. Genotypes 6052 (Syria), 5583 (Muzaffargarh, Pakistan), and 5556 (Hyderabad, Pakistan) produced better SY during both years. Overall performance of genotypes was better during the first year as compared to the subsequent year that could be attributed due to better environmental conditions during second year, however, genotypic trend remained similar during both years indicating evidence of heritable heritability among genotypes.

**L182: change the expression “was ranged” to “ranged” throughout the text**

Changed throughout the text as suggested.

**L205: change the expression “both the years” to “both years” throughout the text.**

Revised according to suggestion.

**L215: Where is Faisalabad? In Pakistan?**

Yes, Faisalabad is in Pakistan and Pakistan included in the text along the cities representing as origin.

**Use of grams and g throughout the paper be consistent!**

g used throughout the text**.**

**Page 12 L1: which can be used to describe lentil on…**

Revised accordingly

**Page 13: Like, hundred seed weight exhibited… Similarly, hundred seed weight exhibited**

Sentence revised accordingly

The 100-SW was positively correlated with BY, while BY was positively correlated with PH.

**Page16: The paragraph below Fig. 3 caption needs revision for grammatical correctness.**

**Example: scattered plots were (…..)**

**indicating a way to screen genotypes**

**therefore, increase in height would result in higher number of seeds per pods**

**that can help in selection for genotypes**

The whole paragraph revised accordingly

Genotypes (5506, 5729, 5510, 5672, 5636, 5581, 5491, 5483) at the upper right corner were grouped on the basis of 100-SW, BY and SY indicating productive genotypes while in lower right corner genotypes (5472, 5512, 5479, 5486, 5650, 5600, 5531) reflected variation due to PH, NP, and HS indicating close association of PH with NP and HS. Therefore, increase in height would result in higher number of seeds per pods that can help in selection for genotypes. Similarly, at the upper left side of the scattered plot, genotypes (6080, 6043, 5653, 6043, 6002, 6060, and 5677) showed varying responses to CT, SP, and PS that can help in selection for genotypes with desirable CT contributed by NSP and PS which affects possible CT. While on the other hand, in lower left-side genotypes 23787, 24787, 23779, 5742, and 5712 were placed with the highest variability in DM and LPH (Fig. 4). It can infer that DM has a significant impact on LPH.

**Page 17: The authors keep talking about 220 lines but there are 184 Pakistan +14 Syria + 22 USA +04 Unknown with a total of 224 lines. Where did the 4 lines go? It’s really surprising the authors don’t know the exact number of lines in their 2 year study!**

The exact number of collection is 220 as stated in point 1. Misperception was due to typographic error. We apologize for misunderstanding.

**What does C2bii mean on page 17?**

C2bii represented the sub-cluster under cluster C2. The rephrased the sentences to avoid complication of the figure.

Cluster C1 consisted of 37 genotypes while cluster C2 consisted of 115 genotypes. Cluster C3 consisted of 12 genotypes whereas clusters C4 and C5 consisted of only 1 (24783) and 4 genotypes (6084, 6058, 6062, and 6122) respectively. Cluster C6 consisted of 49 genotypes and cluster C7 consisted of 2 genotypes. Best performed genotypes were considered in C3 during 2017-18 and C5 during 2018-19 due to their performance as SY, BY, PS, NSP, CT, and HS. Cluster C2 belongs to 100-SW >1.19 producing genotypes during 2017-18 while those genotypes which produced 100-SW >1.19 during 2018-19 were grouped into cluster C3. Cluster C2 also consisted of those genotypes which had PH >55.1 and DM >170 in both years. Cluster C1 consisted of genotypes having SY > 40 or near to 40g during both years.

**Page17: Cluster C7 consisted of only 2 genotypes (5664 Layyah and 6074 Bahawalnagar) which belong to Pakistan. Best performing genotypes were considered in C4 during 2018 and C5 during 2019 due to their performance.** ).

Sentence revised accordingly

Best performed genotypes were considered in C3 during 2017-18 and C5 during 2018-19 due to their performance as SY, BY, PS, NSP, CT, and HS.

**Page 18: Structural Equational Model (SEM): I still don’t get it how cooking time and hard seed count after cooking influence yield? The statistical software are garbage in garbage out. The authors should be cautious in making sense of the data. This doesn’t make sense!**

SEM analysis revised to make uniformity of the data while cooking time removed as suggested.

**Table 5.** Structural equations of model representing regression for agronomic traits studied during 2017-18 and 2018-19.

| **SY** | **100SW** | **BY** | **PH** | **LPH** | **NSP** | **PS** | **DM** | **NP** |
| --- | --- | --- | --- | --- | --- | --- | --- | --- |
| Z-values | 2.12 | 0.27 | 0.02 | 0.43 | 3.69 | 5.43 | -0.04 | 2.21 |
| P-values | 0.10 | 0.09 | 0.00 | 0.01 | 0.81 | 0.0 | 0.0 | 0.04 |

SY = 2.16*100-SW + 0.27*BY + 0.025*PH + 0.0.43*LPH + 1.826*NSP + 6.34*PS - 0.039*DM + 2.298*NP

SY, seed yield; BY, biological yield; PH, plant height; LPH, lower pod height; PS, pod size; DM, days to maturity; NP, number of pods; NSP, number of seeds per pod.

**Figure 8.** Structural equational model for seed yield (SY)

**Page 19: with high genetic advance as percentage of mean 79.71 for 2018. What does this mean?**

Following description has been included

It indicates predominance of additive gene action for these traits under prevailing conditions. Further, high genetic advance along high heritability estimates provides ample opportunity for most suitable selection of traits for future application.

**Page 19: Heritability analysis: “The high heritability was observed for BY (0.99) along with high genetic advance as percentage of mean 79.71 for 2018 while for the subsequent year lower pod height 27.94. High genetic gain 2065.13 for the year 2018 was recorded for SY while low genetic gain 0.07 for LPH. Similarly, BY showed high genetic gain 1638.62 for second year and low 0.07 for LPH.” The authors are talking about genetic gain and genetic advance here and in Table 6 but no mention of how they are calculated in the materials and methods section. Genetic gain is response to selection over time. I don’t think it is the right term here. Please revise!**

Agreed with the honorable reviewer’s observation and we have included genetic advance calculation procedure/formula under material & methods section while excluded the genetic gain.

Discussion

**Page 21 L9: Put comma following in our study, throughout**

Comma added.

**Page 22:** **Although water scarcity affects productivity of legumes at any growth stage, drought during reproductive and grain filling stages (terminal drought) are more critical and usually result in a significant loss of grain yield [39]**

Sentence revised as suggested above.

Variation in native, as well as exotic collections, was observed for both years. Although water scarcity affects productivity of legumes at any growth stage, drought during reproductive and grain filling stages (terminal drought) are more critical and usually result in a significant loss of SY.

**Page 22 L10-14:** **In our study, 5698 (Sialkot, Pakistan) and 6015 (USA), were considered as early maturing genotypes with 161-172 days in 2018 while 24783 (unknown) and 5561 (Sanghar, Pakistan) were found early maturing 161-172 days during 2019 which is more appealing as compared to previous reports of 168-190 days (cite the reference of the previous report here).**

Overall sentence has been revised and desired reference has been included.

(16)

**Page 22 2^nd^ paragraph: It is evident that seed yield is directly proportional to number of seeds per pod****, higher number of seed per pod will result in higher seed yield.**

Sentence has been revised accordingly

It is evident that SY is directly proportional to NSP, and in current studies, NP ranged from 2-3 in 2017-18 and 1-3 in 2018-19.

**Page 22: lowest cooking time 10 mint should be minutes (or m).**

Amended.

**Page 23: Grey ground color of testa and orange-red cotyledon color with grey color of pattern on testa was observed in most lines.**

Sentence revised accordingly

~~Grey ground color of testa and orange-red cotyledon color with grey color of pattern on testa was observed in majority. Various markets demand specific seed coat colors and/or patterns. We attempted to determine the percentage of color of testa, cotyledon color and color of pattern.~~

**Page 24: use of & instead of and in the text (need to be discussion)**

**Authors preferred to use and instead of & throughout the text**

**Page 25: Revise “It is also infer that presence of genotypes…” not grammatically correct. It can be inferred that…**

Sentence has been revised (need to be check it grammatically)

It can be inferred that presence of genotypes having different origins in the same cluster might be close association as common ancestors but on contrary presenting in different clusters also showed variation in genetic material.

**Page 26: Most of the agro-morphological traits had fairly higher heritability [18]**

Sentence has been revised accordingly

Most of the agro-morphological traits had fairly higher heritability [18].
