# Supplementary material for: Estimating genetic variability among diverse lentil collections through novel multivariate techniques
Source: PLoS One. 2022 Jun 30;17(6):e0269177. doi: 10.1371/journal.pone.0269177 (PMC9246128; doi:10.1371/journal.pone.0269177)
Supplement: Attachment — Submitted filename: Response to Reviewers comments.docx [file pone.0269177.s010.docx]

Response to Reviewers comments

Authors pay thanks to the honorable Reviewers to improve this article a standard MS for wide readership. Further, MS has again reviewed thoroughly and edited sufficiently to make intangible and according to Standard English.

Below is the response to the Reviewers comments please;

**Line 141: remove double comma and adjust spacing.**

Double commas have been removed and spacing adjusted accordingly, now at line 145 of the manuscript

**P11, L 8-9: What do you mean by there in “Short duration genotypes are suggested as early maturing genotypes hence required further trials to see there early maturity cultivar status.” Delete the entire sentence.**

Deleted at line 197 of the final manuscript without track changes

P12, L5-7: Remove “Traits which possess low variability must be required to address through new collection by broadening existing lentil genetic resources through local as well as exotic sources.” There are sentences at the end of each paragraph which are not making any sense. In the result section only point out to important findings. Discussion of results should be in the discussion section.

Authors have removed all of the unnecessary paragraphs as advised by the respectable reviewer, above mentioned lines have been removed as at line 220 of the manuscript

**P13, L4-7: Under Pearson correlation matrix: authors indicated that HSW exhibited positive correlation with PS and BY. PH and PS also showed positive correlation with NSP
and DM but Table 4 only shows correlation of SY with other traits in both years. Either you have to show all pairwise correlation in table 4 or don’t write about results that are not shown in the table.**

Authors have removed the suggested characters and restricted to the traits as recommended.

**P 21, L 16: Revise the sentence as “In this study, HSW varied between the years with 3.65 g in 2017-18 and 3.28 g in 2018-19”**

Revised accordingly at line 386

**P21, L 22: Cooking time not only saves energy and time but also it develops ….**
Amended as suggested

**P 23, L5: replace & with and**
Replaced accordingly at line 420 and also in other paragraphs

P 23, L 9: Revise as “Similar results were observed”

Revised accordingly as suggested in the line 424

Conclusion: tons/hectare versus t/h be consistent throughout

Tons/hectare added as suggested in the line 477.
