# Supplementary material for: Estimating genetic variability among diverse lentil collections through novel multivariate techniques
Source: PLoS One. 2022 Jun 30;17(6):e0269177. doi: 10.1371/journal.pone.0269177 (PMC9246128; doi:10.1371/journal.pone.0269177)
Supplement: Attachment — Submitted filename: Response to Reviewers.docx [file pone.0269177.s012.docx]

Response to Reviewers Comments

Reviewer #1: My comments are added to the attached pdf file. I advise the authors to take their time to correct grammatical and punctuation issues. I tried to point out some of them but it is better to have it proofread by native speakers of English or use online platforms such as grammarly for addressing these issues.

Following points were rectified as per advice of the respectable Reviewer by considering both MS Word and PDF files while English language corrections were made throughout the text where suggested which can be seen in revised manuscript with track changes.

MS finalized, where line numbering is now changed due to addition, deletion and inclusion.

Furthermore, MS have been proofread by native speakers of English Dr. Danish Ibrar and Dr. Shahbaz Khan to whom names are acknowledged.

Moreover, online “Grammarly” source have also been consulted to improve the MS accordingly.

Line 30	NSP corrected

Line 45	NPS to NSP corrected

Line 68	‘’due to this physician strongly suggest’’ Sentence removed and modified

Line 91	‘’Current study engaged several statistical techniques’’ sentence Replaced with ‘’this study used ‘’

Line 92	‘’ and aiming “replaced with aimed.

Line 105	comma (,) added

Line 106	including removed

Line 107	sentence modified as per suggestion

Line 122	On a plant basis removed and shifted after data after data

Line 129	NP replaced with NPP

Line 128	‘’after boiling 100 seed’’ sentence removed

Line 129	Sentence has been revised as cooking time in minutes was recorded after boiling of hard unsoaked seed to soften in distilled water.

Line 151	Indicating replaced with indicates

Line 152	Broad sense replaced as broad-sense

Line 159	Broad sense replaced as broad-sense

Line 162	Broad sense replaced as broad-sense

Line 167	Broadened replaced with broaden

Line 166	Comma (,) added after over all

Line 178-180	removed

Line 184	Whereas has been excluded

Line 188	NP corrected as NPP

Line 193-194	Removed and embedded

Line 194	‘’Following’’ deleted

Line 195	‘’Broadened’’ has been removed

Line 205	removed

Line 211 NP	corrected as NPP

Line 129-130	‘’HSW was positively correlated with BY, in return BY was also positively correlated with 235 PH. Desirable combinations described high potential of the germplasm that can be utilized in 236 future crop improvement programs through selection’’ sentence removed

Line 231-240	‘’and much better about evaluated genotypes’’ sentence removed

Line 240	‘’Second year’’ added

Line 246	“Furthermore, scattered plot diagram “changed to ‘’to scatter plot’’

Line 250	Other the genotypes’’ removed

Line 251	Of the germplasm removed

Line 272	‘’While on the other hand, at lower left side’’ sentence removed

Line 285-286	sentence removed

Line 288	Sentence modified

Line 295	‘’as’’ replaced with ‘’ for’’

Line 292	‘’further more ‘’ corrected

Line 312	observed to be’’ modified

Line 327	‘’Figure-7’’ added

Line 328	‘’Found to be’’ was added

Line 328	‘’the traits under study’’ removed

Line 334	It replaces with this

Line 334	Furthermore added

Line 335	Along with corrected

Line 341	Table-6	SP corrected as NSP

Line 341	NP corrected as NPP

Line 344	The removed

Line 344	Scientist replaced with plant breeders

Line 348-349	‘’It is 356 always to be desirable by breeders to choose high yielding genotypes exhibit highest yield 357 potential to develop varieties or to carry out multi-location trials for varying environments [31] ’’ sentence modified

Line 356	overall performance’’ sentence modified

Line 357	to be deleted

Line 364	‘’develops’’ sentence modified

Lines 366-367	‘’There 375 were additionally several environmental factors which influence crop performance as water 376 scarcity affects productivity of legumes at growth stage ‘’ sentence modified

Line 368	are’’ changed to ‘’is’’

Line 370	‘’present ‘’ deleted

Line 370	‘’Genotypes’’ removed

Line 373	Further’’ removed

Line 373	‘as well against‘’ deleted

Line 379	‘’during cold season after adopting breeding strategy for lentils

Line 380	sentence modified and ‘’showed wide variation during both years’’ deleted

Line 382	‘’Further more’’ corrected

Line 383	‘’because’’ added before save energy

Line 383-384	Revised/Rectified

Line 386	‘’over all‘’ sentence revised

Line 387	‘’further for trials and breeding programs ‘’ revised

Line 389	Evidenced’’ deleted

Line 392	‘’Seed’’ removed

Line 396	‘’It’’ replaced with ‘’they’’

Line 394	‘’LP (leaf hairs)’’ corrected

Line 405	‘’Further more’’ revised

Line 406	Understanding of ‘’ revised

Line 412	‘’someone should be very careful about sowing time and its stage’’ revised

Line 423- 424	Revised

Line 427 -428	‘’therefore, this component is also suggested for further use” revised

Line 478	genotypic coefficient of variance (GCV), has already mentioned in the table 6

Line 431-572	comprehensively edited as per English language improvement suggestion by respectable Reviewer and expert See file Manuscript with track changes April 24, 2022

Discussion and Conclusion section improved as per suggestion of the expert and their names are included under acknowledgment. See file Manuscript with track changes April 21, 2022

References were rechecked again to remove any discrepancy.

Figures and supporting material rechecked, revised where fit and data reported in the MS has been counter checked again.
