# Supplementary material for: Estimating genetic variability among diverse lentil collections through novel multivariate techniques
Source: PLoS One. 2022 Jun 30;17(6):e0269177. doi: 10.1371/journal.pone.0269177 (PMC9246128; doi:10.1371/journal.pone.0269177)
Supplement: Attachment — Submitted filename: Response to Reviewer comments.docx [file pone.0269177.s007.docx]

Reviewer #1

**1; In this study, the authors evaluated 220 lentil collections for various quantitative and qualitative traits using augmented design in 2018 and 2019. However, sufficient detail of the field layout and statistical analysis of the raw data was not provided. How many blocks were used?**

The detail of field layout and statistical analysis is added as mentioned below;

Eleven blocks were used in this study. Each block consisted of 20 genotypes and two check varieties, viz., Markaz 2009 and Punjab 2009 were planted after every twenty lines. For statistical analysis ten plants were sampled radomly from each line/genotypes. Morphological and agronomical traits were recorded on indvidual plant basis during 2018 and 2019 [16]. Recorded data of agronomical traits were averaged and mean data were subjected to analyse for descriptive statistics, frequncy distribution, Pearson correlation, PCA, cluster pattern analysis, structure equational modling. While broad sense heritablity was estimated by considereing 2018 as replication 1 and 2019 as replication 2. The descriptive statistics including minimum values, maximum values, range, mean, variances, standered deviation,, cofficient of variances and standered error of mean were estimated through statistical softwere (XLStat 2019).

As the qualitative traits did not changed from both years so combined frequency distribution were estimated [16]. To find the relationship among agronomical traits Pearson correlation was computed with XLStat 2019, as well for PCA. Dendrograms were constructed based on Eucladian distance through RStudio. The structural equation modling was drawn through LISREL (LISREL Student Version 9.2<https://lisrel-for-windows-student.software.informer.com/9.2/>) to estimate multiple regression that was indicating contribution of quantitative traits toward yield. The genetic and phenotypic variances and broad sense heritabity were estimated by formmula suggested by Singh and Chaudhary [51] through online source PBSTAT (http://pbstat.com/ppb/).

**2; Each genotype was grown on a 5m row but it is not indicated if all rows were arranged in a single block or multiple blocks were used?**

Eleven blocks were used in this study. Each block consisted of 20 genotypes and two known cultivars, viz., Markaz 2009 and Punjab 2009 were planted as check (control) after every twenty lines.

**3;Augmented design is used when the number of entries are large. Entries are unreplicated but checks should be replicated and are used to adjust entry means for soil gradient differences. The authors did not indicate if they used adjusted means. This is critical because some of the quantitative traits are highly influenced by the environment and the observed differences may be due to the location effect.**

The adjusted mean values were used. Sentence is added in statistical analysis for clarification about adjusted mean and soil was homogenously distributed as we conducted and provided soil analysis (Line 113).

**4;The background statement states that low yield of lentil in Pakistan is mainly because of adaptation of low yielding local genotypes in different environments due to low genetic variability. However, the best genotypes with the highest seed yield, early maturity, and lowest cooking time are from Pakistan which contradicts the first statement.**

Before collection, evaluation and characterization there were no prior information about yield potential. After conducting subject mentioned analysis we come to know that current germplasm representing best performance is mainly based on collections belonged to Pakistan. We assumed outcome of this trial will lead to explore them under different environments for future varietal development.

**5; Overall, I found several grammatical and punctuation errors which made reading the paper difficult. I advise the authors to address the comments below and resubmit it to local/regional journals as it may be more useful to lentil breeders in and around Pakistan.**
We rectified the points raised, our study is of international significance because of germplasm collection representing USA, Syria, Pakistan, and few are of unknown origin. Therefore it has scope of international use and wide readability.

**6;Lines 31-32: if (Pak) stands for Pakistan then write in full to make it consistent with the one writes on Line 30.**

We replaced the word Pak with Pakistan throughout text

**7;Lines 37-39: In conclusion, 12 genotypes including 5664, 5687,… Markaz 2009, and Punjab 2009 were selected on …..**

In conclusion, 12 genotypes including 5687, 6084, 6062, 6122, 6058, 6087, 5689, 6042, 6074, Markaz 2009 & Punjab 2009 were selected on the basis of best performance especially seed yield to other traits viz., biological yield, plant height, pod size, cooking time and maturity time etc.

**8;Line 42: Lens culinaris Medik.**

Full stop is added at the end of Medik.

**9;Line 43: It was domesticated**

Changed as suggested

**10;Line 45: throughout the Mediterranean Basin**

‘The’ is included before Mediterranean basin

**11;Line 46: Lentil is the 4th major pulse crop in the world after common bean, pea, and chickpea, and includes seven taxa and four species such as L. culinaris,**

Sentence revised as desired

**12;Line 50: Lentils are consumed due to their third-highest level**

Lentil is consumed

**13;Line 62: Why is the reference in superscript as opposed to within square brackets? Be consistent throughout.**

Corrected according to format of Plos One references

**14;Lines 67-69: The sentence “For combating effects of biotic and abiotic stresses by…. cautiously practicable [12]” is not clear what the authors are trying to say. These are practicable. Are the authors referring to Pakistan?**

Corrected as;

It is not economically possible to reduce biotic stressors by utilizing pesticides and herbicides, and to reduce abiotic stresses by supplementing soils to meet plant demands through reclamations and enhancing fertiliser, as well as by improving irrigation systems [13].

**15;Moreover, reference no 12 was cited here but it has nothing to do with the sentence. Please double check if it is the right reference?**

Amended, the correct reference is [13]

**16;Line 79: quantitative and qualitative traits [20].**

Corrected

**17;Line 97: I suggest moving Table 1 into supplementary material.**

Moved as supplementary material

**18;Line 100: during 2018 and 2019. It is known that these are two consecutive years. Revise similar expressions throughout.**

Revised: during 2018 and 2019

**19;Line 101: Presentation of Figure 1 in material and methods section is unnecessary. Here describe methods only Eg. Monthly temperatures during these years were recorded at the experimental station. Present the figure in the results section.**

Figure now moved to results section (P17).

**20;Line 101: Reference 16 is superscript again???**

Corrected: [16]

**21;Line 125: color of testa was observed???**

Corrected
**22;Table 1: Table caption should be placed above the table and figure caption should be below the figure. This should be supplemental file. Do not present tables and figures in Materials and methods section.**

Amended as desired.

**23;Statistical analysis: XLstat 2019 was used to analyze quantitative and qualitative traits data” The method is not detailed enough.**

Detail is provided now

**25; What type of analysis performed?**

Recorded data of agronomical traits were averaged and mean data were subjected to analyse for identification of best genotypes, descriptive statistics, frequency distribution, pearson correlation, PCA, hiererchical clster analysis, structure equational modling and broad sense heritablity.

**26;The authors used augmented design with two checks interspersed every 20 lines? But in the statistical analysis there is no mention if entry values were adjusted relative to the checks.**

These values are adjusted values as detail is given at point 1.

**27;	Proper ANOVA need to be performed for quantitative traits. Are the frequency distribution, Pearson correlation, and PCA performed based on adjusted entry means???**

ANOVA performed and values of significant traits added in Table 2. Yes, the frequency distribution, Pearson correlation, and PCA performed based on adjusted entry means for each year.

**28;	How is Heritability estimated?**

It is estimated from mean values of traits for each genotype during both years.

**29;	Is it broad-sense or narrow-sense?**

It is broad sense heritability

30;	**Which formula was used to estimating heritability? It appears that the authors divided VG/VP but there is no mention how VG is computed.**

Singh and Chaudhary [28] method was followed as provided below

Where GV and PV is the genetic variances and phenotypic variances respectively gms= genetic mean square

ems= error mean square

r= number of replications

EV= envirenmental variances

h^2^= broad sens heritability

References used for heritability [29]

Popat R, Patel R, Parmer D. Variability: Genetic variability analysis for plant breeding research. R package version. <http://cran.rproject.org/web/packages/variability/index.html>. 2020. Accessed 07 September 2021.

We revised whole table by performing analysis again and showing more parameters to increase understanding of the data.

**31;	The trial was conducted for 2 years but no mention of how this computation was performed. These are critical questions that need to be addressed but the methods lack sufficient detail to assess if the right approach was followed.**
Included following information in the main text as already mentioned under point 1;

Ten plants were labeled as sample from each genotypes and agronomical traits were recorded on indvidual plant basis during 2018 and 2019 [16] Recorded data of agronomical traits were averaged and mean data were entered into excel 2016 separatly for 2018 and 2019. Both years data was separatly subjected to analyse for identification of best genotypes, descriptive statistics, frequncy distribution person correlation PCA, dendrogram, structure equational modling while broad sens heritablity was estimated by considereing 2018 as replication 1 and 2019 as replication 2. The descriptive statistics including minimum values, maximum values, range , mean, variances, standered deviation,, cofficient of variancecs and standered error of mean were estimated through statistical softwere (XLstat 2019). As the qualitative traits did not changed from both years so combined frequency distribution for both years were estimated [16]. To find the relation ship among agronomical traits person correlation were computed with XLstat 2019, same staitiscal tool was used for PCA. Dendrogram was constracted based on eucladian distance through RStodio. The structural equation modle was constrcuted through LISREL (LISREL Student Version 9.2 <https://lisrel-for-windows-student.software.informer.com/9.2/>) to estimate multiple regression that was indicating contribution of quantitative traits toward yield.

**32;	Results: Presentation of results should focus on key findings. The authors do not need to repeat everything that can be easily seen in tables.**

Done according to suggestion

**33;	Moreover, if proper ANOVA was conducted and there was no significant effect of year then the two-year means can be presented instead of showing each year data separately which made the paper unnecessarily lengthy.**

ANOVA performed and found significant effects which are now included in Table 2.

**34;	Table 2: What is V? SP is shown in the footnote but not indicated in the table??**

Corrected: V is variance.

SP is present in row number 7

35;	**What is VC? Do you mean CV?**

Corrected, yes, it is CV

The authors should have taken time to clean up such mistakes before submitting this manuscript.
Rectified mistakes as advised throughout the text.

**36;	Table 6 and 7: As these are qualitative traits, they are almost similar between the two years. Why is it necessary to show almost identical data in two tables?**

Table 6 removed

**37;	Tables 9 and 10: What are Fs? Each table should be standalone and descriptions of acronyms should be added in footnotes. Same for Table 11.**

F represents factors

Description of acronyms added in footnotes for table 9 and 10

**38;	Table 11: The authors are trying to predict yield using cooking time as independent variable which doesn’t make sense. How is cooking time contributing to yield? they presented correlation of agronomic traits with cooking time. Correlation does not imply causation.**

Agreed with respectable Reviewer and we removed cooking time

**39;	Page 24-24: “Values less than 1.00……. individual differences”. This sentence is not accurate. Heritability is a dynamic number and values less than 1 does not necessarily mean genes did not contribute to the phenotypic variance.**

Agreed, heritability analysis is revised after excluding qualitative traits. Few more statistical parameters like genetic advance and genetic gain are included.

**40;	Page 26 line 30, Page 27 line 1: different in text citation. Be consistent and refer to the journal citation and reference formats. There are several other similar cases and need revision throughout.**

Corrected page 26 line 30 and page 27 line 1. Revised throughout the text

Reviewer #2:

**The paper by Hussain et al. focus on the important topic of legume germplasms characterization to feed international breeding programs and promote technological advances in plant alternatives to the 5 major crops in which the World currently relies for protein and carbohydrates provision.**

**The quality of the English writing is very poor, starting from the first sentence of the abstract (not abstracts as in L21) and with repeated writing problems such as misplaced capital letters and words lacking. This requires professional english correction.**
English language corrections and editing is performed by local English expert, Dr. Behzad Anwar, Department of English, UOG

**The amount of Tables and Figures is too dense, and authors should revise information to be put as supplementary data or presented in alternative ways. This is also true for the paper in general, I believe that authors must revise the content and make the paper more straightforward and lighter, since it is too extensive in its current form.**

Authors consider suggestions, removed extra text and moved the tables as supplementary material

**In general the reference section has older papers and has to be updated.**

New and updated references are included now

**L62,101; P31 – reference in the wrong format.**

**Corrected according to format**

**L76 - more recent references, such as, DOI: 10.1002/ppp3.10158;** [**https://doi.org/10.3390/foods9040400**](https://doi.org/10.3390/foods9040400)

Suggested reference added

**L82 - delete 'in the near past'; this is a current topic**

Deleted  **L94 - Change Plants to Plant**

Changed

**L103 - these two check varieties are already described for which traits?**

Markaz 2009; suitable for barani and irrigated areas of the plains and Pothwar region. Fimilar as high yielding, drought tolerant and lodging resistant.

Punjab 2009; descried for high yield, moderately resistant to rust and stem rot, tolerant to lodging

**L105 - soil characterization is required**

Soil type used was analysed by Soil Science Laboratory, National Agricultural Research Centre (NARC), Islamabad and was Nabipur series, coarse loamy, mixed, hyperthemic, and udic ustochrepts. Added at Line 113.

**From page 8 forward there is no numbering of the lines.**

We make text continue with line numbering

**In the results section, grams appear after the numbers both without spacing or between parenthesis or in full word (e.g p11). The correct form is after the number with spacing e.g. 3.2 g. Correct throughout the whole text.**

Corrected throughout text.

**P9 'Lentils characterized during first year showed better performance as compared to subsequent year.' this is discussion, not results description.
P11 - replace & with and**

Moved to discussion section; Lentils characterized during first year showed better performance as compared to the subsequent year.
  **Table 4/5 are not readable and the authors should present this in an alternative, more friendly reader way. In the titles is not best performed, but best performing**

**Revised as suggested.**

**P16/18/22/24 - no need to refer the program again, it's in M&M**

**Revised**

**Table 9 & 10 can be supplementary data and the results description regarding PCA should focus only on the first two components.**

Table 9 and 10 moved to supplementary data. The result description of PCA revised according to suggestion.

**P26 - Jaleel et al, 2009 is not in the correct form**

Corrected

**P27 - Pushpavalli et al, 2014 also**

Corrected

**P27 - please correct the sentence 'Another aspect of lentils is lowest cooking time considered a** **favourite trait for** [**consumers.in**](http://consumers.in/) **the current studies, lowest cooking time 10 mint' from punctuation to words misspelling**

Corrected

The discussion on seed coat coloring and the genetic background is quite interesting but I feel it lacks integration in the context of the author's study, as they did not look at this dimension.

Appropriate text has been included

**The definition of PCA is excusable. This is for academic partners who know this method.**

Unnecessary text has been removed

**P29 - And not only in Pakistan or Turkey, for example:** [**https://doi.org/10.1071/CP18205**](https://doi.org/10.1071/CP18205)

Included

**P30 - Once again, punctuation errors should be covered throughout the entire document: 'Moreover, other traits like number of pods. Lower pod height and plant height were also placed in F3, F4, and F5, respectively.'**

Rectified

**In the conclusion section, authors shouldn't just summarize the main findings again, that is for the Abstract section; here, the main findings should be commented on further, with the industry and farmers sector perspective, showing the importance of the study for them. Would the authors advise farmers to just use the best performing genotype and forget the rest?**

Conclusion revised as suggested.

**Are these 12 genotypes well adapted to different edaphoclimatic regions, or just in the research center site?**

These 12 genotypes were selected on best performance out of 220 after conducting two years of evaluation and characterization under same edaphoclimatic conditions therefore recommended for the area having similar features like Photohar, arid and semi-arid regions. Moreover, these genotypes have potential to become stable varieties after trials.

We revised carefully and now suggested 09 genotypes for further crop improvement program.
